# Health inequities in SARS-CoV-2 infection, seroprevalence, and COVID-19 vaccination: Results from the East Bay COVID-19 study

**DOI:** 10.1371/journal.pgph.0000647

**Published:** 2022-08-15

**Authors:** Cameron Adams, Mary Horton, Olivia Solomon, Marcus Wong, Sean L. Wu, Sophia Fuller, Xiaorong Shao, Indro Fedrigo, Hong L. Quach, Diana L. Quach, Michelle Meas, Luis Lopez, Abigail Broughton, Anna L. Barcellos, Joan Shim, Yusef Seymens, Samantha Hernandez, Magelda Montoya, Darrell M. Johnson, Kenneth B. Beckman, Michael P. Busch, Josefina Coloma, Joseph A. Lewnard, Eva Harris, Lisa F. Barcellos

**Affiliations:** 1 School of Public Health, University of California, Berkeley, California, United States of America; 2 Institute for Health Metrics and Evaluation, University of Washington, Seattle, Washington, United States of America; 3 University of Minnesota Genomics Center, University of Minnesota, Minneapolis, Minnesota, United States of America; 4 Vitalant Research Institute, San Francisco, California, United States of America; KEMRI-Wellcome Trust Research Programme Nairobi, KENYA

## Abstract

Comprehensive data on transmission mitigation behaviors and both SARS-CoV-2 infection and serostatus are needed from large, community-based cohorts to identify COVID-19 risk factors and the impact of public health measures. We conducted a longitudinal, population-based study in the East Bay Area of Northern California. From July 2020-March 2021, approximately 5,500 adults were recruited and followed over three data collection rounds to investigate the association between geographic and demographic characteristics and transmission mitigation behavior with SARS-CoV-2 prevalence. We estimated the populated-adjusted prevalence of antibodies from SARS-CoV-2 infection and COVID-19 vaccination, and self-reported COVID-19 test positivity. Population-adjusted SARS-CoV-2 seroprevalence was low, increasing from 1.03% (95% CI: 0.50–1.96) in Round 1 (July-September 2020), to 1.37% (95% CI: 0.75–2.39) in Round 2 (October-December 2020), to 2.18% (95% CI: 1.48–3.17) in Round 3 (February-March 2021). Population-adjusted seroprevalence of COVID-19 vaccination was 21.64% (95% CI: 19.20–24.34) in Round 3, with White individuals having 4.35% (95% CI: 0.35–8.32) higher COVID-19 vaccine seroprevalence than individuals identifying as African American or Black, American Indian or Alaskan Native, Asian, Hispanic, two or more races, or other. No evidence for an association between transmission mitigation behavior and seroprevalence was observed. Despite >99% of participants reporting wearing masks individuals identifying as African American or Black, American Indian or Alaskan Native, Asian, Hispanic, two or more races, or other, as well as those in lower-income households, and lower-educated individuals had the highest SARS-CoV-2 seroprevalence and lowest vaccination seroprevalence. Results demonstrate that more effective policies are needed to address these disparities and inequities.

## Introduction

The first confirmed severe acute respiratory syndrome coronavirus 2 (SARS-CoV-2) infection in the California Bay Area was reported on February 28, 2020 [1]. Measures to prevent transmission were implemented soon after and included shelter-in-place orders, mask mandates, business and school closures, and social distancing recommendations. Despite these measures, spikes in reported infections occurred from July to September 2020 and December 2020 to February 2021 [2].

Local public health and healthcare systems experienced major challenges in preventing infections, identifying COVID-19 cases, and ensuring adherence to transmission mitigation strategies. Furthermore, disparities and inequities in COVID-19 risk and outcomes by race and ethnicity and socioeconomic factors such as income and education have been observed in the United States including in the San Francisco Bay Area [3–7]. A detailed understanding of the effectiveness of transmission mitigation behaviors and sociodemographic factors that contribute to the disproportionate impact of COVID-19 in vulnerable communities is critical. Public health and policy directives aimed at controlling ongoing transmission, developing future prevention strategies, and targeting health disparities and inequities must be evidence-based. Large population-representative cohorts with individual-level data on social and behavioral factors [8] associated with COVID-19 and SARS-CoV-2 infection and serostatus [9–13] are needed.

To address this need, we investigated individual-level characteristics and mitigation behaviors that contributed to SARS-CoV-2 seroprevalence, self-reported infection, and viral infection, and other outcomes in a large, population-based sample of over 5,500 individuals from 12 East Bay cities in Northern California followed longitudinally over three time periods (July-September 2020, October-December 2020, and February-March 2021). The East Bay region was chosen for study because it is densely populated, and both racially and ethnically diverse. We estimated the population-adjusted prevalence of SARS-CoV-2 outcomes and differences by age, sex, race/ethnicity, ZIP code, and other demographic strata as well as the effect of transmission mitigation behavior on SARS-CoV-2 prevalence with Bayesian multilevel regression and poststratification (MRP) models [14,15].

## Methods

### Recruitment and participants

Recruitment and selection of study participants was completed using a screening phase followed by a longitudinal study phase with three rounds of data collection. In the screening phase, all residential addresses within the East Bay cities and communities of Albany, Berkeley, El Cerrito, El Sobrante, Emeryville, Hercules, Kensington, Oakland, Piedmont, Pinole, Richmond, and San Pablo (~307,000 residential households) were mailed an invitation to complete a consent form and screening questionnaire. Within a household, the individual aged 18 years or older with the next upcoming birthday from the date of postcard receipt, was eligible to participate. Additional eligibility criteria included living within the study region, willingness to provide biospecimens and questionnaire responses, ability to read and speak English or Spanish, and having internet access and an email address.

The target sample size was 5,500 participants per study round. The distribution of racial and ethnic identification in the screening questionnaire responses was more White and non-Hispanic than the region population. To obtain a sample that resembled the racial and ethnic proportions in the 2018 American Community Survey (ACS) for the study region, we ranked assigned ranks for order of inclusion to all eligible individuals who responded to the screening questionnaire. Black and Hispanic individuals were ranked the highest followed by individuals identifying as Asian, Pacific Islander, American Indian or Alaskan Native, two or more races, or other. Order of inclusion for White individuals was randomly sampled. In Round 1, individuals ranked between 1 and 5,500 were offered study enrollment. Those who declined to enroll were replaced with next highest ranked individuals who had not been offered study entry. In subsequent rounds, individuals who had participated in the previous round(s) were offered participation in the next round. If participation was declined, individuals from the pool of participants who had not participated in a study round were invited. Approximate dates for each round were July-September 2020, October-December 2020, and February-March 2021. Further information in S1 File.

### Ethics statement

All participants provided their formal written informed consent for the screening phase. All participants in the study phase provided their formal written informed consent for each study round. The study was approved by the University of California, Berkeley Committee on Protection of Human Subjects (Protocol #2020-03-13121).

### Study procedures

At the start of each round, eligible participants were invited to participate. Those who agreed to participate received a kit via FedEx containing materials for self-collection of biospecimens, pre-paid return shipping labels, and instructions to complete an online-administered questionnaire at the same time as biospecimen collection.

### Questionnaire

The questionnaire addressed sex, gender, age, race/ethnicity, income, employment, physical and mental health, as well as symptoms potentially related to COVID-19 within the previous 2 weeks, and SARS-CoV-2 testing outside of the study (S2 File). Participants were also asked about transmission mitigation behaviors including physical distancing practices, close contacts with others, and mask wearing. Questionnaires were available in English or Spanish.

### SARS-CoV-2 viral and antibody testing

Anterior nasal nare swabs for viral RNA testing and dried blood spots (DBS) for antibody testing were collected from participants at each study round. Quantitative reverse transcription PCR (RT-qPCR) was used to identify SARS-CoV-2 viral infection. Three tests were used to assess anti-SARS-CoV-2 antibodies in DBS: Ortho VITROS Anti-SARS-CoV-2 Total IgG (ORTHO-IgG) and spike IgG ELISA (ELISA-IgG) targeted antibodies against the SARS-CoV-2 spike protein (indicating prior natural infection or vaccination), and Roche-NC (ROCHE-NC) Total IgG targeted antibodies against the nucleocapsid (NC) protein (indicating prior natural infection only) [16]. Before COVID-19 vaccines were available in the study region during rounds 1 and 2, detection of antibodies against the SARS-CoV-2 spike protein was considered evidence of SARS-CoV-2 infection. During Round 3, vaccinations were widely available, therefore detection of antibodies against the NC protein were considered evidence of SARS-CoV-2 infection, while detection of antibodies against the spike protein were considered evidence of SARS-CoV-2 infection or COVID-19 vaccination (S1 Fig) [17–20]. Further information on viral and antibody testing in S3 File.

The sensitivity and specificity of the antibody assays used in this study are reported in Wong et al and in S5 Table [16]. Briefly, in round 1, using a threshold of S/C>1 for evidence of anti-Spike antibodies, the sensitivity (Se) and specificity (Sp) of the ORTHO-IgG assay was 80.6% [95% CI: (64.0–91.8)] and 100% [95% CI: (63.1–100)], respectively. In rounds 2 and 3, we implemented a serial testing strategy, using the ORTHO-IgG assay as the screening test (S/C>0.7) and the ELISA-IgG assay as the confirmatory test. In these rounds, the sensitivity and specificity of the ORTHO-IgG assay 88.9% [95% CI: (73.9–96.9)] and 100% [95% CI: (63.1–100)], respectively. The sensitivity and specificity of the ELISA-IgG assay was 97.2% [95% CI: (88.7–99.9)] and 100% [95% CI: (87.7–1)], respectively. In round 3, the ROCHE-NC assay was used as a confirmatory test for presence of NC antibodies. The sensitivity and specificity were 86.7% [95% CI: (69.3–96.2)] and 97.9% [95% CI: (94.8–99.4)], respectively.

### SARS-CoV-2 outcomes

The following outcomes were investigated in each round: (1) cumulative SARS-CoV-2 antibody positivity, (2) participants’ self-reported history of SARS-CoV-2 positivity from testing outside the study, (3) and a surveillance definition of “probable COVID-19 case” derived from self-reported symptoms and close contact with infected individuals [21], and (4) viral SARS-CoV-2 positivity from RT-qPCR testing of nasal swabs (S3 File). We also investigated antibodies induced by COVID-19 vaccination only in Round 3. Cumulative SARS-CoV-2 antibody positivity was defined as having detectable antibodies in the current and/or previous round(s). DBS samples that tested negative for anti-NC antibodies and positive for anti-spike antibodies in Round 3 were considered to have antibodies from COVID-19 vaccination alone. Self-reported COVID-19 viral positivity prevalence was defined as the proportion of participants reporting a positive viral test among all participants who reported being tested within a study round. Probable COVID-19 case prevalence was defined as the proportion of participants identified as a probable COVID-19 case among all participants who provided valid responses within a study round. Viral positivity prevalence was defined as testing positive by RT-qPCR from nasal swab samples.

## Statistical analysis

### Population-adjusted seroprevalence and other SARS-CoV-2 outcomes

Bayesian MRP was used to estimate population-adjusted cumulative seroprevalence, self-reported SARS-CoV-2 viral positivity prevalence at each study round, and “probable COVID-19” prevalence at each study round (See S4 File for further information on MRP and statistical methods). MRP is a regression-based method for estimating population and sub-population averages from survey data that has been shown to perform better than survey weighting, particularly with sparse data [14,15].

In addition to estimation of regional prevalence of our outcomes, we estimated prevalence within demographic groups and geographic areas in the study region. Variables of interest were gender, age, race, Hispanic ethnicity, income, education, household size, and ZIP code. We used a method described by Leeman and colleagues to generate a synthetic population for poststratification using data from the 2018 American Community Survey (ACS) and the Public Use Microdata Sample [15]. Poststratification was done using binary sex because gender is not reported by the ACS. Race and ethnicity were combined into a single variable to reduce the number of poststratification strata. This procedure yielded 44,640 strata.

At each study round, binary SARS-CoV-2 outcomes were modeled as a function of geographic and demographic characteristics using multilevel logistic regression models. Participant sex was included as a fixed effect. Vectors of random intercepts were defined for each category of race/ethnicity, age, education, income, household size, and ZIP code and two-way interactions between ZIP code, race/ethnicity, educational attainment, income, and age. To improve estimation of geographic effects we allowed for spatial correlations using the modified Besag-York-Mollié model and included the proportion of Spanish speaking households within the ZIP code of residence from the ACS as a fixed effect [22].

### SARS-CoV-2 outcome prevalence and measures of association

We report populated-adjusted prevalence of SARS-CoV-2 outcomes across the study region and within geographic and demographic groups of interest. To calculate prevalence estimates, posterior distributions of the relevant poststratification stratum were aggregated. We also estimated prevalence differences (PD) and prevalence ratios (PR) for the association between populated-adjusted SARS-CoV-2 outcomes and race/ethnicity, education, and sex. For each parameter of interest, the mean of the posterior distribution was the point estimate, and the 95% credible interval (CI) was the 2.5% and 97.5% quantiles of a posterior distribution.

### SARS-CoV-2 test-kit bias corrected seroprevalence

We estimated cumulative SARS-CoV-2 seroprevalence at each study round adjusted for the sensitivity and specificity of the SARS-CoV-2 antibody testing assays used in each study round (S5 Table).

### Transmission mitigation behavior analyses

In each round, participants were asked about physical distancing practices, recent close contacts with others, mask wearing, and other behaviors and activities that might affect the risk of SARS-CoV-2 infection (S5 File). We classified participants into two behavior categories, “high-risk” and “low-risk”, with those responses using latent class analysis [23]. Crude associations between behavior categories and characteristics such as sex, age, race/ethnicity, education, and income were assessed with *χ*^2^ tests. Associations between high-risk vs. low-risk behavior and within round SARS-CoV-2 seroprevalence and self-reported test positivity were estimated using the MRP model described above with random intercepts for behavior categories and interactions between the behavior categories and ZIP code, age, race/ethnicity, education, and income.

Statistical analyses were completed in R 4.0.2. NIMBLE was used to implement MRP models [24]. Descriptions of MRP methods and code are provided in at github.com/adams-cam/ebcovid_prev.

## Results

### Enrollment and characteristics of study participants

**Fig 1:**
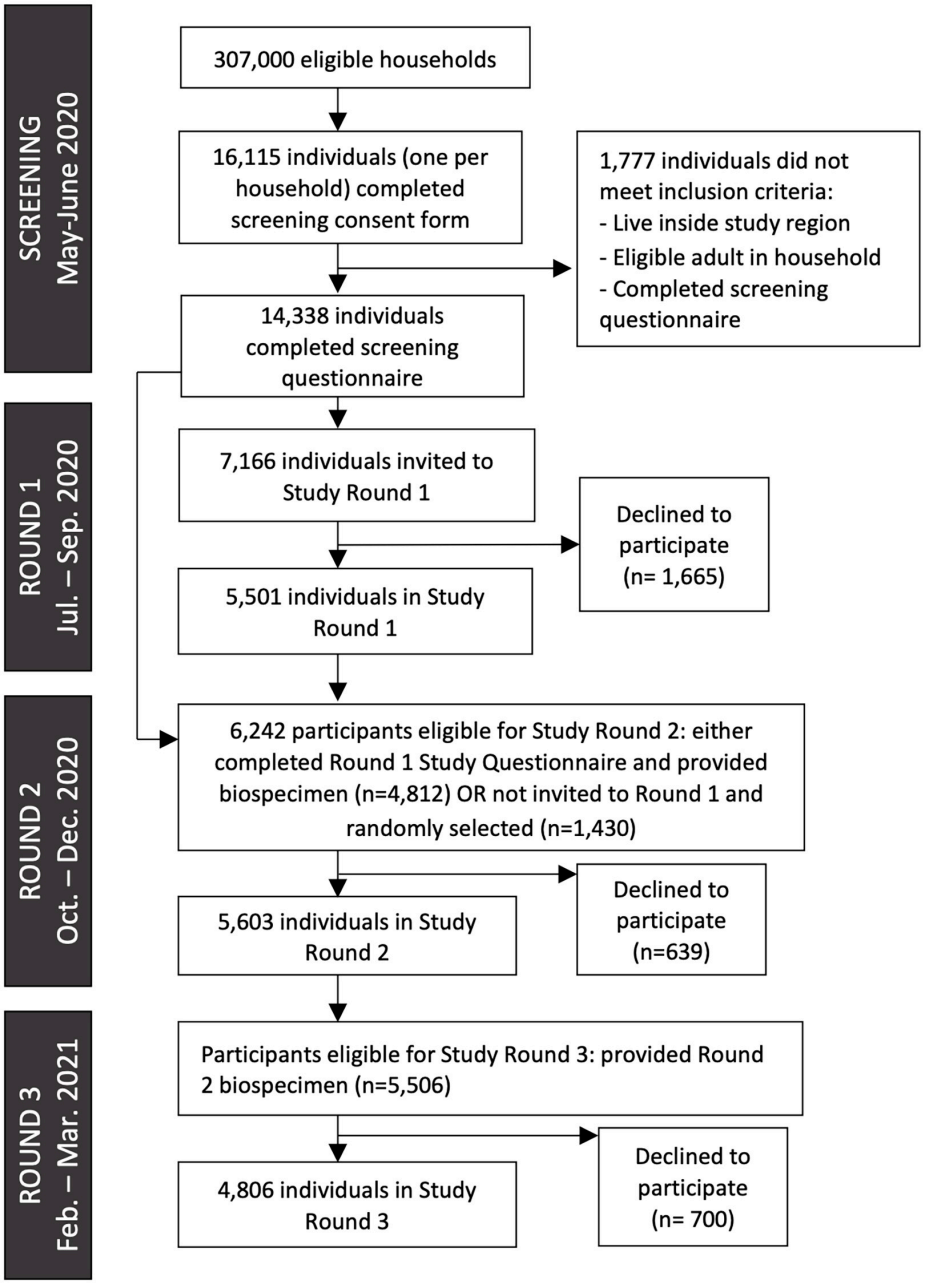
Study flow chart.

Of the 16,115 residents who consented and completed the screening procedures between May-July 2020, 1,777 did not satisfy inclusion criteria and were excluded (Fig 1). Characteristics of participants are presented in Table 1 and S1 Table. Participation rates were high (Round 1: 76.8%, Round 2: 89.8%, and Round 3: 87.3%), and participants identified predominantly as female (~63%). Those aged 45–64 years were the largest age group of participants across all study rounds (ranging from 37.3% to 39.4%). Most participants identified as White (52.5% to 63.3%), followed by Asian/Pacific Islander (13.9% to 15.7%), Hispanic (11.0% to 15.6%), two or more races (6.9% to 9.1%), African American or Black (3.0% to 4.9%), and Native American/Alaska Native or other (1.7% to 2.2%). Approximately 4,750 participants (86.3% of Round 1 participants) completed all three study rounds.

**Table 1:** Characteristics of participants at each round of the study compared to study region population.

	PS Strata^a^ %	Round 1 no. (%)	Round 2 no. (%)	Round 3 no. (%)
Invited to round		7166	6242	5506
Invited from previous study round			4,812 (77.1)	5,506 (100)
Additional invitees from screening pool			1,430 (22.9)	0
Total participants in study round		5501 (76.8)	5603 (89.8)	4806 (87.3)
Gender				
Female		3451 (62.7)	3509 (62.6)	3040 (63.3)
Male		1996 (36.3)	2036 (36.3)	1721 (35.8)
Other		51 (0.9)	52 (0.9)	40 (0.8)
Missing		3 (0.1)	6 (0.1)	5 (0.1)
Sex				
Female	51.8	3502 (63.7)	3559 (63.5)	3080 (64.1)
Male	48.2	1996 (36.3)	2042 (36.4)	1725 (35.9)
Missing		3 (0.1)	2 (0)	1 (0)
Age				
18–29	24.2	476 (8.7)	378 (6.7)	286 (6)
30–44	29.0	1768 (32.1)	1656 (29.6)	1330 (27.7)
45–64	30.0	2052 (37.3)	2141 (38.2)	1895 (39.4)
65–74	10.2	928 (16.9)	1077 (19.2)	977 (20.3)
75+	6.6	275 (5)	345 (6.2)	313 (6.5)
Missing		2 (0)	6 (0.1)	5 (0.1)
Race/ethnicity				
African American/Black	17.4	268 (4.9)	180 (3.2)	146 (3)
American Indian/Alaska Native/Other	0.5	123 (2.2)	114 (2)	84 (1.7)
Asian/Pacific Islander	20.3	862 (15.7)	763 (13.6)	668 (13.9)
Hispanic	22.4	860 (15.6)	637 (11.4)	527 (11)
Two or more races	3.8	500 (9.1)	400 (7.1)	334 (6.9)
White	35.6	2886 (52.5)	3503 (62.5)	3041 (63.3)
Missing		2 (0)	6 (0.1)	6 (0.1)
Educational attainment				
College degree or more	47.0	4912 (89.3)	5101 (91)	4404 (91.6)
Less than college degree	53.0	552 (10)	496 (8.9)	399 (8.3)
Missing		37 (0.7)	6 (0.1)	3 (0.1)
Current annual household income, USD				
<$100K	62.9	2081 (37.8)	2075 (37)	1779 (37)
≥$100K	37.1	3192 (58)	3347 (59.7)	2866 (59.6)
Missing		228 (4.1)	181 (3.2)	161 (3.3)

Abbreviations: PS, Poststratification; USD, United States Dollar.

^a^ Population percentages from synthetic poststratification tables generated from American Community Survey (ACS) and Public Use Microdata Sample data. Each cell contains the percentage of population age >18. Gender is not available in ACS data.

Of those who completed the questionnaire, 87.3%, 95.3%, and 96.6% provided DBS and 93.6%, 98.1% and 98.5% provided nasal swabs in rounds 1, 2, and 3, respectively (Table 2). Antibodies against the SARS-CoV-2 spike protein were detected in 29 (0.6%) and 33 (0.6%) of DBS in rounds 1 and 2, respectively. In Round 3, NC antibodies from natural infection alone were detected in 84 participants (1.8% of 4,806) and spike antibodies from natural infection or vaccination were detected in 1,452 participants (31.3% of 4,806). Viral infection was detected in less than three nasal swabs in each round. The proportion of participants reporting both being SARS-CoV-2 tested outside the study and testing positive increased over the study period: 10/1,030 (1.0%) participants reporting a positive COVID-19 result in Round 1, 19/2,059 (0.9%) in Round 2, and 53/1,892 (2.8%) in Round 3. Few participants met the criteria for being a COVID-19 probable case (<0.5%) (S2 Table).

**Table 2:** Prevalence of SARS-CoV-2 related outcomes among participants per study round.

	Round 1	Round 2	Round 3
Biospecimen collection period	Jul. 4, 2020—Sep. 1, 2020	Oct. 10, 2020—Dec. 16, 2020	Feb. 11, 2021—Mar. 20, 2021
N	5501	5603	4806
Provided DBS, no. (%)	4801 (87.3)	5340 (95.3)	4641 (96.6)
Provided nasal swab, no. (%)	5148 (93.6)	5499 (98.1)	4735 (98.5)
SARS-CoV-2 spike antibody status^a^, no. (%)			
Non-reactive	4641 (96.7)	5275 (98.8)	3157 (68.0)
QNS	131 (2.7)	31 (0.6)	32 (0.7)
Reactive	29 (0.6)	33 (0.6)	1452 (31.3)
SARS-CoV-2 nucleocapsid antibody status ^b^, no. (%)			
Non-reactive			4181 (90.1)
QNS			376 (8.1)
Reactive			84 (1.8)
SARS-CoV-2 viral status, no. (%)			
Invalid/Inconclusive	4 (0.1)	5 (0.1)	7 (0.1)
Not Detected	5143 (99.9)	5491 (99.9)	4725 (99.8)
Positive 2019-nCoV	1 (0.0)	3 (0.1)	3 (0.1)
Self-reported positive COVID-19 test, no. (%)			
No	1020 (18.5)	2059 (36.7)	1892 (39.4)
Yes	10 (0.2)	19 (0.3)	53 (1.1)
Not tested	4471 (81.3)	3525 (62.9)	2861 (59.5)
Probable COVID-19 case^c^, no. (%)			
No	4800 (87.3)	4938 (88.1)	4419 (91.9)
Yes	21 (0.4)	10 (0.2)	8 (0.2)
Not reported	680 (12.4)	655 (11.7)	379 (7.9)

Abbreviations: DBS, dried blood spot; QNS, quantity not sufficient.

^a^ Antibody assays specific to spike antibodies detect presence of antibodies induced by SARS-CoV-2 natural infection or vaccination.

^b^ Antibody assays specific to nucleocapsid antibodies detect presence of antibodies induced by SARS-CoV-2 natural infection.

^c^ Defined by the Council for State and Territorial Epidemiologists.

### Population adjusted SARS-CoV-2 outcome prevalence in study region

Population adjusted SARS-CoV-2 seroprevalence, self-reported COVID-19 test positivity, and probable COVID-19 cases are reported in Table 3. Overall, populated-adjusted SARS-CoV-2 natural infection seroprevalence was low across the study region: Round 1 (July-September 2020) 1.03% (95% CI 0.50–1.96), Round 2 (October-December 2020 1.37% (0.75–2.39), and Round 3 (February-March 2021) 2.18% (95% CI 1.48–3.17). In Round 3 the populated-adjusted seroprevalence of COVID-19 vaccination was 21.64% (95% CI: 19.2, 24.34). Models incorporating sensitivity and specificity of the antibody assays yielded lower SARS-CoV-2 seroprevalence estimates in Round 1, similar estimates in Rounds 2 and 3, and a higher estimate of COVID-19 vaccine seroprevalence in Round 3. Population adjusted self-reported test positivity to SARS-CoV-2 was similar in Rounds 1 (1.11%, 95 CI: 0.39–2.40) and 2 (1.29%, 95% CI: 0.55, 2.17) to seroprevalence estimates and increased to 4.58% (95% CI: 2.56–7.64) in Round 3. Population-adjusted prevalence of being a COVID-19 probable case was <1% across all study rounds (S2 Fig).

**Table 3:** Crude and population-adjusted prevalence (%) and 95% credible intervals of SARS-CoV-2 outcomes within the study region.

	Round 1	Round 2	Round 3
SARS-CoV-2 Outcome	Prev.% (95% CI)	Prev. % (95% CI)	Prev. % (95% CI)
SARS-CoV-2 Ab+ NI^a^			
Crude	29/4670 = 0.62%	48/5949 = 0.81%	110/5991 = 1.83%
Pop-adjusted^b^	1.03 (0.50, 1.96)	1.37 (0.75, 2.39)	2.18 (1.48, 3.17)
Pop-adjusted + Test-bias adjusted^c^	0.63 (0.04, 2.00)	1.02 (0.17, 2.40)	2.19 (1.49, 3.18)
SARS-CoV-2 Ab+ V^d^			
Crude			1369/4608 = 29.7%
Pop-adjusted^b^			21.64 (19.2, 24.34)
Pop-adjusted + Test-bias adjusted^c^			27.56 (22.84, 33.55)
Self-reported test positivity^e^			
Crude	10/1030 = 0.97%	19/2078 = 0.91%	53/1945 = 2.73%
Pop-adjusted^b^	1.11 (0.39, 2.40)	1.29 (0.55, 2.71)	4.58 (2.56, 7.64)
Probable COVID-19 case^f^			
Crude	21/4821 = 0.44%	10/4948 = 0.2%	8/4427 = 0.18%
Pop-adjusted^b^	0.59 (0.26, 1.19)	0.64 (0.17, 1.75)	0.65 (0.17, 1.77)

Abbreviations: Ab, antibody; NI, natural infection; Prev., prevalence; V, vaccination.

^a^ Cumulative antibody prevalence from natural infection by SARS-CoV-2.

^b^ Population-adjusted prevalence estimated using multilevel regression and poststratification models.

^c^ Population-adjusted prevalence estimated using multilevel regression and poststratification models adjusted for antibody assay sensitivity and specificity.

^d^ Cumulative prevalence of antibodies from COVID-19 vaccination.

^e^ Within round prevalence of self-reported COVID-19 test positivity.

^f^ Within round prevalence of COVID-19 probable case.

### SARS-CoV-2 seroprevalence and infection vary by geographic area

**Fig 2:**
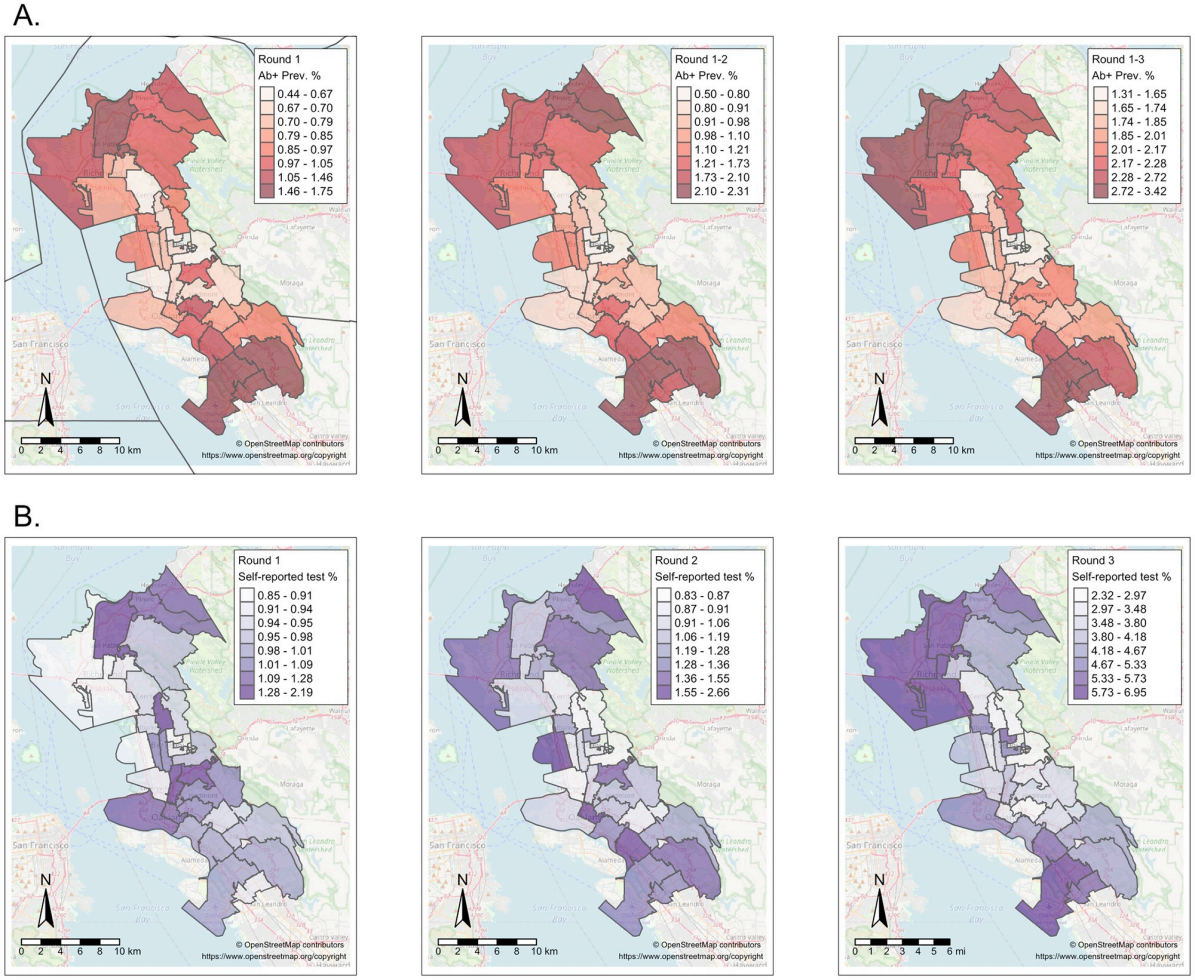
Population-adjusted prevalence of SARS-CoV-2 outcomes within study region ZIP codes. (A) Cumulative seroprevalence of SARS-CoV-2 antibodies to natural infection. (B) Prevalence of self-reported COVID-19 test positivity across the study region. Data collected in three rounds: Round 1, July-September 2020; Round 2, October-December 2020), and Round 3, February-March 2021. Base map and data from OpenStreetMap and OpenStreetMap Foundation (https://www.openstreetmap.org/copyright).

There was evidence for spatial differences in both populated-adjusted seroprevalence and self-reported test positivity (Fig 2). The northern areas (Richmond, San Pablo, Pinole, and Hercules) and southern areas (East Oakland) of the study region had higher seroprevalence than Berkeley, El Cerrito, and North/Downtown Oakland. Self-reported test positivity was also higher in the northern and southern areas. These trends were consistent across study rounds.

### Population adjusted seroprevalence and self-reported test positivity prevalence within subgroups

**Fig 3:**
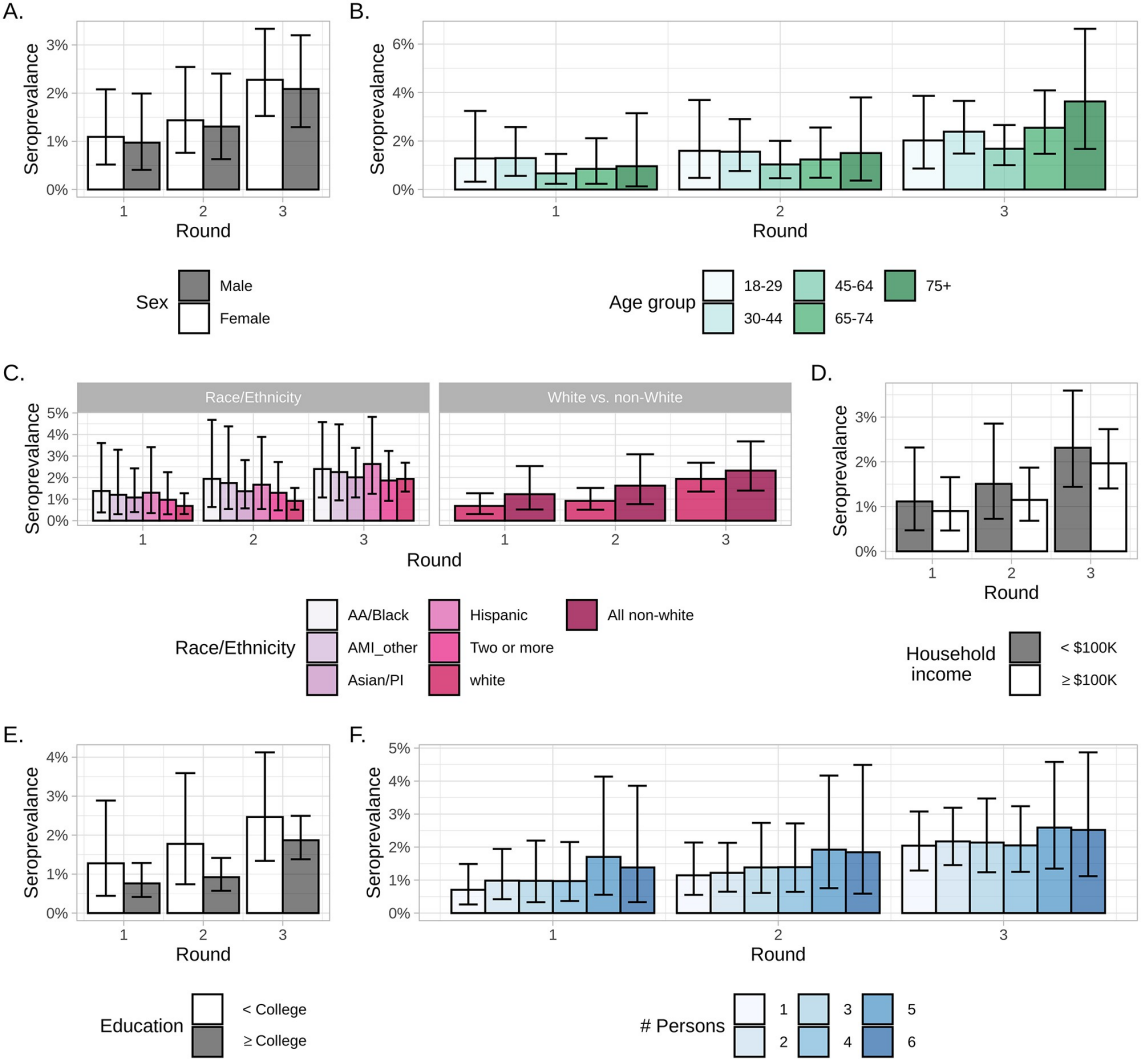
Cumulative populated-adjusted seroprevalence of SARS-CoV-2 antibodies to natural infection among demographic groups. A) Sex, B) Age, C) Race/ethnicity, D) Income, E) Educational attainment, and F) Household size. Abbreviations: AA, African American; AMI, American Indian or Alaskan Native; PI, Pacific Islander.

Estimated populated-adjusted seroprevalence within demographic subgroups is reported in Fig 3. Cumulative seroprevalence increased across all demographic groups over the study period. Non-white individuals consistently showed evidence for higher seroprevalence than White individuals, specifically those identifying as African American or Black and Hispanic. We also found evidence for higher prevalence among those with less than a college degree compared to those with a college degree and those with a household income less than $100,000 compared those with household income greater than $100,000. In addition, there appeared to be a small threshold effect in household size, where those in households with five or more persons had a higher seroprevalence than those in households with four or less persons. There were no consistent trends in prevalence by age or sex. Similar relationships were seen between demographic groups and self-reported test prevalence (S3 Fig).

### COVID-19 vaccination seroprevalence differs by race/ethnicity and age

In Round 3, populated-adjusted COVID-19 vaccination seroprevalence was lower among individuals identifying as African American or Black, American Indian or Alaskan Native, Asian, Hispanic, two or more races, or other compared to White individuals (White vs. Non-White, PD: -4.35%, 95% CI: (-9.32, -0.35), Table 4, S4 Fig). The difference between racial/ethnic groups was largest among older participants; non-White individuals had a lower COVID-19 vaccination seroprevalence compared to White individuals among those aged between 65–74 (PD: -11.67%, 95% CI: (-20.98, -2.49)) and those aged 75 or older (PD: -10.15%, 95% CI: (-22.25, 0.43)).

**Table 4:** Population-adjusted prevalence of antibodies from COVID-19 vaccination in Round 3 within race/ethnicity and age groups and prevalence differences between non-White and White individuals.

	Within group	Non-White individuals compared to White individuals
	Prev. % (95% CI)^a^	PD % (95% CI)^b^
Race/ethnicity		
White	25.33 (23.14, 27.65)	Ref
African American/Black	19.69 (13.47, 26.13)	-5.65 (-12.17, 0.76)
American Indian/Alaska Native/Other	18.87 (12.32, 25.95)	-6.46 (-13.3, 0.51)
Asian/Pacific Islander	21.34 (17.24, 26.13)	-4 (-8.4, 0.93)
Hispanic	18.64 (13.74, 24.4)	-6.69 (-11.83, -0.81)
Two or more races	15.74 (11.85, 19.94)	-9.6 (-13.88, -5.28)
All non-White	19.6 (16.16, 23.33)	-4.35 (-8.32, -0.35)
Age group, years		
18–29	15.04 (10.92, 19.81)	-3.2 (-9.14, 2.23)
30–44	16.53 (13.84, 19.57)	-1.94 (-6, 2.74)
45–64	16.6 (13.99, 19.61)	-1.04 (-5.44, 3.89)
65–74	45.74 (40.72, 50.75)	-11.67 (-20.98, -2.49)
75 +	54.01 (46.12, 61.79)	-10.15 (-22.25, 0.43)

Abbreviations: CI, credible interval; PD, prevalence difference; Prev, prevalence.

^a^ Population adjusted seroprevalence in round 3 estimated using multilevel regression and poststratification (MRP); Seroprevalence and 95% CI are the mean and 2.5% and 97.5% quantiles of a posterior distribution respectively. Assay used detects antibodies from natural SARS-CoV-2 infection or from COVID-19 vaccination.

^b^ Population adjusted COVID-19 seroprevalence difference in round 3 between individuals in each race/ethnicity group and White individuals among race/ethnicity and between non-White individuals and White individuals within each age group. PDs estimated using MRP, and the PDs and 95% CIs are the mean and 2.5% and 97.5% quantiles of a posterior distribution, respectively.

### Mask-wearing and association between high-risk vs. low-risk behavior and seroprevalence

More than 99% of participants reported ever wearing a mask 99% reported wearing a mask during leisure and exercise activities, >91% reported wearing a mask at work, and >88% reported wearing a mask while shopping (S3 Table). After clustering participants into “low-risk” and “high-risk” groups according to self-reported mitigation behaviors, most participants were considered low-risk across the study rounds (70%, 82%, and 77%, respectively; S4 Fig). Behaviors with the largest differences in high- versus low-risk behavior were reporting “yes” to: left home for work, medical/healthcare, care of relative, or other; worked with potential COVID-19 contact; attended gathering; and traveled to county outside of residence within last two weeks (S4 Fig). Results from *χ*^2^ tests indicated that age, race/ethnicity, and education were all significantly associated (*P*<0.001) with mitigation behavior (S4 Table), but we did not observe evidence for an association between mitigation behavior and either seroprevalence or self-reported test positivity (Table 5).

**Table 5:** Association of high-risk vs. low-risk mitigation behavior with seroprevalence and self-reported test positivity and within each study round.

	MOA^a^	Round 1	Round 2	Round 3
SARS-CoV-2 antibody prevalence^b^	PD % (95% CI)	-0.21 (-1.4, 0.79)	0.02 (-1.59, 1.03)	-0.46 (-2.23, 1.00)
	PR (95% CI)	0.93 (0.34, 2.03)	1.4 (0.32, 3.93)	0.88 (0.45, 1.57)
Self-reported test positivity	PD % (95% CI)	0.53 (-0.73, 2.24)	0.11 (-2.12, 1.6)	-1.11 (-5.26, 2.18)
	PR (95% CI)	2.17 (0.54, 6.72)	1.56 (0.33, 4.65)	0.87 (0.41, 1.64)

Abbreviations: CI, credible interval; MOA, measure of association; PD, prevalence difference; PR, prevalence ratio.

^a^ Populated adjusted prevalence estimated using multilevel regression and poststratification models; point estimate is the mean of a posterior distribution of the parameter, 95% credible interval estimated from 2.5% and 97.5% quantiles of a posterior distribution.

^b^ Defined as having detectable SARS-CoV-2 antibodies within a study round.

## Discussion

In the current study, we investigated individual-level characteristics and behaviors that contributed to SARS-CoV-2 related outcomes, including seroprevalence and self-reported infection, in a large, population-based sample of over 5,500 participants from 12 East Bay (Northern California) cities. During three time periods from July 2020 to March 2021, we estimated the population-adjusted prevalence of SARS-CoV-2 outcomes across the study region and within strata of age, sex, race/ethnicity, ZIP code, and household size. We then characterized behaviors to mitigate transmission of SARS-CoV-2 and their association with related outcomes. Overall, prevalence of SARS-CoV-2 outcomes for natural infection were low which may be attributable to the high percentage of mask-wearing and other risk-mitigating behaviors among our participants. COVID-19 vaccination seroprevalence estimates in Round 3 were greater than 20%, with individuals identifying as African American or Black, American Indian or Alaskan Native, Asian, Hispanic, two or more races, or other having lower COVID-19 vaccination seroprevalence estimates than White individuals.

Despite the low overall SARS-CoV-2 seroprevalence and infection observed in our study, we observed evidence for differences in seroprevalence by ZIP code of residence, racial/ethnic identification, educational attainment, and household income. Further, ZIP codes with higher proportions of Spanish speakers had higher populated-adjusted seroprevalence estimates (S5 Fig). These differences persisted despite the high rates of mask wearing reported by our study sample, further adding to strong evidence that the risk of COVID-19 is distributed unequally and that structural inequities play an important role in COVID-19 risk [7,25–28]. Moreover, during Round 3 (February-March 2021), COVID-19 vaccines were widely available across the study region. We found that White individuals had higher prevalence of antibodies from COVID-19 vaccination in Round 3, than individuals identifying as African American or Black, American Indian or Alaskan Native, Asian, Hispanic, two or more races, or other, as reported elsewhere [3,29]. Notably, this difference was largest among those aged 65 or older. We believe this discrepancy is not explained by differences in vaccine hesitancy within racial and ethnic groups in our sample (S6 Table). In Round 3, among the n = 2,572 participants who self-reported not receiving at least one dose of a COVID-19 vaccine, 95.6% (n = 2,462) reported having plans to get vaccinated. This rate was greater than 91% across all racial and ethnic groups. Of the 110 individuals who did not report getting vaccinated, only 18 (16.4%) reported no plans to get vaccinated. These findings demonstrate that in the first few months of vaccine availability in the Bay Area, large disparities in vaccination rates by race/ethnicity were observed among older persons. Furthermore, White individuals, the group with the lowest prevalence of SARS-CoV-2 infection, were more likely to be vaccinated, underscoring the inequities that exist surrounding the coronavirus pandemic.

Another key finding was that almost all participants reported wearing masks. This contrasts with models of mask usage reported by the Institute for Health Metrics and Evaluation which reported mask usage ranging between 75–82% from December 2020 through March 2021 in California [30,31]. Mask wearing is one the most effective behaviors for controlling community spread of SARS-CoV-2 infection [32]. The high rate of mask usage by study participants may partially explain why we did not detect a differences between high-risk and low-risk mitigation behavior and SARS-CoV-2 prevalence, and partially explain why our estimates of SARS-CoV-2 seroprevalence and self-report test positivity were lower than public case reports.

A major strength of this study was the longitudinal design and collection of individual-level data, including biospecimens for antibody and virus testing, which is challenging but much needed in current studies of the pandemic. Comprehensive data on social distancing, self-quarantine, mask wearing, working from home, and other transmission mitigation efforts are also needed to inform current and future prevention strategies. At-home collection of biospecimens, including DBS for antibody testing, made regular testing without in-person interaction possible. This was a critical feature, particularly early in the pandemic, when recommendations were to travel only for essential purposes and to limit in-person interactions. At-home sample collection was used to obtain more than 30,000 biospecimens and is a feasible approach for large populations and geographic regions.

One limitation of this study was the under-representation of certain demographics in our sample. Supplementary mailings of recruitment postcards in Spanish were sent to residences in ZIP codes with high proportions of Spanish speaking households. We also placed recruitment flyers in local grocery stores and conducted outreach to community organizations, local government officials, and school districts in the study region. Despite these efforts, individuals identifying as African American or Black, American Indian or Alaskan Native, Asian, Hispanic, two or more races, or other, males, individuals from lower income households and with lower educational attainment, and individuals residing in lower socioeconomic ZIP codes were underrepresented in our sample. This was important given evidence that individuals who identify as Hispanic or Black, and other underrepresented groups, are at the highest risk for COVID-19 [28]. The low response rates of individuals from groups at higher COVID-19 infection likely contributed to an overall underestimation of SARS-CoV-2 seroprevalence in our study region. Evidence of the relationship between low-response rates and prevalence underestimation can be seen by comparing our seroprevalence estimates to COVID-19 case prevalence reported by Alameda County and Contra Costa County public health agencies [33]. In ZIP codes with low response rates the postcard invitation (e.g., 94601, 94603, 94621) we observed lower estimated prevalence in our study compared to public health agency case reports. The use of at-home testing kits may have excluded individuals from our sample who were either unable or unwilling to collect their own DBS, although we believe this limitation is offset by the insurmountable logistical challenges posed by in-person collection, especially when strict lockdowns were in place during the early phase of the study. Our testing algorithm for identifying individuals with antibodies from COVID-19 vaccination was unable to distinguish antibodies from vaccination versus natural infection in individuals who had both been previously infected and vaccinated. However, this should not have significantly affected our vaccination seroprevalence, as our natural infection seroprevalence estimates in Round 3 were low and only a subset of individuals with previous natural infection were also vaccinated. Another limitation is the possibility of self-selection bias, whereby individuals who joined our study were also more likely to be fervent adherers to COVID-19 public health measures than the general population. Additionally, a small proportion of individuals do not generate detectable antibodies after infection or vaccination for COVID-19. And lastly, there may be unmeasured confounding from variables not included in the analyses.

Our results underscore the substantial and persistent inequities that exist surrounding the coronavirus pandemic. Individuals identifying as African American or Black, American Indian or Alaskan Native, Asian, Hispanic, two or more races, or other, as well as those in lower-income households, and lower-educated individuals had the highest SARS-CoV-2 seroprevalence. We also observed large differences in COVID-19 vaccination seroprevalence between racial and ethnic groups. These disparities in COVID-19 infection and vaccination seroprevalence were observed despite low-response rates of individuals from groups at higher risk for COVID-19 infection in our sample and the near universal rates of mask wearing reported by participants. More work must be done to address these disparities and inequities, such as allocation of resources for high-risk communities and strategies to mitigate the structural barriers posed by social and structural determinants of health.

## Supporting information

S1 Fig Antibody testing algorithm for each study round. Antibodies against the spike (S) protein indicate prior or current SARS-CoV-2 infection or COVID-19 vaccination. Antibodies against the nucleocapsid (NC) protein indicate prior or current SARS-CoV-2 infection.
(PDF)

S2 Fig Population-adjusted covid probable prevalence by zip code (July 2020-April 2021). Base map and data from OpenStreetMap and OpenStreetMap Foundation (https://www.openstreetmap.org/copyright).
(PDF)

S3 Fig Populated-adjusted self-reported COVID-19 test positivity in each study round among demographic subgroups, A) sex, B) age, C) race/ethnicity, D) income, E) education, and F) household size. (PDF)

S4 Fig Proportion of participants stratified by high- and low-risk mitigation behaviors in each study round, A) overall, and B) according to specific behavior. (PDF)

S5 Fig Scatterplot of proportion of households who speak Spanish at home reported by the American Com-munity Survey (x-axis) and cumulative population-adjusted SARS-CoV-2 seroprevalence (y-axis) within ZIP codes. (PDF)

S6 Fig Comparison of SARS-CoV-2 seroprevalence estimated in study to cumulative case prevalence reported by local public health agencies (July 2020-April 2021): A) Oakland/Piedmont, B) Albany, Berkeley, and Emeryville, and D) El Cerrito, Richmond, and San Pablo. The household response rate (RR) to the study invitation within each ZIP code is included in the upper left of each panel.
(PDF)

S1 Table ZIP code of residence of participants at each round of the study compared to study region population. (PDF)

S2 Table Distribution of COVID-19 probable case definition variables. (PDF)

S3 Table Self-reported mask wearing behavior during each study round. (PDF)

S4 Table Characteristics of study participants stratified by high-risk and low-risk mitigation behavior. (PDF)

S5 Table Antibody assays used to detect antibodies against SARS-CoV-2 spike and nucleocapsid proteins. (PDF)

S6 Table Self-reported vaccine plans among participants who did not self-report receiving a vaccination in Round 3 stratified by race and ethnicity. (PDF)

S1 File Methods used for SARS-CoV-2 viral and antibody detection, and participant recruitment. (PDF)

S2 File Study questionnaires used for each data collection round. (PDF)

S3 File Details on data collection and SARS-CoV-2 outcome definitions. (PDF)

S4 File Statistical methods used for population adjusted prevalence analyses. (PDF)

S5 File Behaviors related to virus containment and mitigation. (PDF)

S6 File Population adjusted prevalence estimates of COVID-19 outcomes and 95% credible intervals across demographic and regional strata. See tables for region and stratum population-adjusted estimates for COVID-19 outcomes and mitigation analyses.
(XLSX)
