# Supplementary material for: Health inequities in SARS-CoV-2 infection, seroprevalence, and COVID-19 vaccination: Results from the East Bay COVID-19 study
Source: PLOS Glob Public Health. 2022 Aug 15;2(8):e0000647. doi: 10.1371/journal.pgph.0000647 (PMC10022102; doi:10.1371/journal.pgph.0000647)
Supplement: S1 Fig — Antibodies against the spike (S) protein indicate prior or current SARS-CoV-2 infection or COVID-19 vaccination. Antibodies against the nucleocapsid (NC) protein indicate prior or current SARS-CoV-2 infection. (PDF) [file pgph.0000647.s001.pdf]

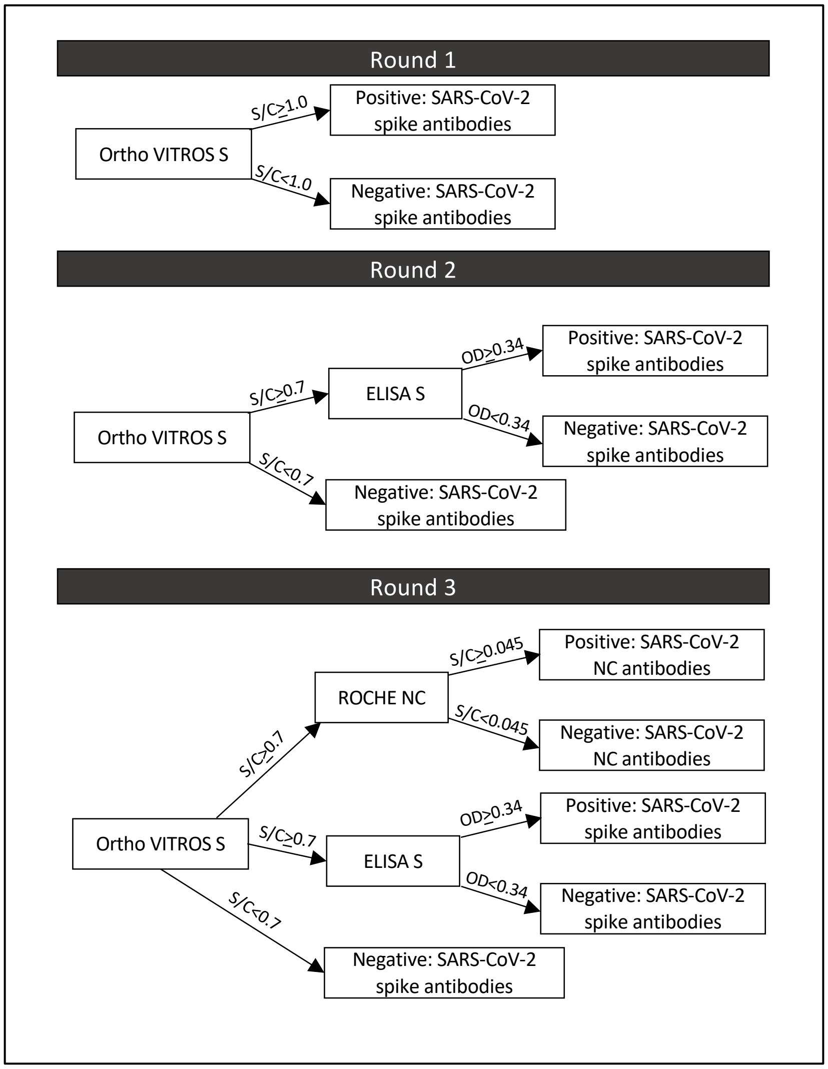

Fig S1. Antibody testing algorithm for each study round. Antibodies against the spike (S) protein indicate prior or current SARS-CoV-2 infection or COVID-19 vaccination. Antibodies against the nucleocapsid (NC) protein indicate prior or current SARS-CoV-2 infection.
