# Supplementary material for: Health inequities in SARS-CoV-2 infection, seroprevalence, and COVID-19 vaccination: Results from the East Bay COVID-19 study
Source: PLOS Glob Public Health. 2022 Aug 15;2(8):e0000647. doi: 10.1371/journal.pgph.0000647 (PMC10022102; doi:10.1371/journal.pgph.0000647)
Supplement: S2 Fig — Base map and data from OpenStreetMap and OpenStreetMap Foundation (https://www.openstreetmap.org/copyright). (PDF) [file pgph.0000647.s002.pdf]

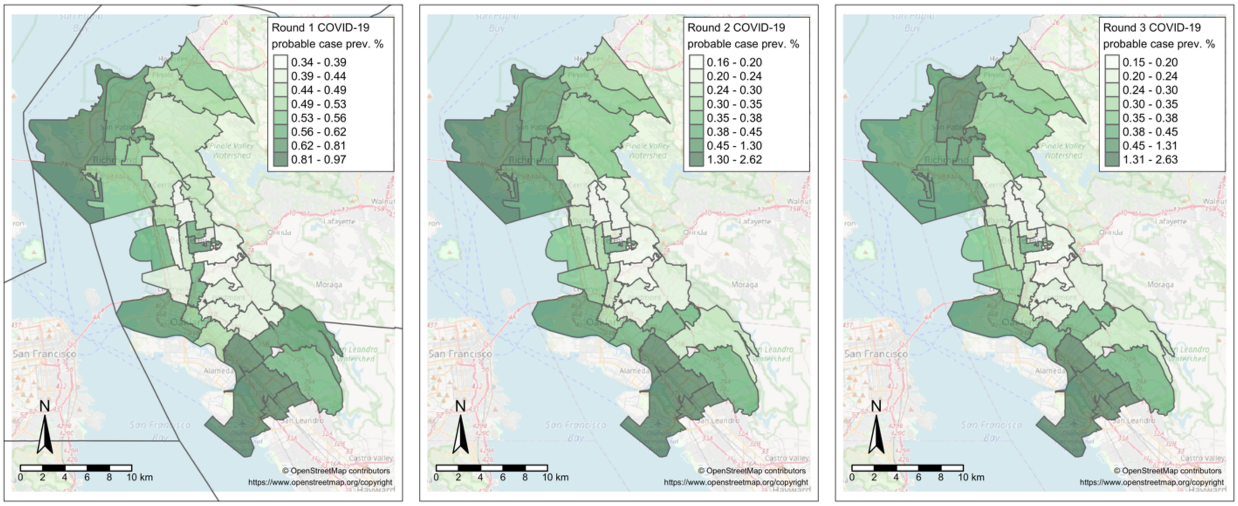

Fig S2. Population-adjusted covid probable prevalence by zip code (July 2020-April 2021). Base map and data from OpenStreetMap and OpenStreetMap Foundation.
