# Supplementary material for: Health inequities in SARS-CoV-2 infection, seroprevalence, and COVID-19 vaccination: Results from the East Bay COVID-19 study
Source: PLOS Glob Public Health. 2022 Aug 15;2(8):e0000647. doi: 10.1371/journal.pgph.0000647 (PMC10022102; doi:10.1371/journal.pgph.0000647)
Supplement: S3 Fig — (PDF) [file pgph.0000647.s003.pdf]

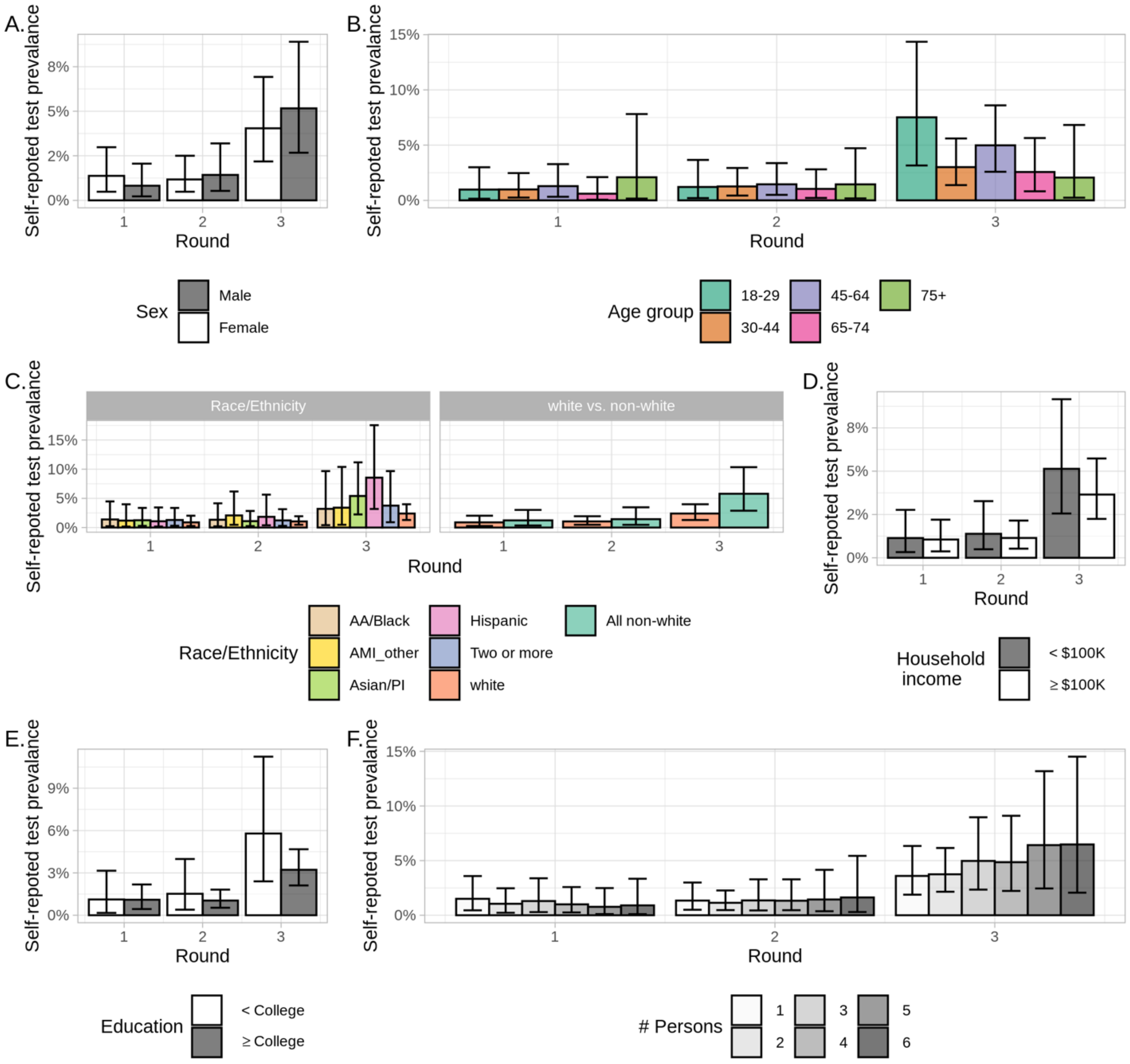

Fig S3. Populated-adjusted self-reported COVID-19 test positivity in each study round among demographic subgroups, A) sex, B) age, C) race/ethnicity, D) income, E) education, and F) household size.
