# Supplementary material for: Health inequities in SARS-CoV-2 infection, seroprevalence, and COVID-19 vaccination: Results from the East Bay COVID-19 study
Source: PLOS Glob Public Health. 2022 Aug 15;2(8):e0000647. doi: 10.1371/journal.pgph.0000647 (PMC10022102; doi:10.1371/journal.pgph.0000647)
Supplement: S4 Fig — (PDF) [file pgph.0000647.s004.pdf]

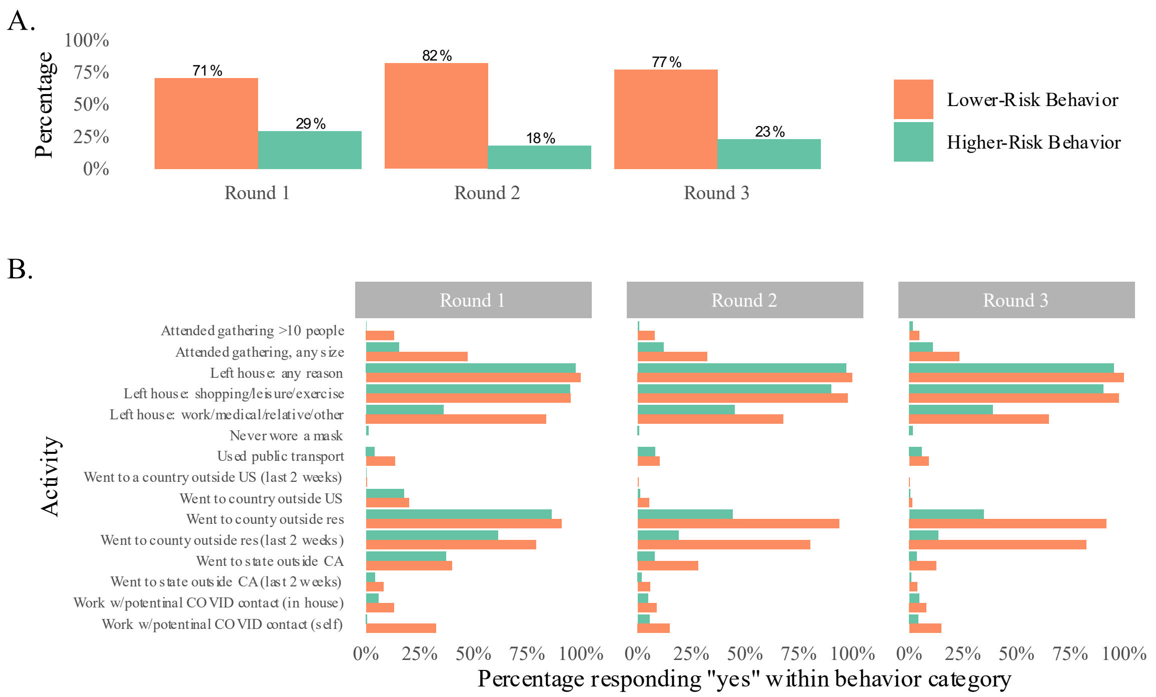

Fig S4. Proportion of participants stratified by high- and low-risk mitigation behaviors in each study round, A) overall, and B) according to specific behavior.
