# Supplementary material for: Health inequities in SARS-CoV-2 infection, seroprevalence, and COVID-19 vaccination: Results from the East Bay COVID-19 study
Source: PLOS Glob Public Health. 2022 Aug 15;2(8):e0000647. doi: 10.1371/journal.pgph.0000647 (PMC10022102; doi:10.1371/journal.pgph.0000647)
Supplement: S5 Fig — (PDF) [file pgph.0000647.s005.pdf]

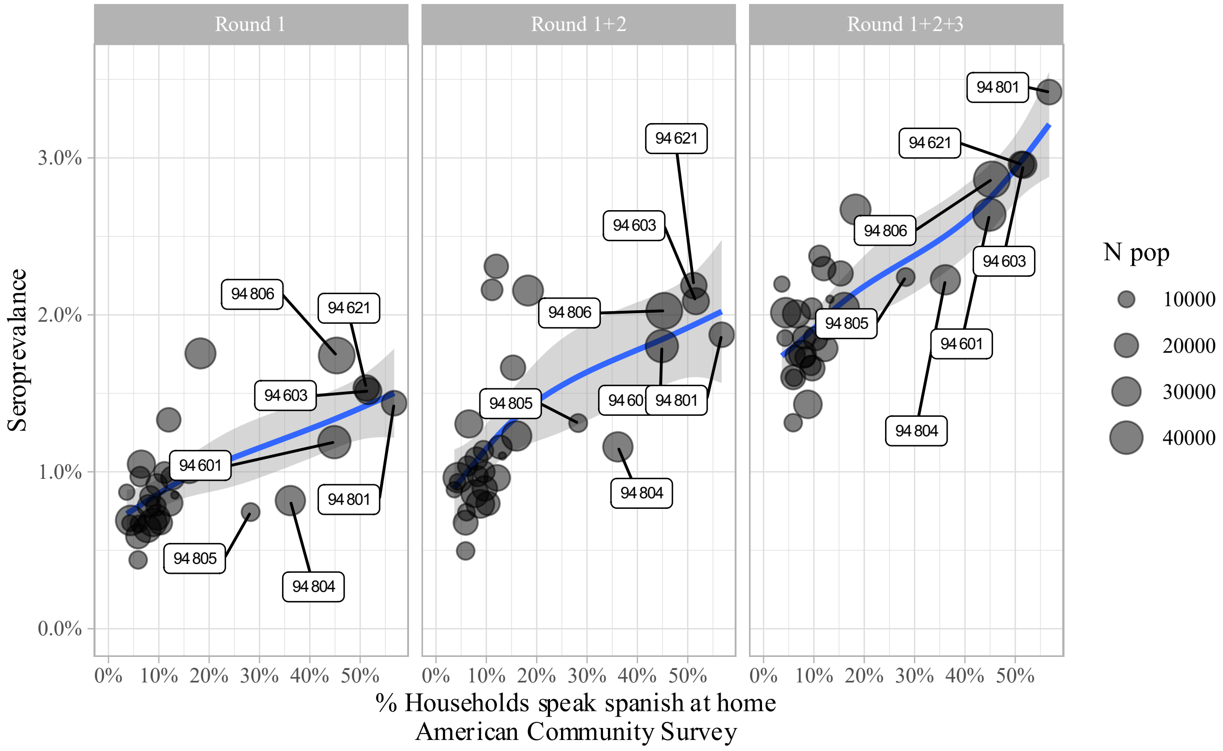

Fig S5. Scatterplot of proportion of households who speak Spanish at home reported by the American Community Survey (x-axis) and cumulative population-adjusted SARS-CoV-2 sero-prevalence (y-axis) within ZIP codes.
