# Supplementary material for: Health inequities in SARS-CoV-2 infection, seroprevalence, and COVID-19 vaccination: Results from the East Bay COVID-19 study
Source: PLOS Glob Public Health. 2022 Aug 15;2(8):e0000647. doi: 10.1371/journal.pgph.0000647 (PMC10022102; doi:10.1371/journal.pgph.0000647)
Supplement: S6 Fig — Comparison of SARS-CoV-2 seroprevalence estimated in study to cumulative case prevalence reported by local public health agencies (July 2020-April 2021): A) Oakland/Piedmont, B) Albany, Berkeley, and Emeryville, and D) El Cerrito, Richmond, and San Pablo. The household response rate (RR) to the study invitation within each ZIP code is included in the upper left of each panel. (PDF) [file pgph.0000647.s006.pdf]

A.

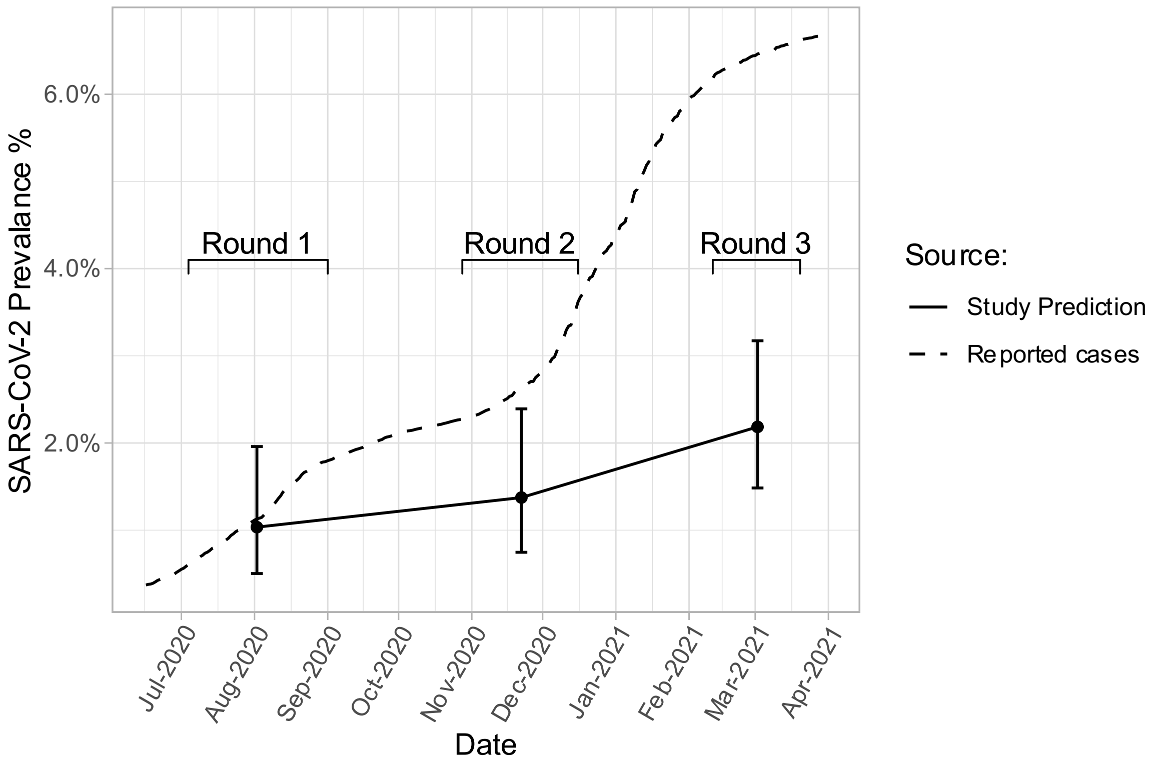

Fig S6. Comparison of SARS-CoV-2 seroprevalence estimated in study to cumulative case prevalence reported by local public health agencies (July 2020-April 2021): A) Oakland/Piedmont, B) Albany, Berkeley, and Emeryville, and D) El Cerrito, Richmond, and San Pablo. The household response rate (RR) to the study invitation within each ZIP code is included in the upper left of each panel.

B.

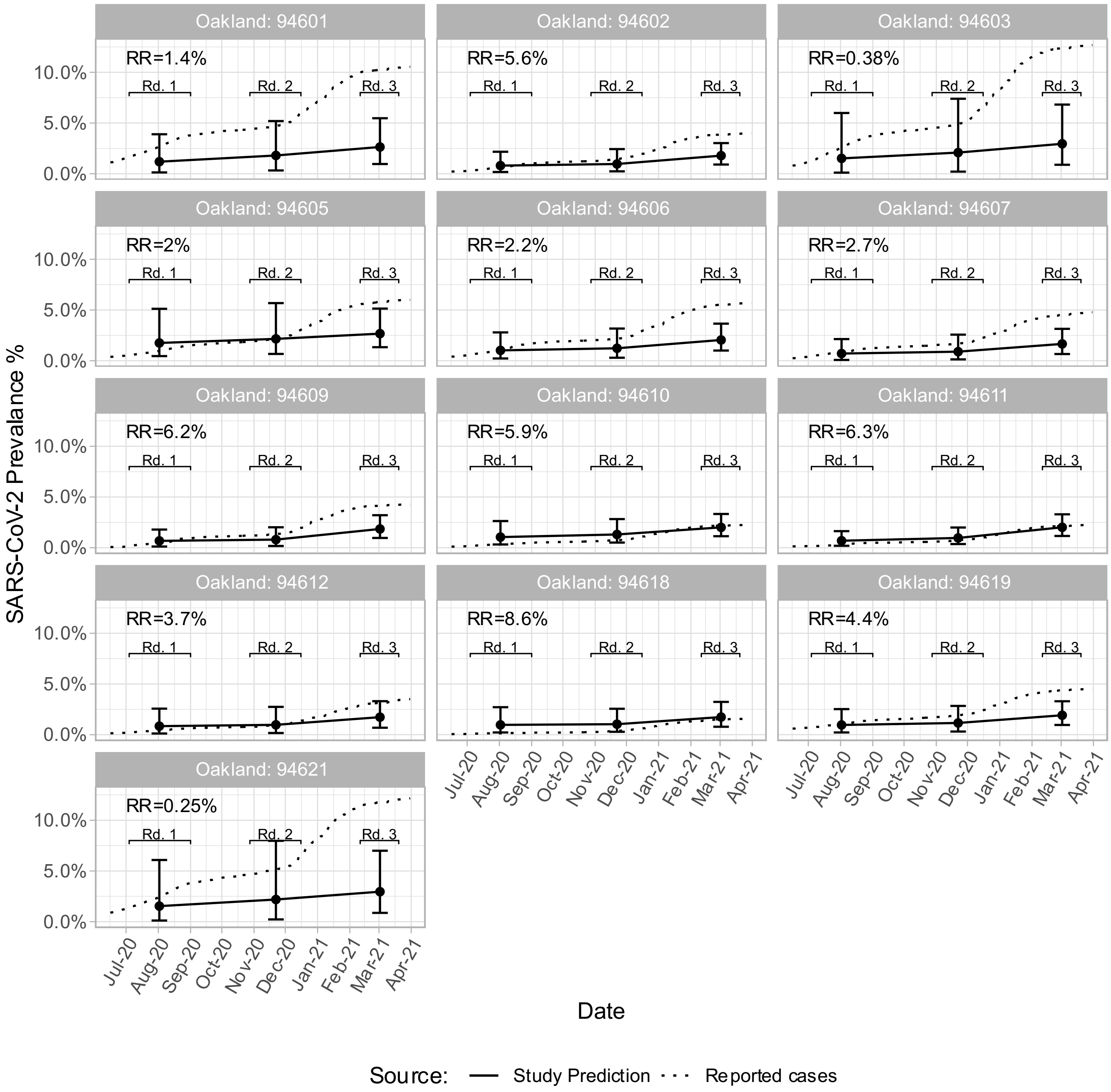
Oakland: 94601
RR=1.4%
Oakland: 94602
RR=5.6%
Oakland: 94603
RR=0.38%
Oakland: 94605
RR=2%
Oakland: 94606
RR=2.2%
Oakland: 94607
RR=2.7%
Oakland: 94609
RR=6.2%
Oakland: 94610
RR=5.9%
Oakland: 94611
RR=6.3%
Oakland: 94612
RR=3.7%
Oakland: 94618
RR=8.6%
Oakland: 94619
RR=4.4%
Oakland: 94621
RR=0.25%
Rd. 1
Rd. 2
Rd. 3
10.0%
5.0%
0.0%
SARS-CoV-2 Prevalance %
Jul-20
Aug-20
Sep-20
Oct-20
Nov-20
Dec-20
Jan-21
Feb-21
Mar-21
Apr-21
Date
Source:
Study Prediction
Reported cases

C.

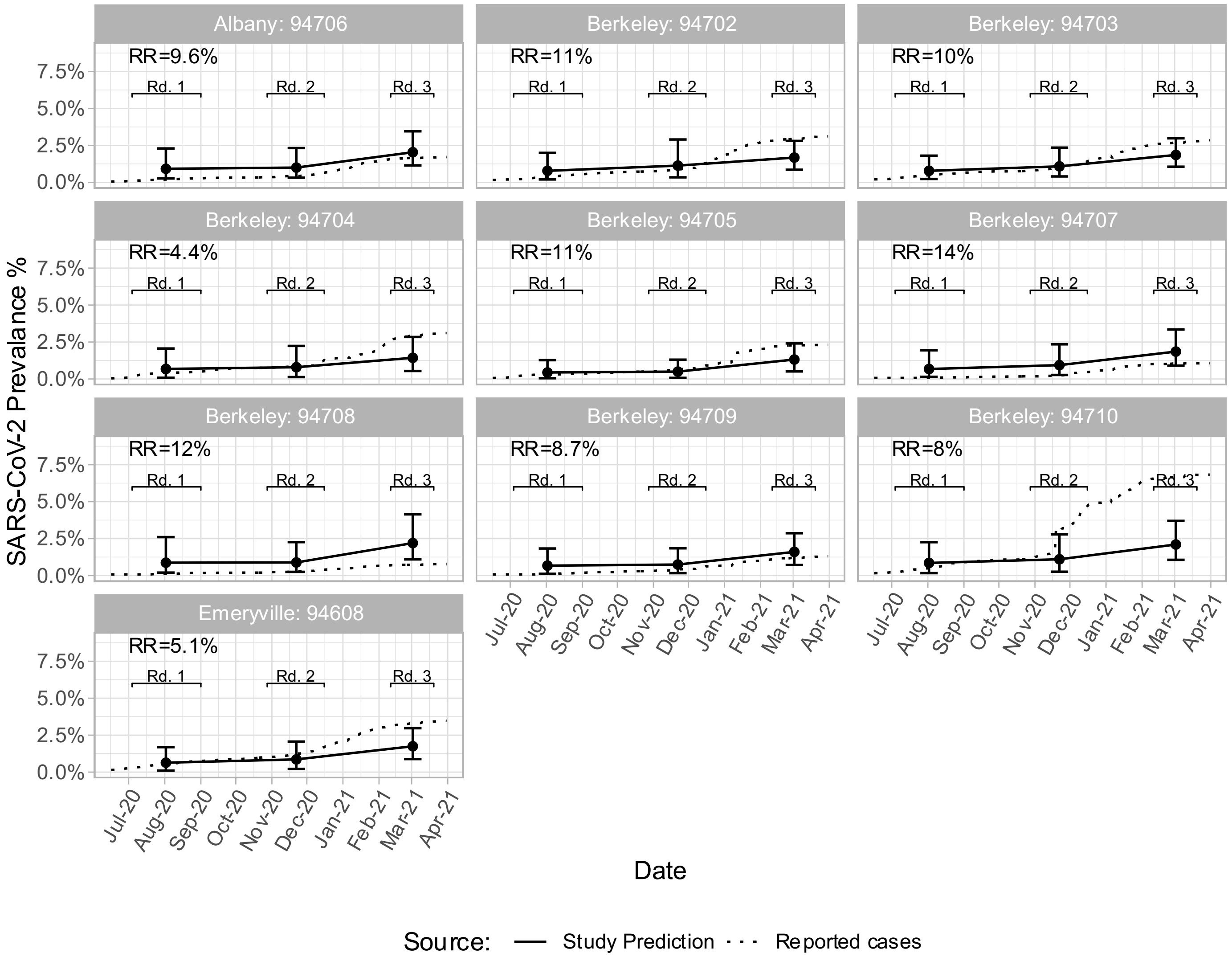

Albany: 94706
RR=9.6%
Berkeley: 94702
RR=11%
Berkeley: 94703
RR=10%
Berkeley: 94704
RR=4.4%
Berkeley: 94705
RR=11%
Berkeley: 94707
RR=14%
Berkeley: 94708
RR=12%
Berkeley: 94709
RR=8.7%
Berkeley: 94710
RR=8%
Emeryville: 94608
RR=5.1%
Rd. 1
Rd. 2
Rd. 3
7.5%
5.0%
2.5%
0.0%
SARS-CoV-2 Prevalance %
Jul-20
Aug-20
Sep-20
Oct-20
Nov-20
Dec-20
Jan-21
Feb-21
Mar-21
Apr-21
Date
Source:
Study Prediction
Reported cases

D.

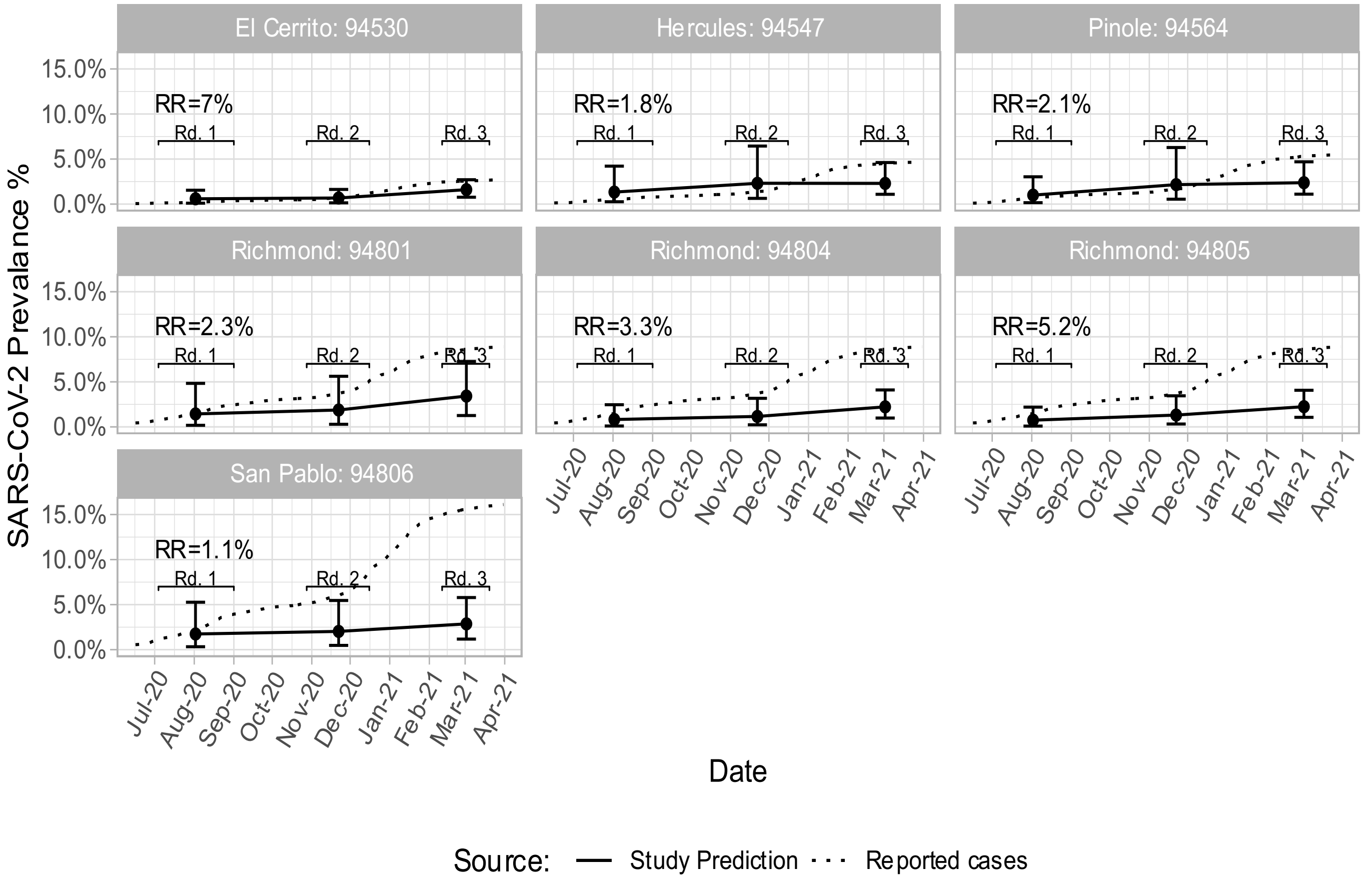

El Cerrito: 94530
Hercules: 94547
Pinole: 94564
Richmond: 94801
Richmond: 94804
Richmond: 94805
San Pablo: 94806
RR=7%
RR=1.8%
RR=2.1%
RR=2.3%
RR=3.3%
RR=5.2%
RR=1.1%
Rd. 1
Rd. 2
Rd. 3
SARS-CoV-2 Prevalance %
15.0%
10.0%
5.0%
0.0%
Jul-20
Aug-20
Sep-20
Oct-20
Nov-20
Dec-20
Jan-21
Feb-21
Mar-21
Apr-21
Date
Source:
Study Prediction
Reported cases
