# Supplementary material for: Health inequities in SARS-CoV-2 infection, seroprevalence, and COVID-19 vaccination: Results from the East Bay COVID-19 study
Source: PLOS Glob Public Health. 2022 Aug 15;2(8):e0000647. doi: 10.1371/journal.pgph.0000647 (PMC10022102; doi:10.1371/journal.pgph.0000647)
Supplement: S1 Table — (PDF) [file pgph.0000647.s007.pdf]

Table S-1. ZIP code of residence of participants at each round of the study compared to study region population.

	PS Strata† %	Round 1 no. (%)	Round 2 no. (%)	Round 3 no. (%)
Invited to round, N		7166	6242	5506
Participated in study round, N		5501 (76.8)	5603 (89.8)	4806 (87.3)
City				
Albany	2.3	245 (4.5)	258 (4.6)	229 (4.8)
Berkeley	16.1	1446 (26.3)	1535 (27.4)	1314 (27.3)
El Cerrito	3.1	226 (4.1)	257 (4.6)	226 (4.7)
El Sobrante	3.2	113 (2.1)	116 (2.1)	107 (2.2)
Emeryville	4.0	309 (5.6)	289 (5.2)	236 (4.9)
Hercules	3.0	66 (1.2)	69 (1.2)	58 (1.2)
Oakland‡	48.8	2417 (43.9)	2417 (43.1)	2061 (42.9)
Pinole	2.4	44 (0.8)	48 (0.9)	38 (0.8)
Richmond	9.8	502 (9.1)	496 (8.9)	440 (9.2)
San Pablo	7.4	133 (2.4)	118 (2.1)	97 (2)

*PS, Poststratification; USD, United States Dollar.

†Population percentages from synthetic poststratification tables generated from American Community Survey (ACS) and Public Use Microdata Sample data. Each cell contains the percentage of population age >18. Gender is not available in ACS data.

‡Piedmont included in Oakland.
