# Supplementary material for: Health inequities in SARS-CoV-2 infection, seroprevalence, and COVID-19 vaccination: Results from the East Bay COVID-19 study
Source: PLOS Glob Public Health. 2022 Aug 15;2(8):e0000647. doi: 10.1371/journal.pgph.0000647 (PMC10022102; doi:10.1371/journal.pgph.0000647)
Supplement: S2 Table — (PDF) [file pgph.0000647.s008.pdf]

Table S-2. Distribution of COVID-19 probable case definition variables.

		Round 1	Round 2	Round 3
N		5501	5603	4806
COVID-like symptoms[a]				
	No	4574 (83.1)	5092 (90.9)	4143 (86.2)
	Yes	898 (16.3)	339 (6.1)	571 (11.9)
	Not reported	29 (0.5)	172 (3.1)	92 (1.9)
COVID close contact[a]				
	No	4720 (85.8)	4873 (87.0)	4370 (90.9)
	Yes	101 (1.8)	75 (1.3)	57 (1.2)
	Not reported	680 (12.4)	655 (11.7)	379 (7.9)
COVID probable case[a]				
	No	4800 (87.3)	4938 (88.1)	4419 (91.9)
	Yes	21 (0.4)	10 (0.2)	8 (0.2)
	Not reported	680 (12.4)	655 (11.7)	379 (7.9)

Abbreviations: DBS, dried blood spot; QNS, Quantity not sufficient

[a]COVID-like symptoms, COVID close-contact, and COVID probable case were defined by Council and State Territorial epidemiologists.[4]
