# Supplementary material for: Health inequities in SARS-CoV-2 infection, seroprevalence, and COVID-19 vaccination: Results from the East Bay COVID-19 study
Source: PLOS Glob Public Health. 2022 Aug 15;2(8):e0000647. doi: 10.1371/journal.pgph.0000647 (PMC10022102; doi:10.1371/journal.pgph.0000647)
Supplement: S3 Table — (PDF) [file pgph.0000647.s009.pdf]

**Table S-3.** Self-reported mask wearing behavior during each study round

| | Round 1 | Round 2 | Round 3 |
|---|---|---|---|
| Mask wearing behavior n (%) | | | |
| Ever wear a mask=Yes | 5432 (99.3) | 5420 (99.8) | 4694 (99.6) |
| Wear during leisure/exercise=Yes | 4433 (98.9) | 4619 (99.1) | 3951 (99.1) |
| Wear at work=Yes | 1259 (91.9) | 1488 (93.2) | 1251 (95.0) |
| Wear while shopping=Yes | 3824 (88.0) | 4041 (92.2) | 3621 (94.8) |
| Wear while home=Yes | 399 (7.3) | 556 (10.2) | 475 (10.1) |
