# Supplementary material for: Health inequities in SARS-CoV-2 infection, seroprevalence, and COVID-19 vaccination: Results from the East Bay COVID-19 study
Source: PLOS Glob Public Health. 2022 Aug 15;2(8):e0000647. doi: 10.1371/journal.pgph.0000647 (PMC10022102; doi:10.1371/journal.pgph.0000647)
Supplement: S4 Table — (PDF) [file pgph.0000647.s010.pdf]

Table S-4. Characteristics of study participants stratified by high-risk and low-risk mitigation behavior.

	Round 1			Round 2			Round 3		
	High Risk	Low Risk		High Risk	Low Risk		High Risk	Low Risk	
N	1599	3871	*P*	977	4452	*P*	1084	3630	*P*
Sex			0.999			0.001			0.089
Female	1018 (63.7)	2465 (63.7)		573 (58.7)	2874 (64.6)		718 (66.2)	2299 (63.4)	
Male	581 (36.3)	1404 (36.3)		403 (41.3)	1577 (35.4)		366 (33.8)	1330 (36.6)	
Age, years			<0.001			<0.001			<0.001
18 – 29	170 (10.6)	296 (7.6)		80 (8.2)	281 (6.3)		67 (6.2)	213 (5.9)	
30 - 44	620 (38.8)	1138 (29.4)		360 (36.9)	1231 (27.7)		245 (22.6)	1049 (28.9)	
45 – 64	621 (38.9)	1423 (36.8)		379 (38.8)	1703 (38.3)		396 (36.6)	1464 (40.4)	
65 – 74	161 (10.1)	764 (19.7)		131 (13.4)	919 (20.7)		254 (23.5)	713 (19.7)	
75 +	26 (1.6)	249 (6.4)		26 (2.7)	313 (7.0)		120 (11.1)	188 (5.2)	
Race			<0.001			<0.001			<0.001
African American/Black	75 (4.7)	192 (5.0)		21 (2.2)	148 (3.3)		51 (4.7)	87 (2.4)	
Native American/ Alaskan Native or Other	32 (2.0)	91 (2.4)		16 (1.6)	89 (2.0)		22 (2.0)	58 (1.6)	
Asian/Pacific Islander	244 (15.3)	609 (15.7)		160 (16.4)	580 (13.0)		146 (13.5)	513 (14.1)	
Hispanic	312 (19.5)	540 (14.0)		122 (12.5)	480 (10.8)		129 (11.9)	377 (10.4)	
Two or more races	155 (9.7)	345 (8.9)		92 (9.4)	288 (6.5)		65 (6.0)	263 (7.3)	
White	781 (48.8)	2092 (54.1)		565 (57.9)	2862 (64.4)		668 (61.8)	2329 (64.2)	
Education			<0.001			0.251			<0.001
College degree	1392 (87.1)	3519 (91.1)		901 (92.4)	4058 (91.2)		956 (88.3)	3368 (92.8)	
No college degree	206 (12.9)	345 (8.9)		74 (7.6)	391 (8.8)		127 (11.7)	260 (7.2)	
