# Supplementary material for: Health inequities in SARS-CoV-2 infection, seroprevalence, and COVID-19 vaccination: Results from the East Bay COVID-19 study
Source: PLOS Glob Public Health. 2022 Aug 15;2(8):e0000647. doi: 10.1371/journal.pgph.0000647 (PMC10022102; doi:10.1371/journal.pgph.0000647)
Supplement: S5 Table — (PDF) [file pgph.0000647.s011.pdf]

Table S-5. Antibody assays used to detect antibodies against SARS-CoV-2 spike and nucleocapsid proteins.

	**Round 1**	**Round 2**	**Round 3**	**Round 3**
Antibody Target	Spike	Spike	Spike	Nucleocapsid
Assay #1				
Name	VITROS Anti-SARS-CoV-2 Total Ig	VITROS Anti-SARS-CoV-2 Total Ig	VITROS Anti-SARS-CoV-2 Total Ig	VITROS Anti-SARS-CoV-2 Total Ig
DBS tested	All	All	All	All
Criteria	S/C ≥ 1: Reactive<br>S/C <1: Non-reactive	S/C ≥ 0.7: ELISA<br>S/C < 0.7: Non-reactive	S/C ≥ 0.7: ELISA<br>S/C < 0.7: Non-reactive	S/C ≥ 0.7: Roche<br>S/C < 0.7: Non-reactive
Se/Sp (95% CI)	Se: 80.6 (64.0 - 91.8)<br>Sp: 1 (63.1 - 1)	Se: 88.9 (73.9 - 96.9)<br>Sp: 1 (63.1 - 1)	Se: 88.9 (73.9 - 96.9)<br>Sp: 1 (63.1 - 1)	Se: 88.9 (73.9 - 96.9)<br>Sp: 1 (63.1 - 1)
Assay #2				
Name		ELISA IgG	ELISA IgG	Roche NC Total Ig
DBS tested		Ortho S/C ≥ 0.7	Ortho S/C ≥ 0.7	Ortho S/C ≥ 0.7
Criteria		OD ≥ 0.34: Reactive<br>OD < 0.34: Non-reactive	OD ≥ 0.34: Reactive<br>OD < 0.34: Non-reactive	S/C ≥ 0.0465: Reactive<br>S/C < 0.0465 Non-reactive
Se/Sp (95% CI)		Se: 0.972 (88.7 - 99.9)<br>Sp: 1 (87.7 - 1)	Se: 0.972 (88.7 - 99.9)<br>Sp: 1 (87.7 - 1)	Se: 86.7 (69.3 - 96.2)<br>Sp: 97.9 (94.8 - 99.4)

Se, sensitivity; Sp, specificity. Data on sensitivity and specificity from Wong et al.[18]
