# Supplementary material for: Health inequities in SARS-CoV-2 infection, seroprevalence, and COVID-19 vaccination: Results from the East Bay COVID-19 study
Source: PLOS Glob Public Health. 2022 Aug 15;2(8):e0000647. doi: 10.1371/journal.pgph.0000647 (PMC10022102; doi:10.1371/journal.pgph.0000647)
Supplement: S6 Table — (PDF) [file pgph.0000647.s012.pdf]

Table S-6. Self-reported vaccine plans among participants who did not self-report receiving a vaccination in Round 3 stratified by race and ethnicity.

		Self-report race and ethnicity					
	All	African American or Black	AMI or Other	Asian or Pacific Islander	Hispanic	Two or more races	White
n	2572	68	50	421	321	240	1470
Planning to get vaccinated, n (%)	2462 (95.6)	62 (91.2)	46 (92.0)	406 (96.4)	299 (93.1)	229 (95.4)	1418 (96.4)
Not planning to get vaccinated, n (%)	18 (0.7)	3 (4.4)	0 (0.0)	4 (1.0)	3 (0.9)	2 (0.8)	6 (0.4)
Unsure of plans, n (%)	63 (2.6)	3 (4.4)	3 (6.0)	6 (1.4)	17 (5.3)	8 (3.3)	26 (1.8)
Decline to answer, n (%)	29 (1.1)	0 (0.0)	1 (2.0)	5 (1.2)	2 (0.6)	1 (0.4)	20 (1.4)
