# Supplementary material for: Health inequities in SARS-CoV-2 infection, seroprevalence, and COVID-19 vaccination: Results from the East Bay COVID-19 study
Source: PLOS Glob Public Health. 2022 Aug 15;2(8):e0000647. doi: 10.1371/journal.pgph.0000647 (PMC10022102; doi:10.1371/journal.pgph.0000647)
Supplement: S1 File — (PDF) [file pgph.0000647.s013.pdf]

S1 File. Methods used for SARS-CoV-2 viral and antibody detection, and participant recruitment.

1 SARS-CoV-2 viral detection

Nucleic acid extraction to obtain (potential) viral RNA from study participants was performed in collaboration with the University of Minnesota Genomics Center. Briefly, nasal/nares swab samples provided by study participants in collection tubes with a stabilizing solution were first heat-inactivated and transferred to a 96-well plate for further processing to obtain RNA. Solid Phase Reversible Immobilization (SPRI) was used for nucleic acid extraction as previously described.[1] Briefly, Anterior nares (AN) swab samples in 3 mL DNA/RNA Shield (Zymo) were first heat-inactivated at 56°C for 20 minutes. 200 µL of the resulting solution was transferred to a 96-well deep well plate containing 150 uL of Lysis Buffer (4.67M GuHCl, 5.8 mM TCEP, 23.3 mM EDTA, 23.3 mM Tris, pH 7.0, 0.23 % (v/v) Igepal CA-630, 100 units/mL Proteinase K) and pipette-mixed 10 times. The plate was sealed and again incubated at 56°C for 20 minutes.

Following lysis/digestion, 350 µL of a SPRI bead solution (1:50 dilution of 3X washed Cytiva Sera-Mag SpeedBeads™ 65152105050250, 18% (w/v) PEG-8000, 1M NaCl, 10 mM Tris-HCl, 1 mM EDTA) was added to the lysate and pipette-mixed 10 times, incubated at room temperature for 10 minutes, and pipette-mixed an additional 10 times. Following incubation and mixing, the plate was placed on a magnet and the magnetic beads were allowed to pellet. After, the supernatant was removed, and the beads were washed twice with 800 µL freshly prepared 80% ethanol. Beads were allowed to dry off-magnet for 10 minutes at room temperature, and then were

resuspended with 50 µL nuclease-free H20. After magnetizing again, the clear supernatant was removed to a fresh plate, and this solution was used as input for RT-qPCR.

This RNA was used as input for reverse transcription quantitative real-time polymerase chain reaction (RT-qPCR). Three separate RT-qPCR reactions were set up for each study participant sample in a 384-well plate, and PCR was performed using CDC-specified primers and Taqman probes for viral N1 and N2 genes, and human RNase P (https://www.cdc.gov/coronavirus/2019-ncov/downloads/rt-pcr-panel-primer-probes.pdf) as described.[2] Following PCR thermocycling steps, the amplification results for each run were manually inspected, and results were interpreted to determine whether there was evidence of coronavirus infection for each participant (amplification cycle threshold for N1 or N2 gene $\leq$ 40).

Lysis Buffer

- 4.67M GuHCl
- 5.8 mM TCEP
- 23.3 mM EDTA
- 23.3 mM Tris, pH 7.0
- 0.23 % (v/v) Igepal CA-630
- 100 units/mL Proteinase K

Beads

- 1:50 dilution of 3X washed Cytiva Sera-Mag SpeedBeads™
- 18% (w/v) PEG-8000

- 1M NaCl
- 10 mM Tris-HCl
- 1 mM EDTA

2 SARS-CoV-2 antibody detection

Antibody testing was performed using dried blood spots (DBS) provided by study participants.

2.1 Quality Control of Dried Blood Spots

Participant’s DBS sample cards (containing six discs) were assessed for quality. We evaluated each DBS disc on a scale from 0-3, with 0 representing a blank DBS, 1 representing an incompletely filled DBS on one side, 2 representing a DBS card saturated fully on one side, and 3 representing a DBS fully saturated on both sides. Participants that provided at least two DBS discs with a cumulative score of two or higher were processed in our serological assays. Samples that did not meet this criterion were labelled as “Quantity Not Sufficient” (QNS).

2.2 Reconstitution of Blood Spots

Two DBSs were cut from disks and placed in 2mL screwcap tubes. 500uL of elution buffer was added into tube and vortexed for 20 seconds. If spots were limited, one spot was eluted in 250uL of elution buffer instead. DBS was incubated in elution buffer overnight at 4°C, then spun down at 10,600 g for 10 min at 4°C. DBS eluate was then transferred to a fresh tube and stored at 4°C until analysis.

2.3 Antibody testing

For each study round, two high quality DBS discs from each participant were rehydrated by soaking them in a reconstitution solution and were processed for testing. Over the course of the study, three assays were used: Ortho VITROS Anti-SARS-CoV-2 Total Ig test (ORTHO), anti-Spike IgG ELISA Assay (ELISA), and Roche NC Total Ig (ROCHE).

2.3.1 Ortho VITROS Anti-SARS-CoV-2 Total Ig test

DBS eluates were tested on the Ortho VITROS Anti-SARS-CoV-2 Total test according to the manufacturer's instructions described briefly. The Ortho VITROS Anti-SARS-CoV-2 total (CoV2T, Ortho-Clinical Diagnostics, Inc) was used to detect total (IgG, IgM, and IgA) antibodies to SARS-CoV-2 Spike S1 protein. Briefly, DBS eluates or plasma samples were loaded on Ortho VITROS XT-7200 or 3600 instruments (Ortho-Clinical Diagnostics, Inc) and programmed for the CoV2T test following the manufacturer's instructions. The S1 antigens coated on the assay wells bind S1 antibodies from human serum which, in turn, bind to a secondary HRP-labeled S1 antigen in the conjugate reagent forming a sandwich. The addition of signal reagent containing luminol generates a chemiluminescence reaction that is measured by the system and quantified as the ratio of the signal relative to the cut-off value generated during calibration. A S/CO $\geq$1 was considered positive.

2.3.2 *anti-Spike IgG ELISA Assay*

DBS eluates and plasma were evaluated for the presence of IgG against SARS-CoV-2 Spike (S) and using an in-house direct ELISA as previously described.[3] Briefly, SARS-CoV-2 antigens were coated in 96-well Nunc Maxisorp ELISA plates (ThermoFisher) overnight at 4°C. Plates were then blocked in 2.5% non-fat dry milk in PBS for 2h at 37°C. Plates were then washed 3x with PBS and 100uL of DBS eluate in 1% non-fat dry milk and incubated at 37°C for 1h. Plates were then washed 5 times with 0.05% PBS-Tween-20 and 100uL of goat-α-human IgG HRP secondary antibody (Fisher) diluted in 1% non-fat dry milk was added. After incubating for 1h at 37°C, plates were washed 5 times in 0.05% PBS-Tween-20 and once in PBS. Plate was developed with TMB (3,3',5,5'-Tetramethylbenzidine) for exactly 5 minutes and reaction was stopped with 2M H_2SO_4. Plates were read on a plate reader at 490nm.

2.3.3 *Roche NC Total Ig*

The Roche Elecsys Anti-SARS-CoV-2 immunoassay (Roche NC) was run on the Cobas e441 analyzer (Roche Diagnostics) to detect antibodies against the SARS-CoV-2 nucleocapsid protein. DBS eluates were initially incubated with biotinylated and ruthenium-labeled SARS-CoV-2 recombinant nucleocapsid antigens and any antibody present in the solution is sandwiched between the two. Subsequently, streptavidin-coated microparticles are added to the mixture to bind the biotin. The magnetic particles drive the complexes to the electrode, where a chemiluminescent signal is emitted and measured as the ratio between the signal and the cut-off obtained during calibration. A S/CO ≥1 in plasma was considered positive while a S/CO ≥ 0.045 in DBS was considered positive.

3 Recruitment and participants

Recruitment and selection of study participants was completed using a screening phase followed by a longitudinal study phase with three timepoints or “rounds”. In the screening phase, all residential addresses within the East Bay cities and communities of Albany, Berkeley, El Cerrito, El Sobrante, Emeryville, Hercules, Kensington, Oakland, Piedmont, Pinole, Richmond, and San Pablo (n~307,000 residential households) were mailed an invitation to participate. The household member aged 18 or older with the next birthday was invited to complete a consent form and screening questionnaire. Spanish versions of study invitations and all study materials were also utilized.

Significant effort was spent recruiting individuals from ZIP codes with high percentages of Latino and non-white individuals. This included sending recruitment postcards in English and Spanish to households in ZIP codes with large percentages of Spanish speakers; outreach to local high schools to notify families of the recruitment effort; fliers in English, Spanish, Chinese, Korean, Vietnamese, and Tagalog sent to local city government officials and community benefit organizations; and outreach to local Spanish radio stations and markets.

Of the 16,115 residents who consented and completed the screening procedures between May-July 2020, 1,777 individuals did not meet the inclusion criteria and were excluded. Eligible participants were required to be the household member with the next birthday, live within the study region, and be willing to provide biospecimens and questionnaire responses, read and speak English or Spanish, have a valid email address, and have internet access. The target sample size for the study phase was 5,500 participants. To obtain a sample that resembled the racial and ethnic

proportions reported in the 2018 American Community Survey (ACS) for the study region, we ranked screening participants for study inclusion. Black and/or Hispanic individuals were ranked the highest (n=1,556) followed by other non-white individuals (n=1,939). Order of inclusion for white individuals was randomly sampled. Individuals ranked between 1 and 5,500 were offered study enrollment, and non-respondents were replaced with next highest ranked individuals who had not yet been offered study entry.

Biospecimens and questionnaire data were collected during three rounds. Approximate dates for each round were July-September 2020, October-December 2020, and February-April 2021. For each round of data collection, individuals who had participated in the previous round were contacted to confirm their participation in the next round. If participation was declined, individuals from the pool of screening participants who had not yet participated in a study round were invited as needed. This resulted in 5,501 participants in Round 1, 5,603 participants in Round 2, and 4,806 participants in Round 3. This corresponded to participation rates of 76.8%, 89.8%, and 87.3% across the study round, respectively.

All participants provided their informed consent for the screening phase. All those participating in the study phase provided their informed consent for each study round. The study was approved by the University of California, Berkeley Committee on Protection of Human Subjects (2020-03-13121).

References

1 DeAngelis MM, Wang DG, Hawkins TL. Solid-phase reversible immobilization for the isolation of PCR products. *Nucleic Acids Res* 1995; **23**: 4742–3.

2 Nelson AC, Auch B, Schomaker M, *et al.* Analytical Validation of a COVID-19 qRT-PCR Detection Assay Using a 384-well Format and Three Extraction Methods. 2020.

3 Wong M, Meas MA, Adams C, *et al.* Development and Implementation of Dried Blood Spot-based COVID-19 Serological Assays for Epidemiologic Studies. *medRxiv* 2021; **Submitted November 24, 2021**: MS ID#: MEDRXIV/2021/266786.
