# Supplementary material for: Health inequities in SARS-CoV-2 infection, seroprevalence, and COVID-19 vaccination: Results from the East Bay COVID-19 study
Source: PLOS Glob Public Health. 2022 Aug 15;2(8):e0000647. doi: 10.1371/journal.pgph.0000647 (PMC10022102; doi:10.1371/journal.pgph.0000647)
Supplement: S2 File — (PDF) [file pgph.0000647.s014.pdf]

S2 File. Study questionnaires used for each data collection round.

Round 1 Questionnaire

University of California, Berkeley
SARS-CoV-2 Testing for Surveillance in the Bay Area Community Study:
Study Questionnaire

Please complete this research study questionnaire. These data will be used to help determine the extent to which individuals without COVID-19 (the disease caused by SARS-CoV-2 or "coronavirus") symptoms have been infected with the virus. It will also help us understand factors that increase or decrease risk of infection and disease and the impacts of the coronavirus pandemic.

Questions for which your responses are required are indicated by *must provide value.

SECTION 1: The following questions ask about general demographic information.

1. What is your last name?
   _____ last name

2. What is your first name?
   _____ first name

3. What is your middle name? (optional)
   _____ middle name

4. What is your date of birth? (This information is needed to participate in the study.)
   _____ (dd/mm/yyyy)

5. What is your gender?
   a. Male
   b. Female
   c. Other
   d. Don't know
   e. Refuse

Note: Please answer BOTH of the following two questions about Hispanic origins and race. For this survey, Hispanic origins are not races.

6. Do you consider yourself of Hispanic, Latino, or Spanish origin? (select all that apply)
    a. No, not of Hispanic, Latino, or Spanish origin
    b. Yes, Mexican, Mexican American, Chicano
    c. Yes, Puerto Rican
    d. Yes, Cuban
    e. Yes, another Hispanic, Latino, or Spanish origin
    f. Don't know
    g. Refuse

7. Do you consider yourself to be any of the following (select all that apply):
    a. White
    b. Black or African American
    c. American Indian or Alaska Native
    d. Asian Indian
    e. Chinese
    f. Filipino
    g. Japanese
    h. Korean
    i. Vietnamese
    j. Native Hawaiian
    k. Guamanian or Chamorro
    l. Samoan
    m. Other Pacific Islander
    n. Other
    o. Don't know
    p. Refuse

8. What was your physical sex assigned at birth?
    a. Male
    b. Female
    c. Other
    d. Don't know
    e. Refuse

9. What is the full address of your CURRENT residence?
    _____ (street, city, state, zip code)

10. Do you prefer to receive your "biospecimen kit" at a valid, alternative address other than the one specified above within the East Bay Area?
    a. Yes
    b. No
    c. Don't know

d. Refuse

11. **[If “yes” to Q10]** What is the full address of the alternative address?
_____ (street, city, state, zip code)

12. **[If “yes” to Q10]** The alternative address above is:
    a. Where I work
    b. The home of a friend or family member
    c. My church or another organization where I am a member
    d. Other
    e. Don’t know
    f. Refuse

13. What is your email address?
_________ email address

14. What is a phone number study investigators can use to contact you?
________ phone number

15. What is the highest grade or year of school you completed?
    a. Never attended school or only attended kindergarten
    b. Grades 1 through 8 (elementary)
    c. Grades 9 through 11 (some high school)
    d. Grade 12 or GED (high school graduate)
    e. College 1 year to 3 years (some college or technical school)
    f. College 4 years or more (college graduate)
    g. Don’t know
    h. Refuse

SECTION 2: The following questions ask about your exposure to SARS-CoV-2 and symptoms, testing, and diagnosis of COVID-19.

16. To the best of your knowledge, in the past TWO WEEKS, have you been in close contact (within 6 ft) with a person who is a suspected or confirmed case of COVID-19? **[If “no”, “don’t know”, or “refuse”, skip to Q20.]**
    a. Yes
    b. No
    c. Don’t know
    d. Refuse

17. **[If “yes” to Q16]** Does this person live in your home?
    a. Yes
    b. No
    c. Don’t know
    d. Refuse

18. **[If “yes” to Q16]** How many days ago was the FIRST time you had contact with the infected person?
    a. ____ number of days ago
    b. Don’t know
    c. Refuse

19. **[If “yes” to Q16]** How many days ago was the LAST time you had contact with the infected person?
    a. ____ number of days ago
    b. Don’t know
    c. Refuse

20. Have you experienced any of the following symptoms in the past TWO WEEKS? (select all that apply)
    a. Dry cough (without mucus)
    b. Coughing up mucus
    c. Painful pressure in ears
    d. Blocked nose
    e. Runny nose
    f. Sneezing
    g. Watery eyes
    h. Hoarseness
    i. Fever
    j. Sweats
    k. Chills
    l. Headache
    m. Tickles in the throat
    n. Sore throat
    o. Muscle pain
    p. Chest pain
    q. Painful sinuses (pain or pressure in the area above and below your eyes or behind your nose)
    r. Swollen glands in your neck, armpits, or underneath your ears

s. Loss of appetite
t. Difficulty breathing
u. Wheezing (a high-pitched, whistling, or rattling sound when you breathe in or out)
v. Shortness of breath
w. Diarrhea
x. Stomach pain
y. Trouble thinking
z. Trouble sleeping
aa. Fatigue (tiredness)
bb. Loss of sense of taste
cc. Loss of sense of smell
dd. Eye pain
ee. None
ff. Don't know
gg. Refuse

21. **[If responded "fever" to Q20]** Because you responded "fever" above: did you or a physician check your temperature with a thermometer?
    a. Yes
    b. No
    c. Don't know
    d. Refuse

22. **[If responded "Yes" to Q21]** Did the thermometer show a temperature above 98.6℉ (37℃)?
    a. Yes
    b. No
    c. Don't know
    d. Refuse

23. **[If responded "diarrhea" to Q20]** Because you responded "diarrhea" above: what was the greatest number of loose stools passed, or times you had to use the toilet, in a 24-hour period?
    a. Zero
    b. One or two
    c. Three to five
    d. Six or more
    e. Don't know
    f. Refuse

24. Since **[insert date of screening questionnaire completion],** have you been tested for coronavirus (COVID-19) by a physician or medical professional? **[If "no", "don't know", or "refuse", skip to Q27]**

a. Yes
b. No
c. Don’t know
d. Refuse

25. **[If “yes” to Q24]** Was your test positive or negative? **[If “negative”, “don’t know”, or “refuse”, skip to Q27]**
    a. Positive
    b. Negative
    c. Don’t know
    d. Refuse

26. **[If “positive” to Q25]** Were you diagnosed with coronavirus (COVID-19) by a physician or medical professional?
    a. Yes
    b. No
    c. Don’t know
    d. Refuse

27. Since **[insert date of screening questionnaire completion],** has anyone living in your house, besides yourself, been tested for coronavirus (COVID-19) by a physician or medical professional? **[If “no”, “don’t know”, or “refuse”, skip to Q30]**
    a. Yes
    b. No
    c. Don’t know
    d. Refuse

28. **[If “yes” to Q27]** Was their test positive or negative? **[If “negative”, “don’t know”, or “refuse”, skip to Q30]**
    a. Positive
    b. Negative
    c. Don’t know
    d. Refuse

29. **[If “positive” to Q28]** Were they diagnosed with coronavirus (COVID-19) by a physician or medical professional?

a. Yes
b. No
c. Don’t know
d. Refuse

SECTION 3: The following questions ask about social (physical) distancing and your contact with other individuals.

30. Thinking about the past 7 DAYS, for which of these activities did you leave your house? (select all that apply) **[If “Did not leave house”, “don’t know”, or “refuse”, skip to Q32]**
    a. Did not leave house
    b. Work
    c. Shopping
    d. Leisure/Exercise
    e. Medical/healthcare
    f. Care for a relative
    g. Other
    h. Don’t know
    i. Refuse

31. **[If anything other than “did not leave house”, “don’t know”, or “refuse” to Q30]** What form of transportation did you use to leave your residence? (select all that apply)
    a. Walking or biking
    b. Public transportation
    c. Personal automobile/motorcycle
    d. Car service such as Uber/Lyft or taxi
    e. Other
    f. Don’t know
    g. Refuse

Since the COVID-19 pandemic began, what has changed for you and your family or other people living in your home?

Check **YES (Me)** if you were impacted.

Check **YES (Person in Home)** if another person (or people) in your home were impacted.

Check **NO** if you and your family or other people in your home were not impacted.

Check **N/A** if the statement does not apply to you or someone in the home.

****If both* **YES (Me)** *and* **YES (Person in Home)** *are true, check both****

	Yes (me)	Yes (person in home)	No	NA	Refuse
32. Isolated or quarantined due to possible exposure to coronavirus					
33. Isolated or quarantined due to symptoms of COVID-19					
34. Isolated due to existing health conditions that increase risk of infection or disease					

35. In the past 7 DAYS, have you been to a gathering with the following (maximum) group size? (select all that apply)
    a. 10 persons
    b. 20 persons
    c. 50 persons
    d. 100 persons
    e. More than 100 persons
    f. None
    g. Don't know
    h. Refuse

36. In the past 7 DAYS, have you worn a protective mask, and if so, where? (check all that apply) **[If "no", "don't know", or "refuse", skip to 38]**
    a. Yes, inside my home
    b. Yes, at work
    c. Yes, while traveling
    d. Yes, while shopping
    e. Yes, while doing leisure activities outside

f. Yes, other
g. No
h. Don’t know
i. Refuse

37. **[If “yes” to Q36]** Was it a N95 respirator mask, specifically? (pictured below)
    a. Yes
    b. No
    c. Don’t know
    d. Refuse

SECTION 4: The following questions ask about your travel inside and outside of the United States within the past 2 weeks and since December 1, 2019.

38. Have you traveled outside of the UNITED STATES in the PAST 2 WEEKS? **[If “no”, “don’t know” or “refuse”, skip to Q40]**
    a. Yes
    b. No
    c. Don’t know
    d. Refuse

39. **[If “yes” to Q38]** Which country/countries? (select all)
    a. _____ country/countries
    b. Don't know
    c. Refuse

40. Have you traveled outside of the UNITED STATES BETWEEN DECEMBER 1, 2019 AND 2 WEEKS PRIOR TO TODAY’S DATE? **[If “no”, “don’t know” or “refuse”, skip to Q42]**
    a. Yes
    b. No
    c. Don’t know
    d. Refuse

41. **[If “yes” to Q40]** Which country/countries? (select all)
    a. _____ country/countries
    b. Don't know

c. Refuse

42. Have you traveled to another STATE OUTSIDE OF CALIFORNIA in the PAST 2 WEEKS? **[If “no”, “don’t know” or “refuse”, skip to Q44]**
    a. Yes
    b. No
    c. Don’t know
    d. Refuse

43. **[If “yes” to Q42]** Which state(s)? (select all)
    a. _____ state(s)
    b. Don't know
    c. Refuse

44. Have you traveled to another STATE OUTSIDE OF CALIFORNIA BETWEEN DECEMBER 1, 2019 AND 2 WEEKS PRIOR TO TODAY’S DATE? **[If “no”, “don’t know” or “refuse”, skip to Q46]**
    a. Yes
    b. No
    c. Don’t know
    d. Refuse

45. **[If “yes” to Q44]** Which state(s)? (select all)
    a. _____ state(s)
    b. Don't know
    c. Refuse

46. Have you traveled to another CALIFORNIA COUNTY, outside of your current California county of residence, in the PAST 2 WEEKS? **[If “no”, “don’t know” or “refuse”, skip to Q48]**
    a. Yes
    b. No
    c. Don’t know
    d. Refuse

47. **[If “yes” to Q46]** Which California county/counties? (select all)
    a. _____ county/counties

b. Don't know
c. Refuse

48. Have you traveled to another CALIFORNIA COUNTY, outside of your current California COUNTY of residence, BETWEEN DECEMBER 1, 2019 AND 2 WEEKS PRIOR TO TODAY'S DATE? **[If "no", "don't know" or "refuse", skip to Q50]**
    a. Yes
    b. No
    c. Don't know
    d. Refuse

49. **[If "yes" to Q48]** Which California county/counties? (select all)
    a. _____ county/counties
    b. Don't know
    c. Refuse

SECTION 5: The following questions ask about your health and medical conditions.

50. What is your CURRENT weight (lbs)?
    a. ____lbs
    b. Don't know
    c. Refuse

51. What is your CURRENT height?
    a. _____ft _____in
    b. Don't know
    c. Refuse

52. Would you say your general health is:
    a. Excellent
    b. Very good
    c. Good
    d. Fair
    e. Poor
    f. Don't know
    g. Refuse

53. Are you currently pregnant, and if so, which trimester?

a. Yes, first trimester (weeks 1-12)
b. Yes, second trimester (weeks 13-26)
c. Yes, third trimester (week 27 and beyond)
d. No
e. Not applicable
f. Don't know
g. Refuse

54. Have you EVER been told by a doctor, nurse, or other health professional that you have any of the following conditions? (select all that apply)

a. Asthma
b. Chronic obstructive pulmonary disease (COPD)
c. Chronic bronchitis
d. Pneumonia
e. Hypertension
f. Diabetes
g. Cardiovascular disease
h. Cerebrovascular disease (including stroke, transient ischemic attack (TIA), aneurysm, and vascular malformation)
i. Liver disease
j. Kidney disease
k. Multiple sclerosis
l. Other autoimmune disease
m. Cancer
n. Depressive disorder (including depression, major depression, dysthymia, or minor depression)
o. Other psychiatric disorder
p. None
q. Don't know
r. Refuse

55. Have you EVER been told by a doctor or other health professional that your immune system is weakened? **[If "no", "don't know", or "refuse", skip to Q57.]**

a. Yes
b. No
c. Don't know

d. Refuse

56. **[If "yes" to Q55]** Is your immune system CURRENTLY weakened?
   a. Yes
   b. No
   c. Don't know
   d. Refuse

Since the COVID-19 pandemic began, what has changed for you and your family or other people living in your home?

Check **YES (Me)** if you were impacted.

Check **YES (Person in Home)** if another person (or people) in your home were impacted.

Check **NO** if you and people in your home were not impacted.

Check **N/A** if the statement does not apply to you or someone in the home.

****If both* **YES (Me)** *and* **YES (Person in Home)** *are true, check both****

	Yes (me)	Yes (person in home)	No	NA	Refuse
57. Increase in health problems not related to COVID-19					
58. Less physical activity or exercise					
59. Overeating or eating more unhealthy foods (e.g., junk food)					
60. More time sitting down or being sedentary					
61. Important medical procedure cancelled (e.g., surgery)					
62. Unable to access medical care for a serious condition (e.g., dialysis,					

chemotherapy)					
63. Got less medical care than usual (e.g., routine or preventive care appointments)					
64. Have an elderly or disabled family member not living in the home who was unable to get the help they need					

Over the past TWO WEEKS, how often have you been bothered by the following problems?

	Not at all	Several days	More than half the days	Nearly every day	Don't know	Refuse
65. Little interest or pleasure in doing things						
66. Feeling down, depressed or hopeless						

SECTION 6: The following questions ask about your healthcare coverage and access to healthcare.

67. What is the primary source of your health care coverage?
    a. A plan purchased through an employer or union (including plans purchased through another person's employer)
    b. A plan that you or another family member buys on your own
    c. Medicare
    d. Medicaid or other state program
    e. TRICARE (formerly CHAMPUS), VA, or Military
    f. Alaska Native, Indian Health Service, Tribal Health Services
    g. Some other source

h. None (no coverage)
i. Don’t know
j. Refuse

68. Was there a time in the past 12 MONTHS when you needed to see a doctor but could not because of cost?
    a. Yes
    b. No
    c. Don’t know
    d. Refuse

69. Was there a time in the past 2 WEEKS when you needed to see a doctor for something unrelated to COVID-19 symptoms but you did not? **[If “no”, “don’t know”, or “refuse”, skip to Q71]**
    a. Yes
    b. No
    c. Don’t know
    d. Refuse

70. **[If “yes” to Q69]** What was the reason you could not see a doctor? (select all that apply)
    a. Cost
    b. Fear of COVID-19 infection
    c. Could not get an appointment
    d. No transportation
    e. Health care need was not urgent
    f. Other
    g. Don’t know
    h. Refuse

71. About how long has it been since you last visited a doctor for a routine checkup?
    a. Within the past year (anytime less than 12 months ago)
    b. Within the past 2 years (1 year but less than 2 years ago)
    c. Within the past 5 years (2 years but less than 5 years ago)
    d. 5 or more years ago
    e. Don’t know

f. Refuse

SECTION 7: The following questions ask about your employment status and economic situation.

72. What is your annual household income from all sources?
   a. Less than $25,000
   b. $25,000 to less than $35,000
   c. $35,000 to less than $50,000
   d. $50,000 to less than $75,000
   e. $75,000 to less than $100,000
   f. $100,000 or more
   g. Don't know
   h. Refuse

Which of the following best describes your CURRENT employment status and employment status PRIOR to the shelter-in-place order or other social distancing restrictions due to the COVID-19 pandemic? (check all that apply)

	Employed for wages	Self-employed	Out of work for 1 year or more	Out of work for less than 1 year	Home-maker	Student	Retired	Unable to work	Don't know	Refuse
73. CURRENT employment status										
74. Employment status PRIOR to shelter-in-place order (or other social distancing restrictions due to the COVID-19 pandemic)										

75. **[If responded "employed for wages" or "self-employed" to Q73]** What kind of work do you currently do? For example, a registered nurse, janitor, cashier, auto mechanic. (If you have more than one job, indicate your main job.)
   a. _______ answer

b. Don't know
c. Refuse

76. **[If responded "employed for wages" or "self-employed" to Q73]** What kind of business or industry do you currently work in? For example, hospital, elementary school, clothing manufacturing, restaurant. (If you have more than one job, indicate your main business or industry.)
   a. _______ answer
   b. Don't know
   c. Refuse

77. **[If responded "out of work for less than 1 year" to Q73]** What kind of work did you do? For example, a registered nurse, janitor, cashier, auto mechanic. (If you had more than one job, indicate your main job.)
   a. _______ answer
   b. Don't know
   c. Refuse

78. **[If responded "out of work for less than 1 year" to Q73]** What kind of business or industry did you work in? For example, hospital, elementary school, clothing manufacturing, restaurant. (If you had more than one job, indicate your main business or industry.)
   a. _______ answer
   b. Don't know
   c. Refuse

Since the COVID-19 pandemic began, what has changed for you and your family or other people living in your home?

Check **YES (Me)** if you were impacted.

Check **YES (Person in Home)** if another person (or people) in your home were impacted.

Check **NO** if you and your family or other people in your home were not impacted.

Check **N/A** if the statement does not apply to you or someone in the home.

****If both* **YES (Me)** *and* **YES (Person in Home)** *are true, check both****

	Yes (me)	Yes (person in home)	No	NA	Refuse

79. Laid off from job or had to close own business					
80. Reduced work hours or furloughed					
81. Had to continue to work even though in close contact with people who might be infected (e.g., customers, patients, co-workers)					

82. If you CURRENTLY work in the healthcare field, do you come in contact with patients (within 6 ft) with confirmed COVID-19 infection?
    a. Yes
    b. No
    c. Not applicable, don't work in healthcare field
    d. Don't know
    e. Refuse

83. Does anyone CURRENTLY living in your household, not including yourself, work in the healthcare field?
    a. Yes
    b. No
    c. Don't know
    d. Refuse

Since the COVID-19 pandemic began, what has changed for you and your family or other people in your home?

Check **YES (Me)** if you were impacted.

Check **YES (Person in Home)** if another person (or people) in your home were impacted.

Check **NO** if you and your family or people in your home were not impacted.

Check **N/A** if the statement does not apply to you or someone in the home.

If both **YES (Me)** and **YES (Person in Home)** are true, check both

	Yes (me)	Yes (person in home)	No	NA	Refuse
84. Unable to get enough food or healthy food					
85. Unable to pay important bills like rent or utilities					
86. Difficulty getting places due to less access to public transportation or concerns about safety					
87. Unable to get needed medications (e.g., prescriptions or over-the-counter)					

SECTION 8: The following questions ask about your smoking and tobacco use.

88. Have you EVER smoked conventional cigarettes, e-cigarettes, pipes, or cigars? **[If "no", "don't know" or "refuse", skip to Q97]**
    a. Yes
    b. No
    c. Don't know
    d. Refuse

89. **[If "yes" to Q88]** Have you smoked at least 100 "conventional" cigarettes in your ENTIRE LIFE?
    a. Yes
    b. No
    c. Don't know
    d. Refuse

90. **[If "yes" to Q88]** How often do you NOW smoke "conventional" cigarettes?
    a. Every day
    b. Some days

c. Not at all
d. Don’t know
e. Refuse

91. **[If “yes” to Q88]** Have you smoked a pipe at least 50 times in your ENTIRE LIFE?
    a. Yes
    b. No
    c. Don’t know
    d. Refuse

92. **[If “yes” to Q88]** How often do you NOW smoke a pipe?
    a. Every day
    b. Some days
    c. Not at all
    d. Don’t know
    e. Refuse

93. **[If “yes” to Q88]** Have you smoked at least 50 cigars in your ENTIRE LIFE?
    a. Yes
    b. No
    c. Don’t know
    d. Refuse

94. **[If “yes” to Q88]** How often do you NOW smoke cigars?
    a. Every day
    b. Some days
    c. Not at all
    d. Don’t know
    e. Refuse

95. **[If “yes” to Q88]** Have you EVER used an e-cigarette or other electronic vaping product, even just one time, in your entire life? **[If “no”, “don’t know” or “refuse”, skip to Q97]**
    a. Yes
    b. No
    c. Don’t know

d. Refuse

96. **[If “yes” to Q95]** How often do you <u>NOW</u> use an e-cigarette or other electronic vaping product?
    a. Every day
    b. Some days
    c. Not at all
    d. Don’t know
    e. Refuse

97. How often do you <u>CURRENTLY</u> use chewing tobacco, snuff, or snus?
    a. Every day
    b. Some days
    c. Not at all
    d. Don’t know
    e. Refuse

98. Does ANYONE smoke cigarettes, cigars, or pipes ANYWHERE INSIDE your home? **[If “no”, “don’t know” or “refuse”, skip to Q97]**
    a. Yes
    b. No
    c. Don’t know
    d. Refuse

99. **[If “yes” to Q95]** On average, how many days per week is there smoking ANYWHERE INSIDE your home?
    a. Less than 1 day per week/rarely/none
    b. 1 day
    c. 2 days
    d. 3 days
    e. 4 days
    f. 5 days
    g. 6 days
    h. 7 days
    i. Don’t know
    j. Refuse

SECTION 9: The following questions ask about your alcohol consumption.

100. During the PAST 30 DAYS, how many days PER WEEK did you have at LEAST ONE DRINK of an alcoholic beverage such as beer, wine, a malt beverage or liquor? (One drink is equivalent to a 12-ounce beer, a 5-ounce glass of wine, or a drink with one shot of liquor.) **[If "0 days", "don't know" or "refuse", skip to Q103]**
    a. ________ number of days
    b. Don't know
    c. Refuse

101. **[If any value other than "0", "don't know" or "refuse" for Q100]** During the PAST 30 DAYS, on the days when you drank, about how many drinks did you drink on average? (One drink is equivalent to a 12-ounce beer, a 5-ounce glass of wine, or a drink with one shot of liquor.)
    a. ______number of drinks
    b. None
    c. Don't know
    d. Refuse

102. **[If any value other than "0", "don't know" or "refuse" for Q100]** During the PAST 30 DAYS, what is the largest number of drinks you had on any occasion?
    a. ____ number of drinks
    b. Don't know
    c. Refuse

SECTION 10: The following questions ask about your household and home life.

103. Do you CURRENTLY own or rent your home?
    a. Own
    b. Rent
    c. Other arrangement
    d. Don't know
    e. Refuse

104. What is your CURRENT marital status?
    a. Married
    b. Living with partner

c. Separated
d. Divorced
e. Widowed
f. Single
g. Don't know
h. Refuse

105. Besides yourself, how many people live in your household? **[If “0”, “don’t know”, or “refuse” skip to end]**
    a. ________ number
    b. Don’t know
    c. Refuse

[If responded any value other than “0”, “don’t know”, or “refuse” to Q105] For each person living in your household (not including yourself), please answer the following questions:

PERSON X *(repeat questions 106-109 for each person in household)*

106. How old is PERSON X (years)?
    _______age (years)

107. Does PERSON X currently work at a job (or attend school) that requires them to work (or study) OUTSIDE of the home, despite the shelter in place order?
    a. Yes, for work
    b. Yes, for school
    c. Yes, for both work and school
    d. No
    e. Don’t know
    f. Refuse

108. **[If responded “yes, for work” or “yes, for both work and school” to Q107]** What kind of work does PERSON X currently do? For example, a registered nurse, janitor, cashier, auto mechanic. (If you have more than one job, indicate your main job.)
    a. _______ answer
    b. Don’t know
    c. Refuse

109. **[If responded “yes, for work” or “yes, for both work and school” to Q107]** What kind of business or industry does PERSON X currently work in? For

example, hospital, elementary school, clothing manufacturing, restaurant. (If you have more than one job, indicate your main business or industry.)

a. _______ answer
b. Don’t know
c. Refuse

Thank you for your participation!

Round 2 Questionnaire

University of California, Berkeley
SARS-CoV-2 Testing for Surveillance in the Bay Area Community Study:
Study Questionnaire

Please complete this research study questionnaire **on the same day you collect your blood, swab, and saliva samples**. These data will be used to help determine the extent to which individuals without COVID-19 (the disease caused by SARS-CoV-2 or "coronavirus") symptoms have been infected with the virus. It will also help us understand factors that increase or decrease risk of infection and disease and the impacts of the coronavirus pandemic.

STOP: do not complete this form before collecting your blood, swab and saliva samples.

Questions for which your responses are required are indicated by *must provide value.

SECTION 1: The following questions ask you to confirm your participation and sample information.

1. What is your last name?
   ____ last name

2. What is your first name?
   ____ first name

3. The email address we have on file for you is **[auto-insert here]**. If you would like an **alternative** email address used for communications, please provide below:
   _______ email address

4. On what date did you collect your own blood sample?

   _______ date

5. On what date did you collect your own nasal swab sample?

_______ date

6. On what date did you collect your own saliva sample?

   _______ date

SECTION 2: The following questions ask about your exposure to SARS-CoV-2 (coronavirus) and symptoms, testing, and diagnosis of COVID-19.

7. To the best of your knowledge, in the past TWO WEEKS, have you been in close contact (within 6 ft) with a person who is a suspected or confirmed case of COVID-19? **[If “no”, “don’t know”, or “refuse”, skip to Q11.]**
   a. Yes
   b. No
   c. Don’t know
   d. Refuse

8. **[If “yes” to Q7]** Does this person live in your home?
   a. Yes
   b. No
   c. Don’t know
   d. Refuse

9. **[If “yes” to Q7]** How many days ago was the FIRST time you had contact with the infected person?
   a. ____ number of days ago
   b. Don’t know
   c. Refuse

10. **[If “yes” to Q7]** How many days ago was the LAST time you had contact with the infected person?
    a. ____ number of days ago
    b. Don’t know
    c. Refuse

11. Are you CURRENTLY experiencing any of the following symptoms? (select all that apply)
    a. Dry cough (without mucus)
    b. Coughing up mucus
    c. Painful pressure in ears
    d. Blocked nose
    e. Runny nose
    f. Sneezing
    g. Watery eyes
    h. Hoarseness
    i. Fever
    j. Sweats
    k. Chills
    l. Headache
    m. Tickles in the throat
    n. Sore throat
    o. Muscle pain
    p. Chest pain
    q. Painful sinuses (pain or pressure in the area above and below your eyes or behind your nose)
    r. Swollen glands in your neck, armpits, or underneath your ears
    s. Loss of appetite
    t. Difficulty breathing
    u. Wheezing (a high-pitched, whistling, or rattling sound when you breathe in or out)
    v. Shortness of breath
    w. Diarrhea
    x. Stomach pain
    y. Trouble thinking
    z. Trouble sleeping
    aa. Fatigue (tiredness)
    bb. Loss of sense of taste
    cc. Loss of sense of smell
    dd. Eye pain
    ee. None
    ff. Don’t know
    gg. Refuse

12. **[If responded “fever” to Q11]** Because you responded “fever” above: did you or a physician check your temperature with a thermometer?
    a. Yes
    b. No
    c. Don’t know
    d. Refuse

13. **[If responded “Yes” to Q12]** Did the thermometer show a temperature above 98.6℉ (37℃)?
    a. Yes
    b. No
    c. Don’t know
    d. Refuse

14. **[If responded “diarrhea” to Q11]** Because you responded “diarrhea” above: what was the greatest number of loose stools passed, or times you had to use the toilet, in a 24-hour period?
    a. Zero
    b. One or two
    c. Three to five
    d. Six or more
    e. Don’t know
    f. Refuse

15. Have you experienced any of the following symptoms in the past TWO WEEKS (before today)? (select all that apply)
    a. Dry cough (without mucus)
    b. Coughing up mucus
    c. Painful pressure in ears
    d. Blocked nose
    e. Runny nose
    f. Sneezing
    g. Watery eyes
    h. Hoarseness
    i. Fever
    j. Sweats
    k. Chills
    l. Headache
    m. Tickles in the throat
    n. Sore throat
    o. Muscle pain
    p. Chest pain
    q. Painful sinuses (pain or pressure in the area above and below your eyes or behind your nose)
    r. Swollen glands in your neck, armpits, or underneath your ears
    s. Loss of appetite
    t. Difficulty breathing
    u. Wheezing (a high-pitched, whistling, or rattling sound when you breathe in or out)
    v. Shortness of breath
    w. Diarrhea
    x. Stomach pain

y. Trouble thinking
z. Trouble sleeping
aa. Fatigue (tiredness)
bb. Loss of sense of taste
cc. Loss of sense of smell
dd. Eye pain
ee. None
ff. Don't know
gg. Refuse

16. **[If responded "fever" to Q15]** Because you responded "fever" above: did you or a physician check your temperature with a thermometer?
    a. Yes
    b. No
    c. Don't know
    d. Refuse

17. **[If responded "Yes" to Q16]** Did the thermometer show a temperature above 98.6℉ (37℃)?
    a. Yes
    b. No
    c. Don't know
    d. Refuse

18. **[If responded "diarrhea" to Q15]** Because you responded "diarrhea" above: what was the greatest number of loose stools passed, or times you had to use the toilet, in a 24-hour period?
    a. Zero
    b. One or two
    c. Three to five
    d. Six or more
    e. Don't know
    f. Refuse

19. Since **[insert date Round 1 study questionnaire completed],** have you been tested for coronavirus (COVID-19) infection (not an antibody test) by a physician or medical professional, not including this study? **[If "no", "don't know", or "refuse", skip to Q24]**
    a. Yes
    b. No
    c. Don't know
    d. Refuse

20. **[If "yes" to Q19]** How many times were you tested?
   _______ number of times

21. **[If "yes" to Q19]** Did you test positive (if you were tested multiple times, did you receive at least one positive test result)? **[If "no", "don't know", or "refuse", skip to Q24]**
    a. Yes
    b. No
    c. Don't know
    d. Refuse

22. **[If "yes" to Q21]** For your positive test result, what date were you tested?
    _________date

23. **[If "yes" to Q21]** Were you diagnosed with coronavirus (COVID-19) by a physician or medical professional?
    a. Yes
    b. No
    c. Don't know
    d. Refuse

24. Have you ever been tested for the presence of antibodies to coronavirus (COVID-19) by a physician or medical professional (not including this study)? **[If "no", "don't know", or "refuse", skip to Q26]**
    a. Yes
    b. No
    c. Don't know
    d. Refuse

25. **[If "yes" to Q24]** Did the results show that you have antibodies to coronavirus?
    a. Yes
    b. No
    c. Don't know
    d. Refuse

26. **[If "yes" to Q25]** For your positive coronavirus antibody test result, on what date were you tested? If you had multiple positive antibody test results, select the most recent test date.

    ________ date

27. Since **[insert date Round 1 study questionnaire completed],** has anyone living in your house, besides yourself, been tested for coronavirus (COVID-19) infection (not an antibody test) by a physician or medical professional, not including this study? **[If "no", "don't know", or "refuse", skip to Q31]**
    a. Yes
    b. No
    c. Don't know
    d. Refuse

28. **[If "yes" to Q27]** Did they test positive? (If multiple household members were tested or someone was tested multiple times, was there at least one positive test result?) **[If "no", "don't know", or "refuse", skip to Q31]**
    a. Yes
    b. No
    c. Don't know
    d. Refuse

29. **[If "yes" to Q28]** For the positive test result, what date were they tested? If there were multiple positive results in your household, answer for the first positive result.

    _________date

30. **[If "yes" to Q28]** Were they diagnosed with coronavirus (COVID-19) by a physician or medical professional?
    a. Yes
    b. No
    c. Don't know
    d. Refuse

31. Have you ever participated in a coronavirus vaccine clinical trial? **[If "no", "don't know", or "refuse", skip to Q34]**

a. Yes
b. No
c. Don’t know
d. Refuse

32. **[If “yes” to Q31]** What was the date of your first vaccination/injection?
_______ date

33. **[If “yes” to Q31]** [Optional] What is the name of clinical trial/study?
_______ open text

SECTION 3: The following questions ask about social (physical) distancing and your contact with other individuals.

34. Thinking about the past 7 DAYS, for which of these activities did you leave your house? (select all that apply) **[If “Did not leave house”, “don’t know”, or “refuse”, skip to Q36]**
    a. Did not leave house
    b. Work
    c. Shopping
    d. Leisure/Exercise
    e. Medical/healthcare
    f. Care for a relative
    g. Other
    h. Don’t know
    i. Refuse

35. **[If anything other than “did not leave house”, “don’t know”, or “refuse” to Q34]** What form of transportation did you use to leave your residence? (select all that apply)
    a. Walking or biking
    b. Public transportation
    c. Personal automobile/motorcycle
    d. Car service such as Uber/Lyft or taxi
    e. Other
    f. Don’t know
    g. Refuse

36. In the past 7 DAYS, have you been to a gathering with the following (maximum) group size? (select all that apply)
    a. 10 persons
    b. 20 persons
    c. 50 persons
    d. 100 persons
    e. More than 100 persons
    f. None
    g. Don’t know
    h. Refuse

37. In the past 7 DAYS, have you worn a protective mask, and if so, where? (check all that apply) **[If “no”, “don’t know”, or “refuse”, skip to 39]**
    a. Yes, inside my home
    b. Yes, at work
    c. Yes, while traveling
    d. Yes, while shopping
    e. Yes, while doing leisure activities outside
    f. Yes, other
    g. No
    h. Don’t know
    i. Refuse

38. **[If “yes” to Q37]** Was it a N95 respirator mask, specifically? (pictured below)
    a. Yes
    b. No
    c. Don’t know
    d. Refuse

SECTION 4: The following questions ask about your travel inside and outside of the United States within the past 2 weeks and since the previous study round of coronavirus testing.

39. Have you traveled outside of the UNITED STATES in the PAST 2 WEEKS? **[If “no”, “don’t know” or “refuse”, skip to Q41]**
    a. Yes
    b. No

c. Don’t know
d. Refuse

40. **[If “yes” to Q39]** Which country/countries? (select all)
    a. _____ country/countries
    b. Don't know
    c. Refuse

41. Have you traveled outside of the UNITED STATES BETWEEN [date previous study questionnaire completed] AND 2 WEEKS PRIOR TO TODAY’S DATE? **[If “no”, “don’t know” or “refuse”, skip to Q43]**
    a. Yes
    b. No
    c. Don’t know
    d. Refuse

42. **[If “yes” to Q41]** Which country/countries? (select all)
    a. _____ country/countries
    b. Don't know
    c. Refuse

43. Have you traveled to another STATE OUTSIDE OF CALIFORNIA in the PAST 2 WEEKS? **[If “no”, “don’t know” or “refuse”, skip to Q45]**
    a. Yes
    b. No
    c. Don’t know
    d. Refuse

44. **[If “yes” to Q43]** Which state(s)? (select all)
    a. _____ state(s)
    b. Don't know
    c. Refuse

45. Have you traveled to another STATE OUTSIDE OF CALIFORNIA BETWEEN [date previous study questionnaire completed] AND 2 WEEKS PRIOR TO TODAY’S DATE? **[If “no”, “don’t know” or “refuse”, skip to Q47]**
    a. Yes

b. No
c. Don’t know
d. Refuse

46. **[If “yes” to Q45]** Which state(s)? (select all)
    a. _____ state(s)
    b. Don't know
    c. Refuse

47. Have you traveled to another CALIFORNIA COUNTY, outside of your current California county of residence, in the PAST 2 WEEKS? **[If “no”, “don’t know” or “refuse”, skip to Q49]**
    a. Yes
    b. No
    c. Don’t know
    d. Refuse

48. **[If “yes” to Q47]** Which California county/counties? (select all)
    a. _____ county/counties
    b. Don't know
    c. Refuse

49. Have you traveled to another CALIFORNIA COUNTY, outside of your current California COUNTY of residence, BETWEEN [date previous study questionnaire completed] AND 2 WEEKS PRIOR TO TODAY’S DATE? **[If “no”, “don’t know” or “refuse”, skip to Q51]**
    a. Yes
    b. No
    c. Don’t know
    d. Refuse

50. **[If “yes” to Q49]** Which California county/counties? (select all)
    a. _____ county/counties
    b. Don't know
    c. Refuse

SECTION 5: The following questions ask about your health and medical conditions.

51. What is your CURRENT weight (lbs)?
    a. ____lbs
    b. Don't know
    c. Refuse

52. Would you say your general health is:
    a. Excellent
    b. Very good
    c. Good
    d. Fair
    e. Poor
    f. Don’t know
    g. Refuse

53. Are you currently pregnant, and if so, which trimester?
    a. Yes, first trimester (weeks 1-12)
    b. Yes, second trimester (weeks 13-26)
    c. Yes, third trimester (week 27 and beyond)
    d. No
    e. Not applicable
    f. Don’t know
    g. Refuse

54. Have you EVER been told by a doctor, nurse, or other health professional that you have any of the following conditions? (select all that apply)
    a. Asthma
    b. Chronic obstructive pulmonary disease (COPD)
    c. Chronic bronchitis
    d. Pneumonia
    e. Hypertension
    f. Diabetes
    g. Cardiovascular disease
    h. Cerebrovascular disease (including stroke, transient ischemic attack (TIA), aneurysm, and vascular malformation)
    i. Liver disease
    j. Kidney disease
    k. Multiple sclerosis
    l. Other autoimmune disease

m. Cancer
n. Depressive disorder (including depression, major depression, dysthymia, or minor depression)
o. Other psychiatric disorder
p. None
q. Don't know
r. Refuse

55. Have you EVER been told by a doctor or other health professional that your immune system is weakened?
    a. Yes
    b. No
    c. Don't know
    d. Refuse

Since **[date previous study questionnaire completed]**, what has changed for you and your family or other people living in your home?

Check **YES (Me)** if you were impacted.

Check **YES (Person in Home)** if another person (or people) in your home were impacted.

Check **NO** if you and people in your home were not impacted.

Check **N/A** if the statement does not apply to you or someone in the home.

****If both* **YES (Me)** *and* **YES (Person in Home)** *are true, check both****

	Yes (me)	Yes (person in home)	No	NA	Refuse
56. Increase in health problems not related to COVID-19					
57. Less physical activity or exercise					
58. Overeating or eating more unhealthy foods (e.g., junk food)					
59. More time sitting					

down or being sedentary					
60. Important medical procedure cancelled (e.g., surgery)					
61. Unable to access medical care for a serious condition (e.g., dialysis, chemotherapy)					
62. Got less medical care than usual (e.g., routine or preventive care appointments)					
63. Have an elderly or disabled family member not living in the home who was unable to get the help they need					

Over the past TWO WEEKS, how often have you been bothered by the following problems?

	Not at all	Several days	More than half the days	Nearly every day	Don't know	Refuse
64. Little interest or pleasure in doing things						
65. Feeling down, depressed or hopeless						

SECTION 6: The following questions ask about your healthcare coverage and access to healthcare.

66. What is the primary source of your health care coverage?
    a. A plan purchased through an employer or union (including plans purchased through another person's employer)
    b. A plan that you or another family member buys on your own
    c. Medicare
    d. Medicaid or other state program
    e. TRICARE (formerly CHAMPUS), VA, or Military
    f. Alaska Native, Indian Health Service, Tribal Health Services
    g. Some other source
    h. None (no coverage)
    i. Don't know
    j. Refuse

67. Was there a time in the past 2 WEEKS when you needed to see a doctor for something unrelated to COVID-19 symptoms but you did not? **[If "no", "don't know", or "refuse", skip to Q69]**
    a. Yes
    b. No
    c. Don't know
    d. Refuse

68. **[If "yes" to Q67]** What was the reason you could not see a doctor? (select all that apply)
    a. Cost
    b. Fear of COVID-19 infection
    c. Could not get an appointment
    d. No transportation
    e. Health care need was not urgent
    f. Other
    g. Don't know
    h. Refuse

SECTION 7: The following questions ask about your employment status and economic situation.

69. What is your annual household income from all sources?
    a. Less than $25,000
    b. $25,000 to less than $35,000
    c. $35,000 to less than $50,000
    d. $50,000 to less than $75,000
    e. $75,000 to less than $100,000
    f. $100,000 or more
    g. Don't know
    h. Refuse

Which of the following best describes your CURRENT employment status? (check all that apply)

	Employed for wages	Self-employed	Out of work for 1 year or more	Out of work for less than 1 year	Home-maker	Student	Retired	Unable to work	Don't know	Refuse
70. CURRENT employment status										

71. **[If responded "employed for wages" or "self-employed" to Q70]** What kind of work do you currently do? For example, a registered nurse, janitor, cashier, auto mechanic. (If you have more than one job, indicate your main job.)
    a. _______ answer
    b. Don't know
    c. Refuse

72. **[If responded "employed for wages" or "self-employed" to Q70]** What kind of business or industry do you currently work in? For example, hospital, elementary school, clothing manufacturing, restaurant. (If you have more than one job, indicate your main business or industry.)
    a. _______ answer
    b. Don't know
    c. Refuse

73. **[If responded "out of work for less than 1 year" to Q70]** What kind of work did you do? For example, a registered nurse, janitor, cashier, auto mechanic. (If you had more than one job, indicate your main job.)

   a. _______ answer
   b. Don't know
   c. Refuse

74. **[If responded "out of work for less than 1 year" to Q70]** What kind of business or industry did you work in? For example, hospital, elementary school, clothing manufacturing, restaurant. (If you had more than one job, indicate your main business or industry.)
   a. _______ answer
   b. Don't know
   c. Refuse

Since **[date previous study questionnaire completed]**, what has changed for you and your family or other people living in your home?

Check **YES (Me)** if you were impacted.

Check **YES (Person in Home)** if another person (or people) in your home were impacted.

Check **NO** if you and your family or other people in your home were not impacted.

Check **N/A** if the statement does not apply to you or someone in the home.

****If both* **YES (Me)** *and* **YES (Person in Home)** *are true, check both****

	Yes (me)	Yes (person in home)	No	NA	Refuse
75. Laid off from job or had to close own business					
76. Reduced work hours or furloughed					
77. Had to continue to work even though in close contact with people who might be infected (e.g., customers, patients, co-workers)					

78. If you CURRENTLY work in the healthcare field, do you come in contact with patients (within 6 ft) with confirmed COVID-19 infection?
    a. Yes
    b. No
    c. Not applicable, don't work in healthcare field
    d. Don't know
    e. Refuse

79. Does anyone CURRENTLY living in your household, not including yourself, work in the healthcare field?
    a. Yes
    b. No
    c. Don't know
    d. Refuse

Since **[date previous study questionnaire completed]**, what has changed for you and your family or other people in your home?

Check **YES (Me)** if you were impacted.

Check **YES (Person in Home)** if another person (or people) in your home were impacted.

Check **NO** if you and your family or people in your home were not impacted.

Check **N/A** if the statement does not apply to you or someone in the home.

****If both* **YES (Me)** *and* **YES (Person in Home)** *are true, check both****

	Yes (me)	Yes (person in home)	No	NA	Refuse
80. Unable to get enough food or healthy food					
81. Unable to pay important bills like rent or utilities					
82. Difficulty getting places due to less access to public transportation or concerns about safety					

83. Unable to get needed medications (e.g., prescriptions or over-the-counter)					

SECTION 8: The following questions ask about your smoking and tobacco use.

84. How often do you smoke “conventional” cigarettes?
    a. Every day
    b. Some days
    c. Not at all
    d. Don’t know
    e. Refuse

85. How often do you smoke a pipe?
    a. Every day
    b. Some days
    c. Not at all
    d. Don’t know
    e. Refuse

86. How often do you smoke cigars?
    a. Every day
    b. Some days
    c. Not at all
    d. Don’t know
    e. Refuse

87. How often do you use an e-cigarette or other electronic vaping product?
    a. Every day
    b. Some days
    c. Not at all
    d. Don’t know
    e. Refuse

88. How often do you use chewing tobacco, snuff, or snus?
    a. Every day

b. Some days
c. Not at all
d. Don’t know
e. Refuse

89. Does ANYONE smoke cigarettes, cigars, or pipes ANYWHERE INSIDE your home? **[If “no”, “don’t know” or “refuse”, skip to Q91]**
    a. Yes
    b. No
    c. Don’t know
    d. Refuse

90. **[If “yes” to Q89]** On average, how many days per week is there smoking ANYWHERE INSIDE your home?
    a. Less than 1 day per week/rarely/none
    b. 1 day
    c. 2 days
    d. 3 days
    e. 4 days
    f. 5 days
    g. 6 days
    h. 7 days
    i. Don’t know
    j. Refuse

SECTION 9: The following questions ask about your alcohol consumption.

91. During the PAST 30 DAYS, how many days PER WEEK did you have at LEAST ONE DRINK of an alcoholic beverage such as beer, wine, a malt beverage or liquor? (One drink is equivalent to a 12-ounce beer, a 5-ounce glass of wine, or a drink with one shot of liquor.) **[If “0 days”, “don’t know” or “refuse”, skip to Q94]**
    a. ________ number of days
    b. Don’t know
    c. Refuse

92. **[If any value other than "0", "don't know" or "refuse" for Q91]** During the PAST 30 DAYS, on the days when you drank, about how many drinks did you drink on average? (One drink is equivalent to a 12-ounce beer, a 5-ounce glass of wine, or a drink with one shot of liquor.)
    a. ______number of drinks
    b. None
    c. Don't know
    d. Refuse

93. **[If any value other than "0", "don't know" or "refuse" for Q91]** During the PAST 30 DAYS, what is the largest number of drinks you had on any occasion?
    a. ____ number of drinks
    b. Don't know
    c. Refuse

SECTION 10: The following questions ask about your demographic, household, and home life.

94. Do you consider yourself to be any of the following (select all that apply) (note: race and ethnicity were previously collected but we are collecting more race categories here):
    a. White
    b. Black or African American
    c. American Indian or Alaska Native
    d. South Asian
    e. Chinese
    f. Filipino
    g. Japanese
    h. Korean
    i. Vietnamese
    j. Other Asian
    k. Native Hawaiian
    l. Guamanian or Chamorro
    m. Samoan
    n. Other Pacific Islander
    o. Other
    p. Don't know
    q. Refuse

95. Do you CURRENTLY own or rent your home?
    a. Own

b. Rent
c. Other arrangement
d. Don’t know
e. Refuse

96. What is your CURRENT marital status?
    a. Married
    b. Living with partner
    c. Separated
    d. Divorced
    e. Widowed
    f. Single
    g. Don't know
    h. Refuse

97. Do any dogs, cats or other small furry animals, such as a rabbit, guinea pig or hamster, live or spend time in your home? **[If “no”, “don’t know”, or “refuse” skip to Q99]**
    a. Yes
    b. No
    c. Don’t know
    d. Refuse

98. **[If “yes” for Q97]** What kind of pet is it? (select all that apply)
    a. Dog
    b. Cat
    c. Other small furry animal
    d. Don’t know
    e. Refuse

99. Besides yourself, how many people live in your household? **[If “0”, “don’t know”, or “refuse” skip to end]**
    a. ________ number
    b. Don’t know
    c. Refuse

[If responded any value other than “0”, “don’t know”, or “refuse” to Q99] For each person living in your household (not including yourself), please answer the following questions:

PERSON X *(repeat questions 100-103 for each person in household)*

100. How old is PERSON X (years)?

_______age (years)

101. Does PERSON X currently work at a job (or attend school) that requires them to work (or study) OUTSIDE of the home, despite the shelter in place order?

a. Yes, for work
b. Yes, for school
c. Yes, for both work and school
d. No
e. Don't know
f. Refuse

102. **[If responded "yes, for work" or "yes, for both work and school" to Q101]** What kind of work does PERSON X currently do? For example, a registered nurse, janitor, cashier, auto mechanic. (If you have more than one job, indicate your main job.)

a. _______ answer
b. Don't know
c. Refuse

103. **[If responded "yes, for work" or "yes, for both work and school" to Q101]** What kind of business or industry does PERSON X currently work in? For example, hospital, elementary school, clothing manufacturing, restaurant. (If you have more than one job, indicate your main business or industry.)

a. _______ answer
b. Don't know
c. Refuse

Thank you for your participation!

Round 3 Questionnaire

University of California, Berkeley
SARS-CoV-2 Testing for Surveillance in the Bay Area Community Study:
Study Questionnaire

Please complete this research study questionnaire **on the same day you collect your blood and swab samples**. These data will be used to help determine the extent to which individuals without COVID-19 (the disease caused by SARS-CoV-2 or "coronavirus") symptoms have been infected with the virus. It will also help us understand factors that increase or decrease risk of infection and disease and the impacts of the coronavirus pandemic.

STOP: do not complete this form before collecting your blood and swab samples.

Questions for which your responses are required are indicated by *must provide value.

SECTION 1: The following questions ask you to confirm your participation and sample information.

1. What is your last name?
   _____ last name

2. What is your first name?
   _____ first name

3. The email address we have on file for you is **[auto-insert here]**. If you would like an **alternative** email address used for communications, please provide below:
   ________ email address

4. On what date did you collect your own blood sample?

   ________ date

5. On what date did you collect your own nasal swab sample?

   ________ date

SECTION 2: The following questions ask about your exposure to SARS-CoV-2 (coronavirus) and symptoms, testing, and diagnosis of COVID-19.

6. To the best of your knowledge, in the past TWO WEEKS, have you been in close contact (within 6 ft) with a person who is a suspected or confirmed case of COVID-19? **[If "no", "don't know", or "refuse", skip to Q10.]**
    a. Yes
    b. No
    c. Don't know
    d. Refuse

7. **[If "yes" to Q6]** Does this person live in your home?
    a. Yes
    b. No
    c. Don't know
    d. Refuse

8. **[If "yes" to Q6]** How many days ago was the FIRST time you had contact with the infected person?
    a. ____ number of days ago
    b. Don't know
    c. Refuse

9. **[If "yes" to Q6]** How many days ago was the LAST time you had contact with the infected person?
    a. ____ number of days ago
    b. Don't know
    c. Refuse

10. Are you CURRENTLY experiencing any of the following symptoms? (select all that apply)
    a. Dry cough (without mucus)
    b. Coughing up mucus
    c. Painful pressure in ears

d. Blocked nose
e. Runny nose
f. Sneezing
g. Watery eyes
h. Hoarseness
i. Fever
j. Sweats
k. Chills
l. Headache
m. Tickles in the throat
n. Sore throat
o. Muscle pain
p. Chest pain
q. Painful sinuses (pain or pressure in the area above and below your eyes or behind your nose)
r. Swollen glands in your neck, armpits, or underneath your ears
s. Loss of appetite
t. Difficulty breathing
u. Wheezing (a high-pitched, whistling, or rattling sound when you breathe in or out)
v. Shortness of breath
w. Diarrhea
x. Stomach pain
y. Trouble thinking
z. Trouble sleeping
aa. Fatigue (tiredness)
bb. Loss of sense of taste
cc. Loss of sense of smell
dd. Eye pain
ee. None
ff. Don't know
gg. Refuse

11. **[If responded "fever" to Q10]** Because you responded "fever" above: did you or a physician check your temperature with a thermometer?
    a. Yes
    b. No
    c. Don't know
    d. Refuse

12. **[If responded "Yes" to Q11]** Did the thermometer show a temperature above 98.6℉ (37℃)?
    a. Yes
    b. No
    c. Don't know

d. Refuse

13. **[If responded "diarrhea" to Q10]** Because you responded "diarrhea" above: what was the greatest number of loose stools passed, or times you had to use the toilet, in a 24-hour period?
    a. Zero
    b. One or two
    c. Three to five
    d. Six or more
    e. Don't know
    f. Refuse

14. Have you experienced any of the following symptoms in the past TWO WEEKS (before today)? (select all that apply)
    a. Dry cough (without mucus)
    b. Coughing up mucus
    c. Painful pressure in ears
    d. Blocked nose
    e. Runny nose
    f. Sneezing
    g. Watery eyes
    h. Hoarseness
    i. Fever
    j. Sweats
    k. Chills
    l. Headache
    m. Tickles in the throat
    n. Sore throat
    o. Muscle pain
    p. Chest pain
    q. Painful sinuses (pain or pressure in the area above and below your eyes or behind your nose)
    r. Swollen glands in your neck, armpits, or underneath your ears
    s. Loss of appetite
    t. Difficulty breathing
    u. Wheezing (a high-pitched, whistling, or rattling sound when you breathe in or out)
    v. Shortness of breath
    w. Diarrhea
    x. Stomach pain
    y. Trouble thinking
    z. Trouble sleeping
    aa. Fatigue (tiredness)
    bb. Loss of sense of taste
    cc. Loss of sense of smell

dd. Eye pain
ee. None
ff. Don’t know
gg. Refuse

15. **[If responded “fever” to Q14]** Because you responded “fever” above: did you or a physician check your temperature with a thermometer?
    a. Yes
    b. No
    c. Don’t know
    d. Refuse

16. **[If responded “Yes” to Q15]** Did the thermometer show a temperature above 98.6℉ (37℃)?
    a. Yes
    b. No
    c. Don’t know
    d. Refuse

17. **[If responded “diarrhea” to Q14]** Because you responded “diarrhea” above: what was the greatest number of loose stools passed, or times you had to use the toilet, in a 24-hour period?
    a. Zero
    b. One or two
    c. Three to five
    d. Six or more
    e. Don’t know
    f. Refuse

18. Since **[insert date of previous round’s study questionnaire completed],** have you been tested for coronavirus (COVID-19) infection (not an antibody test) by a physician or medical professional, not including this study? **[If “no”, “don’t know”, or “refuse”, skip to Q23]**
    a. Yes
    b. No
    c. Don’t know
    d. Refuse

19. **[If “yes” to Q18]** How many times were you tested?
    _______ number of times

20. **[If "yes" to Q18]** Did you test positive (if you were tested multiple times, did you receive at least one positive test result)? **[If "no", "don't know", or "refuse", skip to Q23]**
    a. Yes
    b. No
    c. Don't know
    d. Refuse

21. **[If "yes" to Q20]** For your positive test result, what date were you tested?
    _________date

22. **[If "yes" to Q20]** Were you diagnosed with coronavirus (COVID-19) by a physician or medical professional?
    a. Yes
    b. No
    c. Don't know
    d. Refuse

23. Since **[insert date of previous round's study questionnaire completed],** have you been tested for the presence of antibodies to coronavirus (COVID-19) by a physician or medical professional (not including this study)? **[If "no", "don't know", or "refuse", skip to Q26]**
    a. Yes
    b. No
    c. Don't know
    d. Refuse

24. **[If "yes" to Q23]** Did the results show that you have antibodies to coronavirus?
    a. Yes
    b. No
    c. Don't know
    d. Refuse

25. **[If "yes" to Q24]** For your positive coronavirus antibody test result, on what date were you tested? If you had multiple positive antibody test results, select the most recent test date.

________ date

26. Since **[insert date of previous round's study questionnaire completed],** has anyone living in your house, besides yourself, been tested for coronavirus (COVID-19) infection (not an antibody test) by a physician or medical professional, not including this study? **[If "no", "don't know", or "refuse", skip to Q30]**
    a. Yes
    b. No
    c. Don't know
    d. Refuse

27. **[If "yes" to Q26]** Did they test positive? (If multiple household members were tested or someone was tested multiple times, was there at least one positive test result?) **[If "no", "don't know", or "refuse", skip to Q30]**
    a. Yes
    b. No
    c. Don't know
    d. Refuse

28. **[If "yes" to Q27]** For the positive test result, what date were they tested? If there were multiple positive results in your household, answer for the first positive result.
    _________date

29. **[If "yes" to Q27]** Were they diagnosed with coronavirus (COVID-19) by a physician or medical professional?
    a. Yes
    b. No
    c. Don't know
    d. Refuse

30. Have you ever participated in a coronavirus vaccine clinical trial? **[If "no", "don't know", or "refuse", skip to Q33]**
    a. Yes
    b. No

   c. Don’t know
   d. Refuse

31. **[If “yes” to Q30]** What was the date of your first vaccination/injection?

    _______ date

32. **[If “yes” to Q30]** [Optional] What is the name of clinical trial/study?

    _______ open text

33. Not including your participation in a coronavirus vaccine clinical trial, have you ever been vaccinated against coronavirus? **[If “no”, “don’t know”, or “refuse”, skip to Q38]**
    a. Yes
    b. No
    c. Don’t know
    d. Refuse

34. **[If “yes” to Q33]** What is the name of the vaccine you received?
    a. Pfizer-BioNTech COVID-19 Vaccine
    b. Moderna COVID-19 Vaccine
    c. Astrazeneca
    d. Johnson & Johnson
    e. Other
    f. Don’t know
    g. Refuse

35. **[If “other” to Q34]** What is the name of the vaccine?

    ______ (open text)

36. **[If “yes” to Q33]** What was the date of your first vaccination/injection?

    _______ date

37. **[If “yes” to Q33]** What was the date (or the expected date) of your second vaccination/injection (if applicable)?

    _______ date

38. **[If “no” to Q33]** Do you plan on getting a coronavirus vaccination as it becomes available to you? **[If “yes”, skip to Q40]**
    a. Yes
    b. No
    c. Don’t know
    d. Refuse

39. **[If “no”, “don’t know”, or “refuse” to Q38]** Do you intend to delay or refuse vaccination for any of the following reasons? (check all that apply)
    a. Already have antibodies from past infection
    b. Fast-tracked nature of the vaccine’s development
    c. Uncertainty over novel mRNA technology
    d. Currently pregnant
    e. Severe allergies
    f. Immunocompromised or other diagnosed condition that prevents you from receiving any vaccine
    g. Concerns that the vaccine may adversely impact long term health
    h. Lack of access to health services, other accessibility concerns
    i. Lack of transparency and/or publicly available information
    j. Ideological reasons
    k. Don’t like needles
    l. Other
    m. Don’t know
    n. Refuse

SECTION 3: The following questions ask about social (physical) distancing and your contact with other individuals.

40. Thinking about the past 7 DAYS, for which of these activities did you leave your house? (select all that apply) **[If “Did not leave house”, “don’t know”, or “refuse”, skip to Q42]**
    a. Did not leave house
    b. Work
    c. Shopping
    d. Leisure/Exercise

e. Medical/healthcare
f. Care for a relative
g. Other
h. Don’t know
i. Refuse

41. **[If anything other than “did not leave house”, “don’t know”, or “refuse” to Q40]** What form of transportation did you use to leave your residence? (select all that apply)
    a. Walking or biking
    b. Public transportation
    c. Personal automobile/motorcycle
    d. Car service such as Uber/Lyft or taxi
    e. Other
    f. Don’t know
    g. Refuse

42. In the past 7 DAYS, have you been to a gathering with the following (maximum) group size? (select all that apply)
    a. 10 persons
    b. 20 persons
    c. 50 persons
    d. 100 persons
    e. More than 100 persons
    f. None
    g. Don’t know
    h. Refuse

43. In the past 7 DAYS, have you worn a protective mask, and if so, where? (check all that apply) **[If “no”, “don’t know”, or “refuse”, skip to 45]**
    a. Yes, inside my home
    b. Yes, at work
    c. Yes, while traveling
    d. Yes, while shopping
    e. Yes, while doing leisure activities outside
    f. Yes, other
    g. No
    h. Don’t know

i. Refuse

44. **[If “yes” to Q43]** Was it a N95 respirator mask, specifically? (pictured below)
    a. Yes
    b. No
    c. Don’t know
    d. Refuse

SECTION 4: The following questions ask about your travel inside and outside of the United States within the past 2 weeks and since the previous study round of coronavirus testing.

45. Have you traveled outside of the UNITED STATES in the PAST 2 WEEKS? **[If “no”, “don’t know” or “refuse”, skip to Q47]**
    a. Yes
    b. No
    c. Don’t know
    d. Refuse

46. **[If “yes” to Q45]** Which country/countries? (select all)
    a. ______ country/countries
    b. Don't know
    c. Refuse

47. Have you traveled outside of the UNITED STATES BETWEEN [date previous study questionnaire completed] AND 2 WEEKS PRIOR TO TODAY’S DATE? **[If “no”, “don’t know” or “refuse”, skip to Q49]**
    a. Yes
    b. No
    c. Don’t know
    d. Refuse

48. **[If “yes” to Q47]** Which country/countries? (select all)
    a. ______ country/countries
    b. Don't know
    c. Refuse

49. Have you traveled to another STATE OUTSIDE OF CALIFORNIA in the PAST 2 WEEKS? **[If “no”, “don’t know” or “refuse”, skip to Q51]**
    a. Yes
    b. No
    c. Don’t know
    d. Refuse

50. **[If “yes” to Q49]** Which state(s)? (select all)
    a. _____ state(s)
    b. Don't know
    c. Refuse

51. Have you traveled to another STATE OUTSIDE OF CALIFORNIA BETWEEN [date previous study questionnaire completed] AND 2 WEEKS PRIOR TO TODAY’S DATE? **[If “no”, “don’t know” or “refuse”, skip to Q53]**
    a. Yes
    b. No
    c. Don’t know
    d. Refuse

52. **[If “yes” to Q51]** Which state(s)? (select all)
    a. _____ state(s)
    b. Don't know
    c. Refuse

53. Have you traveled to another CALIFORNIA COUNTY, outside of your current California county of residence, in the PAST 2 WEEKS? **[If “no”, “don’t know” or “refuse”, skip to Q55]**
    a. Yes
    b. No
    c. Don’t know
    d. Refuse

54. **[If “yes” to Q53]** Which California county/counties? (select all)
    a. _____ county/counties
    b. Don't know
    c. Refuse

55. Have you traveled to another CALIFORNIA COUNTY, outside of your current California COUNTY of residence, BETWEEN [date previous study questionnaire completed] AND 2 WEEKS PRIOR TO TODAY'S DATE? **[If "no", "don't know" or "refuse", skip to Q57]**
    a. Yes
    b. No
    c. Don't know
    d. Refuse

56. **[If "yes" to Q55]** Which California county/counties? (select all)
    a. _____ county/counties
    b. Don't know
    c. Refuse

SECTION 5: The following questions ask about your health and medical conditions.

57. What is your CURRENT weight (lbs)?
    a. ____lbs
    b. Don't know
    c. Refuse

58. Would you say your general health is:
    a. Excellent
    b. Very good
    c. Good
    d. Fair
    e. Poor
    f. Don't know
    g. Refuse

59. Are you currently pregnant, and if so, which trimester?
    a. Yes, first trimester (weeks 1-12)
    b. Yes, second trimester (weeks 13-26)
    c. Yes, third trimester (week 27 and beyond)
    d. No
    e. Not applicable

f. Don’t know
g. Refuse

60. Have you EVER been told by a doctor, nurse, or other health professional that you have any of the following conditions? (select all that apply)
    a. Asthma
    b. Chronic obstructive pulmonary disease (COPD)
    c. Chronic bronchitis
    d. Pneumonia
    e. Hypertension
    f. Diabetes
    g. Cardiovascular disease
    h. Cerebrovascular disease (including stroke, transient ischemic attack (TIA), aneurysm, and vascular malformation)
    i. Liver disease
    j. Kidney disease
    k. Multiple sclerosis
    l. Other autoimmune disease
    m. Cancer
    n. Depressive disorder (including depression, major depression, dysthymia, or minor depression)
    o. Other psychiatric disorder
    p. None
    q. Don’t know
    r. Refuse

61. Have you EVER been told by a doctor or other health professional that your immune system is weakened?
    a. Yes
    b. No
    c. Don’t know
    d. Refuse

Since **[date previous study questionnaire completed]**, what has changed for you and your family or other people living in your home?

Check **YES (Me)** if you were impacted.

Check **YES (Person in Home)** if another person (or people) in your home were impacted.

Check **NO** if you and people in your home were not impacted.

Check **N/A** if the statement does not apply to you or someone in the home.

****If both* **YES (Me)** *and* **YES (Person in Home)** *are true, check both****

	Yes (me)	Yes (person in home)	No	NA	Refuse
62. Increase in health problems not related to COVID-19					
63. Less physical activity or exercise					
64. Overeating or eating more unhealthy foods (e.g., junk food)					
65. More time sitting down or being sedentary					
66. Important medical procedure cancelled (e.g., surgery)					
67. Unable to access medical care for a serious condition (e.g., dialysis, chemotherapy)					
68. Got less medical care than usual (e.g., routine or preventive care appointments)					
69. Have an elderly or disabled family member not living in the home who was unable to get the help they need					

Over the past TWO WEEKS, how often have you been bothered by the following problems?

	Not at all	Several days	More than half the days	Nearly every day	Don't know	Refuse
70. Little interest or pleasure in doing things						
71. Feeling down, depressed or hopeless						

SECTION 6: The following questions ask about your healthcare coverage and access to healthcare.

72. What is the primary source of your health care coverage?
    a. A plan purchased through an employer or union (including plans purchased through another person's employer)
    b. A plan that you or another family member buys on your own
    c. Medicare
    d. Medicaid or other state program
    e. TRICARE (formerly CHAMPUS), VA, or Military
    f. Alaska Native, Indian Health Service, Tribal Health Services
    g. Some other source
    h. None (no coverage)
    i. Don't know
    j. Refuse

73. Was there a time in the past 2 WEEKS when you needed to see a doctor for something unrelated to COVID-19 symptoms but you did not? **[If "no", "don't know", or "refuse", skip to Q75]**
    a. Yes
    b. No
    c. Don't know

d. Refuse

74. **[If "yes" to Q73]** What was the reason you could not see a doctor? (select all that apply)
    a. Cost
    b. Fear of COVID-19 infection
    c. Could not get an appointment
    d. No transportation
    e. Health care need was not urgent
    f. Other
    g. Don't know
    h. Refuse

SECTION 7: The following questions ask about your employment status and economic situation.

75. What is your annual household income from all sources?
    a. Less than $25,000
    b. $25,000 to less than $35,000
    c. $35,000 to less than $50,000
    d. $50,000 to less than $75,000
    e. $75,000 to less than $100,000
    f. $100,000 or more
    g. Don't know
    h. Refuse

Which of the following best describes your CURRENT employment status? (check all that apply)

	Employed for wages	Self-employed	Out of work for 1 year or more	Out of work for less than 1 year	Home-maker	Student	Retired	Unable to work	Don't know	Refuse
76. CURRENT employment status										

77. **[If responded “employed for wages” or “self-employed” to Q76]** What kind of work do you currently do? For example, a registered nurse, janitor, cashier, auto mechanic. (If you have more than one job, indicate your main job.)
    a. _______ answer
    b. Don’t know
    c. Refuse

78. **[If responded “employed for wages” or “self-employed” to Q76]** What kind of business or industry do you currently work in? For example, hospital, elementary school, clothing manufacturing, restaurant. (If you have more than one job, indicate your main business or industry.)
    a. _______ answer
    b. Don’t know
    c. Refuse

79. **[If responded “out of work for less than 1 year” to Q76]** What kind of work did you do? For example, a registered nurse, janitor, cashier, auto mechanic. (If you had more than one job, indicate your main job.)
    a. _______ answer
    b. Don’t know
    c. Refuse

80. **[If responded “out of work for less than 1 year” to Q76]** What kind of business or industry did you work in? For example, hospital, elementary school, clothing manufacturing, restaurant. (If you had more than one job, indicate your main business or industry.)
    a. _______ answer
    b. Don’t know
    c. Refuse

Since **[date previous study questionnaire completed]**, what has changed for you and your family or other people living in your home?

Check **YES (Me)** if you were impacted.

Check **YES (Person in Home)** if another person (or people) in your home were impacted.

Check **NO** if you and your family or other people in your home were not impacted.

Check **N/A** if the statement does not apply to you or someone in the home.

****If both **YES (Me)** and **YES (Person in Home)** are true, check both****

	Yes (me)	Yes (person in home)	No	NA	Refuse
81. Laid off from job or had to close own business					
82. Reduced work hours or furloughed					
83. Had to continue to work even though in close contact with people who might be infected (e.g., customers, patients, co-workers)					

84. If you CURRENTLY work in the healthcare field, do you come in contact with patients (within 6 ft) with confirmed COVID-19 infection?
    a. Yes
    b. No
    c. Not applicable, don't work in healthcare field
    d. Don't know
    e. Refuse

85. Does anyone CURRENTLY living in your household, not including yourself, work in the healthcare field?
    a. Yes
    b. No
    c. Don't know
    d. Refuse

Since **[date previous study questionnaire completed]**, what has changed for you and your family or other people in your home?

Check **YES (Me)** if you were impacted.

Check **YES (Person in Home)** if another person (or people) in your home were impacted.

Check **NO** if you and your family or people in your home were not impacted.

Check **N/A** if the statement does not apply to you or someone in the home.

****If both* **YES (Me)** *and* **YES (Person in Home)** *are true, check both****

	Yes (me)	Yes (person in home)	No	NA	Refuse
86. Unable to get enough food or healthy food					
87. Unable to pay important bills like rent or utilities					
88. Difficulty getting places due to less access to public transportation or concerns about safety					
89. Unable to get needed medications (e.g., prescriptions or over-the-counter)					

SECTION 8: The following questions ask about your smoking and tobacco use.

90. How often do you smoke "conventional" cigarettes?
    a. Every day
    b. Some days
    c. Not at all
    d. Don't know
    e. Refuse

91. How often do you smoke a pipe?
    a. Every day
    b. Some days
    c. Not at all
    d. Don't know

    e. Refuse

92. How often do you smoke cigars?
    a. Every day
    b. Some days
    c. Not at all
    d. Don’t know
    e. Refuse

93. How often do you use an e-cigarette or other electronic vaping product?
    a. Every day
    b. Some days
    c. Not at all
    d. Don’t know
    e. Refuse

94. How often do you use chewing tobacco, snuff, or snus?
    a. Every day
    b. Some days
    c. Not at all
    d. Don’t know
    e. Refuse

95. Does ANYONE smoke cigarettes, cigars, or pipes ANYWHERE INSIDE your home? **[If “no”, “don’t know” or “refuse”, skip to Q97]**
    a. Yes
    b. No
    c. Don’t know
    d. Refuse

96. **[If “yes” to Q95]** On average, how many days per week is there smoking ANYWHERE INSIDE your home?
    a. Less than 1 day per week/rarely/none
    b. 1 day
    c. 2 days
    d. 3 days
    e. 4 days

f. 5 days
g. 6 days
h. 7 days
i. Don’t know
j. Refuse

SECTION 9: The following questions ask about your alcohol consumption.

97. During the PAST 30 DAYS, how many days PER WEEK did you have at LEAST ONE DRINK of an alcoholic beverage such as beer, wine, a malt beverage or liquor? (One drink is equivalent to a 12-ounce beer, a 5-ounce glass of wine, or a drink with one shot of liquor.) **[If “0 days”, “don’t know” or “refuse”, skip to Q100]**
    a. ________ number of days
    b. Don’t know
    c. Refuse

98. **[If any value other than “0”, “don’t know” or “refuse” for Q97]** During the PAST 30 DAYS, on the days when you drank, about how many drinks did you drink on average? (One drink is equivalent to a 12-ounce beer, a 5-ounce glass of wine, or a drink with one shot of liquor.)
    a. ______number of drinks
    b. None
    c. Don’t know
    d. Refuse

99. **[If any value other than “0”, “don’t know” or “refuse” for Q97]** During the PAST 30 DAYS, what is the largest number of drinks you had on any occasion?
    a. ____ number of drinks
    b. Don’t know
    c. Refuse

SECTION 10: The following questions ask about your demographic, household, and home life.

100. Do you consider yourself to be any of the following (select all that apply) (note: race and ethnicity were previously collected but we are collecting more race categories here):

a. White
b. Black or African American
c. American Indian or Alaska Native
d. South Asian
e. Chinese
f. Filipino
g. Japanese
h. Korean
i. Vietnamese
j. Other Asian
k. Native Hawaiian
l. Guamanian or Chamorro
m. Samoan
n. Other Pacific Islander
o. Other
p. Don’t know
q. Refuse

101. Do you consider yourself of Hispanic, Latino, or Spanish origin? (select all that apply)
a. No, not of Hispanic, Latino, or Spanish origin
b. Yes, Mexican, Mexican American, Chicano
c. Yes, Puerto Rican
d. Yes, Cuban
e. Yes, another Hispanic, Latino, or Spanish origin
f. Don’t know
g. Refuse

102. Do you CURRENTLY own or rent your home?
a. Own
b. Rent
c. Other arrangement
d. Don’t know
e. Refuse

103. What is your CURRENT marital status?
a. Married
b. Living with partner
c. Separated
d. Divorced
e. Widowed
f. Single

g. Don't know
h. Refuse

104. Do any dogs, cats or other small furry animals, such as a rabbit, guinea pig or hamster, live or spend time in your home? **[If “no”, “don’t know”, or “refuse” skip to Q106]**
a. Yes
b. No
c. Don’t know
d. Refuse

105. **[If “yes” for Q104]** What kind of pet is it? (select all that apply)
a. Dog
b. Cat
c. Other small furry animal
d. Don’t know
e. Refuse

106. Besides yourself, how many people live in your household? **[If “0”, “don’t know”, or “refuse” skip to end]**
a. ________ number
b. Don’t know
c. Refuse

[If responded any value other than “0”, “don’t know”, or “refuse” to Q106] For each person living in your household (not including yourself), please answer the following questions:

PERSON X *(repeat questions 107-110 for each person in household)*

107. How old is PERSON X (years)?
_______age (years)

108. Does PERSON X currently work at a job (or attend school) that requires them to work (or study) OUTSIDE of the home, despite the shelter in place order?
a. Yes, for work
b. Yes, for school
c. Yes, for both work and school
d. No
e. Don’t know
f. Refuse

109. **[If responded “yes, for work” or “yes, for both work and school” to Q108]** What kind of work does PERSON X currently do? For example, a registered nurse, janitor, cashier, auto mechanic. (If you have more than one job, indicate your main job.)

a. _______ answer
b. Don’t know
c. Refuse

110. **[If responded “yes, for work” or “yes, for both work and school” to Q108]** What kind of business or industry does PERSON X currently work in? For example, hospital, elementary school, clothing manufacturing, restaurant. (If you have more than one job, indicate your main business or industry.)

a. _______ answer
b. Don’t know
c. Refuse

Thank you for your participation!
