# Supplementary material for: Health inequities in SARS-CoV-2 infection, seroprevalence, and COVID-19 vaccination: Results from the East Bay COVID-19 study
Source: PLOS Glob Public Health. 2022 Aug 15;2(8):e0000647. doi: 10.1371/journal.pgph.0000647 (PMC10022102; doi:10.1371/journal.pgph.0000647)
Supplement: S3 File — (PDF) [file pgph.0000647.s015.pdf]

S3. File. Details on data collection and SARS-CoV-2 outcome definitions.

1 Data

1.1 Study questionnaire

Participants were asked to complete an online questionnaire at the time biospecimens were collected for each round. The questionnaire included demographics, tobacco and alcohol use, income, employment, physical and mental health, medical conditions, healthcare coverage, COVID-19 symptoms, and past COVID-19 testing. Questions about the participant's household such as number of members were also collected. Screening and study questionnaire data were collected using REDCap.

1.2 SARS-CoV-2 outcomes

For each study round, we considered three separate outcomes for analyses, (1) cumulative SARS-CoV-2 antibody positivity, (2) self-reported SARS-CoV-2 viral positivity from RT-qPCR testing, (3) and the standardized surveillance case definition of "probable COVID-19" based on self-reported symptoms, close contact with others who were infected, and viral testing. See below supplement methods for description of outcome definitions.

1.2.1 SARS-CoV-2 seropositivity

Broadly, SARS-CoV-2 seropositivity is defined as the presence of antibodies against SARS-CoV-2. Antibody assay testing algorithms implemented in this research were updated during the

study to reflect changing conditions of the pandemic (Figure S-1, Table S-5). Briefly, in Round 1, samples with a signal/cutoff (S/C) value ≥1 on the Ortho-VITROS assay were considered to reflect previous infection by SARS-CoV-2. In Round 2, samples with both Ortho-VITROS S/C≥0.7 and ELISA optical density (OD) ≥0.34 were considered have antibodies from SARS-CoV-2 infection. During Round 3 vaccinations were widely available, therefore we targeted anti-bodies to the SARS-CoV-2 nucleocapsid (NC) protein which is specific to natural infection not vaccination: samples with Ortho-VITROS S/C≥0.7 and ROCHE S/C ≥ 0.0465 were considered to have antibodies from SARS-CoV-2 infection. Samples with Ortho-VITROS S/C≥0.7, ELISA optical density (OD) ≥0.34, and ROCHE S/C < 0.0465 were considered to have antibodies from COVID-19 vaccination alone.

To identify the cumulative seroprevalence of SARS-CoV-2 within our study region, cumulative seropositivity was defined as having detectable SARS-CoV-2 antibodies in the current and/or previous round(s) and cumulative seronegative was defined as not having detectable SARS-CoV-2 antibodies in the current and previous study rounds.

1.2.2 Self-reported COVID-19 test positivity

During each study round, participants were asked whether they had been tested for coronavirus (COVID-19) by a physician or medical professional since the previous data collection period and whether any test had been positive for SARS-CoV-2. Within each round, all participants who re-ported being tested were included the denominator; those who reported having a positive result for that test(s) were included in the numerator. Within a study round, self-reported COVID-19 test prevalence was defined as the proportion of those reporting a positive test out of all partici-pants who reported having a test.

1.2.3 Probable COVID-19

We used the Council for State and Territorial Epidemiologists (CSTE) standardized case definition of "probable COVID-19" which is intended for public health surveillance purposes.[1] Individuals were classified as a "probable COVID-19" case if, within the previous 14 days, they reported being within six feet of a confirmed case of COVID-19 and reported at least two of the following symptoms: fever (measured or subjective), chills, rigors, myalgia, headache, sore throat, nausea, vomiting, diarrhea, fatigue, congestion, or runny nose. Within a study round, probable COVID-19 case prevalence was defined as the proportion of those being identified as a probable COVID-19 case out of all participants who provided responses to questions about recent close contact history and symptoms.

References

1 Council of State and Territorial Epidemiologists. Standardized surveillance case definition and national notification for 2019 novel coronavirus disease (COVID-19). 2020.
