# Supplementary material for: Health inequities in SARS-CoV-2 infection, seroprevalence, and COVID-19 vaccination: Results from the East Bay COVID-19 study
Source: PLOS Glob Public Health. 2022 Aug 15;2(8):e0000647. doi: 10.1371/journal.pgph.0000647 (PMC10022102; doi:10.1371/journal.pgph.0000647)
Supplement: S4 File — (PDF) [file pgph.0000647.s016.pdf]

S4 File. Statistical methods used for population adjusted prevalence analyses.

1 Population-adjusted seroprevalence and other SARS-CoV-2 outcomes

1.1 Summary of MRP

Bayesian multilevel regression and poststratification (MRP) was used to estimate population adjusted cumulative seroprevalence, self-reported SARS-CoV-2 viral positivity at each study round, and "probable COVID-19" at each study round. MRP is a regression-based method for estimating average and sub-average effects from survey data. MRP has been shown to perform better than survey weighting methods particularly when working with small sample sizes and sparse data. The hierarchical regression model achieves this by pooling information across similar observed characteristics in the model. Details of MRP are described here[1,2]. Briefly, the steps for MRP used in this study were as follows:

1. Identify demographic and geographic characteristics of interest in the study population and construct a table with the population size, N_j, for each stratum J for all combinations of characteristics of interest.
2. Model outcome of interest as a function of demographic and geographic characteristics with the survey data using a multilevel regression model.
3. Predict probability of the outcome, $\hat{\pi}_j$, within each stratum j using the fitted multi-level model.

4. Use $\hat{\pi}_j$ as post-stratification weights and aggregate predictions from strata to population or sub-population of interest.

$$\hat{\pi}_{pop} = \frac{\sum_{j \in J} N_j \hat{\pi}_j}{\sum_{j \in J} N_j}$$

1.2 Synthetic population and poststratification

In this study, demographic variables of interest were sex, age, race, Hispanic ethnicity, income, education, household size, and ZIP code. However, cross-tabulations for these variables were not available from a single data source for our study region. We used a method described by Leeman et al to generate a synthetic population using all of the variables described above for the post stratification step.[2] The American Community Survey (ACS) provides cross tabulations for sex, age, and race within ZIP Code Tabulation Areas (ZCTAs). Marginal distributions for Hispanic ethnicity, education, income, and household size were available at the ZCTA level. Synthetic populations were generated by combining sex, age, race, and ZCTA joint distributions and marginal distributions of Hispanic ethnicity, education, and income within ZCTA. Joint distributions at the individual level for these variables were extracted from the Public Use Microdata Sample (PUMS) for Public Use Microdata Areas within our study region. The joint distribution of these seven variables yielded the following number of strata,

$$J = J_{ZIP} \times J_{sex} \times J_{age} \times J_{race/eth} \times J_{edu} \times J_{income} \times J_{HHsize} 31 \times 2 \times 5 \times 6 \times 2 \times 2 \times 6 = 44{,}640 \ strata$$

1.3 Multilevel Regression model

At each study round, SARS-CoV-2 outcomes were modeled as a function of geographic and demographic characteristics using logistic regression,

$$y_i = logit^{-1}(\beta_0 + \beta_1 male_i + \beta_2 X_{zip[i]} + \alpha_{eth[i]} + \alpha_{age[i]} + \alpha_{edu[i]} + \alpha_{income[i]} + \alpha_{hh[i]} + \phi_{zip[i]} + \alpha_{zip.eth[i]} + \alpha_{zip.edu[i]} + \alpha_{eth.edu[i]} + \alpha_{eth.income[i]} + \alpha_{eth.age[i]},$$

where y_i is the binary response variable (e.g., 1=Reactive for SARS-CoV2 antibodies, 0=non-reactive), male is a binary indicator for a participant i reporting being male or female, X_{ZIP} is the proportion of households that report being Hispanic in the ZIP code of participant i. Random intercepts, α, were defined for each category of race/ethnicity (African American/Black; white; Hispanic; American Indian or Other; Asian or Pacific Islander; and two or more races), age in years (18-29, 30-44, 45-64, 65-74, 75+), education (bachelor's degree or not), income (2019 household income > $100,000 USD), household size (1, 2, 3, 4, 5, or ≥6 persons living in household), and 31 ZIP codes within study region as well as for interactions between ZIP code and race/ethnicity, ZIP code and education, race/ethnicity and education, race/ethnicity and income, and race/ethnicity and age were included. Based on the assumption that individuals of similar ages (e.g. those aged 18-29 and 30-44 vs. 65-74 and >75) may have similar behaviors and/or underlying infection risk, structured priors were used for age groups to allow for correlation between age groups.[3]

We allowed for spatial correlations at the ZIP code level, $\phi_{zip[i]}$,using the modified Besag-York-Mollié (BYM2) model.[4,5] The scale parameter for the BYM2 model was calculated with INLA::inla.scale.model in R using neighborhood graph data for our study region ZIP codes.[6] The BYM2 model is a reparameterization of the of the original Besag-York-Mollié (BYM) model

which used random intercepts for non-spatial heterogeneity and Intrinsic Conditional Auto-regressive (ICAR) components for spatial smoothing. The BYreM2 model replaces the random intercepts and ICAR components with a single parameter which is a weighted combination of the ICAR component, ϕ , and $\theta,$ a parameter representing non-spatial heterogeneity:

$$\phi_j = \sigma\left(\phi_j\ \sqrt{\rho/s} + \theta\sqrt{1-\rho}\right)$$

$$\phi_j\ \vee \phi_{i\sim j} \sim N\left(\frac{\sum_{i\sim j}\phi_i}{d_i}, \frac{1}{d_i}\right)$$

$$\theta *\sim N\left(0, I_{N_j}\right)$$

$$\rho{\sim}U(0,1),\ \ \sigma{\sim}U(0,10),\ \ j = 31$$

1.4 Race/ethnicity categories

Self-reported race and Hispanic ethnicity were collapsed into six categories: non-Hispanic White, non-Hispanic Black, Hispanic, Asian, and AMI/other. Participants who identified as either Mexican, Mexican American, Chicano, Puerto Rican, Cuban, or Other Hispanic were categorized as Hispanic. Participants who identified as South Asian, Chinese, Filipino, Japanese, Korean, Vietnamese, other Asian, Native Hawaiian, Guamanian or Chamorro, Samoan, or Other Pacific Islander were categorized as Asian or Pacific Islander. Participants who identified as American Indian, Alaskan Native, two or more, other, refuse, or don't know and non-Hispanic were categorized as American Indian or other.

1.5 SARS-CoV-2 outcome prevalence across study region and within subgroups

We report population adjusted prevalence of our COVID-19 outcomes across the study region and within subgroups of our primary geographic and demographic variables of interest: ZIP code, sex, age, race/ethnicity, education, income, and household size. Posterior distributions of the prevalence outcomes for each poststratification strata were used to calculate the prevalence across the study region and within strata of interest. The mean of the posterior distribution of the poststratification estimates was used as the point estimate and 95% credible interval (95% CI) were the 2.5% and 97.5% quantiles of a posterior distribution. Density plots were used to verify that posterior distributions were unimodal.

1.6 SARS-CoV-2 test-kit bias corrected seroprevalence

In addition to SARS-CoV-2 seroprevalence estimates, we also estimated cumulative SARS-CoV-2 seroprevalence at each study round adjusted for the net sensitivity and specificity of the SARS-CoV-2 antibody testing algorithm by incorporating an expression for the probability of testing seropositive as a function of net sensitivity and specificity of the testing algorithm and the seropositive prevalence in the population:[7]

$$p = \pi Se + (1 - \pi)(1 - Sp),$$

where p is the expected frequency of positive tests, π, is the population prevalence, Se is the sensitivity of the assay, and Sp is the specificity of the assay. Data on sensitivity and specificity for

the antibody assays used these analyses are from validation studies reported by Wong et al (See Table S-5).[8]

1.6.1 Net sensitivity and specific for assays within a study round

In study rounds 2 and 3, serial testing was implemented to improve the sensitivity of the Ortho VITROS Anti-SARS-CoV-2 Total Ig test (ORTHO). The follow expressions for the net sensitivity and specificity of a serial testing algorithm were used to combine sensitivity and specificity of each assay in the serial algorithm[9]:

$$Se_{net} = Se_1 Se_2$$

$$Sp_{net} = Sp_1 + Sp_2 - Sp_1 Sp_2,$$

where Se_{net} and Sp_{net} are the net sensitivity and specific of the serial testing algorithm, Se_1 and Sp_1 are the sensitivity and specificity of the first test, and Se_2 and Sp_2 are the sensitivity and specificity of the second test.

1.6.2 Cumulative assay sensitivity and specificity

Because we were interested in cumulative seroprevalence, defined as testing positive for antibodies to SARS-CoV-2 in the current or previous study rounds, we needed to account for the sensitivity and specificity in assays. For example, in Round 2, study participants were antibody positive if antibodies were detected in Round 1 and/or Round 2. In Round 3, study participants were antibody positive if antibodies were detected in Round 1, Round 2, and/or Round 3. Combining tests from two different rounds in this context is straight-forward under the criteria that if either test is positive, then the participant is positive for SARS-CoV-2 antibodies.[9] See table below for

expressions for sensitivity and specificity for cumulative antibody positivity attributable to natural infection.

**Rounds tested**	**Sensitivity**	**Specificity**
Round 1 only	Se^{R1}	Sp^{R1}
Round 2 only	Se^{R2}	Sp^{R2}
Round 3 only	Se^{R3}	Sp^{R3}
Rounds 1 and 2	$Se^{R1} + Se^{R2} - (Se^{R1}Se^{R2})$	$Sp^{R1}Sp^{R2}$
Rounds 1, 2, and 3	$Se^{R1} + Se^{R2} + Se^{R3} - (Se^{R1}Se^{R2}) - (Se^{R1}Se^{R3}) - (Se^{R2}Se^{R3}) + (Se^{R1}Se^{R2}Se^{R3})$	$Sp^{R1}Sp^{R2}Sp^{R3}$

Abbreviations: Se, sensitivity; Sp, specificity; R, Round.

In Round 3, DBS samples that tested positive for antibodies to SARS-CoV-2 spike protein and negative to the SARS-CoV-2 nucleocapsid protein were considered to have antibodies from COVID-19 vaccination only. Therefore, a sample need to be positive for anti-spike antibodies using serial testing with Ortho S and ELISA S assays and negative for anti-nucleocapsid antibodies with Roche NC assay. The expressions for sensitivity and specificity for COVID-19 antibody positivity were as follows:

$$Se_{Spike} = Se_{Ortho}Se_{ELISA}$$

$$Sp_{spike} = Se_{Ortho} + Se_{ELISA} - Se_{Ortho}Se_{ELISA}$$

$$Se_{V} = Se_{Spike} + Se_{Roche} - Se_{Spike}Se_{Roche}$$

$$Sp_{V} = Sp_{spike}Sp_{Roche}$$

where Se_{Spike} and Sp_{spike} are the net sensitivity and specificity for the serial testing algorithm using the Ortho S and ELISA S assays, Se_{Roche} and Sp_{Roche} are the sensitivity and specificity of the Roche NC assay, and Se_{V} and Sp_{V} are the net sensitivity and specificity of testing positive for anti-spike antibodies and negative for anti-nucleocapsid antibodies.

See Table below for estimates of net sensitivity and specificity from test-bias analyses implemented in NIMBLE.

**Testing period**	**SARS-CoV-2 Outcome**[a]	**Rounds included for antibody testing**[b]	**Net Sensitivity % (95% CI)**[c]	**Net Specificity % (95% CI)**[c]
Round 1	SARS-COV-2 Natural Infection	R1	79.91 (66.01, 90.78)	99.57 (99.3, 99.79)
Rounds 1-2	SARS-COV-2 Natural Infection	R1	80.65 (67.23, 91.03)	99.48 (99.16, 99.75)
		R2	86.5 (75.63, 94.24)	100 (99.98, 100)
		Net R1+R2	97.38 (94.2, 99.18)	99.48 (99.16, 99.75)
Rounds 1-3	SARS-COV-2 Natural Infection	R1	80.54 (67.1, 91.06)	99.2 (97.68, 99.82)
		R2	86.37 (75.22, 94.24)	100 (99.98, 100)
		R3	76.84 (62.88, 88.1)	99.98 (99.94, 100)
		R1+R2	97.35 (94.15, 99.17)	99.19 (97.67, 99.82)
		R1+R2+R3	99.39 (98.48, 99.84)	97.33 (94.14, 99.15)
Round 3	COVID-19 Vaccination	R3	79.61 (68.26, 89.89)	99.99 (99.99, 99.99)

[a]SARS-CoV-2 outcomes for antibody test bias analyses were: 1) cumulative SARS-CoV-2 natural infection antibody positivity, defined as testing antibody positive in the current or previous round, and 2) COVID-19 vaccination antibody positivity, defined as testing positive for the SARS-CoV-2 spike protein and testing negative for the SARS-CoV-2 nucleocapsid protein in Round 3
[b]R1=Tested in Round 1 only, R2=Tested in Round 2 only, R3=Tested in Round 3 only, R1+R2=Tested in Rounds 1 and 2; R1+R2+R3=Tested in Rounds 1, 2, and 3.
[c]Net sensitivity and specificity were estimated within MRP models using NIMBLE. See Supplemental Methods section 6 for details.

1.7 Mitigation analysis

The association between LCA cluster variable classes (high-risk vs. low-risk behavior) and seroprevalence and self-reported test positivity was estimated using the MRP model described above with random intercepts for the binary cluster variable levels and interactions between the cluster variable and ZIP code, age, race/ethnicity, education, and income. Prevalence differences (PD) and prevalence ratios (PR) were estimated by predicting the probability of the outcome given the LCA class and computing the difference or ratio,

$$PD = P(Y = 1|A = 1) - P(Y = 1|A = 0)$$

$$PR = P(Y = 1|A = 1) \,/\, P(Y = 1|A = 0),$$

where Y is the binary antibody or self-test SARS-CoV-2 infection variable (1=SARS-CoV-2 positive, 0=SARS-CoV-2 negative) and A is the binary LCA cluster variable (1="high-risk", 0="low-risk").

1.7.1 Covariate imputation

Missing covariate data was imputed with the median or mean value within a ZIP code if the missingness was less than <1% (sex, age, race/ethnicity, education, and household size). Data on income with missing in approximately 3-4% in the data depending on the study round. Binary household income (1: ≥$100,000, 0: <$100,000) was imputed within each nimble model using a logistic regression likelihood with sex, percent Hispanic within a ZIP code, and education as explanatory variables.

1.8 Markov chain Monte Carlo (MCMC)

NIMBLE[10,11] was used to implement all MRP models using MCMC sampling. For adjusted prevalence outcomes we used 30,000 total iterations with 10,000 burn-in iterations across 10 chains, with a thinning interval of five. Seroprevalence test-bias models ran for 50,000 total iterations with 10,000 burn-in iterations across 10 chains, with a thinning interval of 10. Thinning was used due to memory constraints. We performed several MCMC diagnostics including

estimation of effective sample size, visual inspection of trace and density plots for each parameter of interest, Gelman and Rubin's convergence diagnostic, and autocorrelation plots.

1.8.1 Priors

Random intercepts and fixed effect priors

The prior for a vector of random intercepts for a variable V with K categories was:

$$\alpha_{v[k]} \sim N\left(0, \sigma_{v[k]}\right)$$

$$\sigma_{v[k]} \sim N_{[0,\infty)}(0, 0.5) \text{ for } k \text{ in } 1..K.$$

The prior for a fixed effect coefficient for a binary variable V was $\beta_v \sim N(0, 0.5)$. The prior for a fixed effect coefficient for a continuous variable V was $\beta_v \sim N(0, 0.5/\sigma_v)$. The structured prior[3] for the five-level categorical age variable was:

$$\alpha_j^{Age} | \alpha_{j-1}^{Age}, \ldots, \alpha_1^{Age}, \alpha^{Age} \sim N\left(\alpha_{j-1}^{Age}, \left(\sigma^{Age}\right)^2\right),$$

$$\text{for } j = 2, \ldots, 5$$

$$\sigma^{Age} \sim N_{(0,\infty)}(0,1),$$

$$\sum_{j=1}^{J} \alpha_j^{Age} = 0.$$

Antibody test-bias priors

We adapted our specifications for the sensitivity and specificity of the SARS-CoV-2 antibody assays from Carpenter and Gelman 2020.[7] The number of positive tests positive tests for assay k was binomially distributed:

$$y_k \sim Binomial(p_k, n_k)$$

$$logit(p_k) \sim N(\mu_k, \sigma_k),$$

where y_k is the number of positive tests in n_k tests observed for assay k, p_k is the probability of observing $y_k$positive tests in n_k tests. p_k has a normal prior, μ_k and σ_k are not random variables, they are specified to match the sensitivity and specificity point estimate and confidence from validation studies performed by Wong et al (Table S-5).[8]

1.9 Interpretation of 95% credible intervals

The interpretation of a 95% credible interval is: there is a 95% probability that the true (unknown) value (e.g. prevalence difference) lies within the credible interval, given the evidence provided by the observed data and statiscal model. Example, if a null value for a measure of association is 0, and the 95% credible interval for that measure of association is (0, 1.2), one could say that there is a 95% probability that the true value lies within this credible interval, given the observed data and statistical model. The "significance" of the prevalence differences reported in this research can be interpreted by checking to see if the credible intervals contain the NULL value. If a 95% credible interval does not contain the NULL value, then there is a >95% probability that the true parameter is not null, given the evidence provided by the observed data.

1.10 Nimble Models and Diagnostics

Nimble model code, priors, and MCMC diagnostic results are available at https://github.com/adams-cam/ebcovid_prev.

References

1 Gelman A, Little TC. Poststratification Into Many Categories Using Hierarchical Logistic Regression. *Survey Research* 1997; **23**: 127–35.

2 Leemann L, Wasserfallen F. Extending the Use and Prediction Precision of Subnational Public Opinion Estimation: EXTENDING USE AND PRECISION OF MrP. *American Journal of Political Science* 2017; **61**: 1003–22.

3 Gao Y, Kennedy L, Simpson D, Gelman A. Improving Multilevel Regression and Poststratification with Structured Priors. *Bayesian Analysis* 2021; **1**: 1–26.

4 Riebler A, Sørbye SH, Simpson D, Rue H. An intuitive Bayesian spatial model for disease mapping that accounts for scaling. *arXiv:160101180 [stat]* 2016; published online Jan 6. http://arxiv.org/abs/1601.01180 (accessed Sept 15, 2021).

5 Simpson D, Rue H, Riebler A, Martins TG, Sørbye SH. Penalising Model Component Complexity: A Principled, Practical Approach to Constructing Priors. *Statist Sci* 2017; **32**. DOI:10.1214/16-STS576.

6 van Niekerk J, Bakka H, Rue H, Schenk O. New frontiers in Bayesian modeling using the INLA package in R. *arXiv:190710426 [stat]* 2019; published online July 25. http://arxiv.org/abs/1907.10426 (accessed Sept 15, 2021).

7 Gelman A, Carpenter B. Bayesian analysis of tests with unknown specificity and sensitivity *. *medRxiv* 2020; : 2020.05.22.20108944-2020.05.22.20108944.

8 Wong M, Meas MA, Adams C, *et al.* Development and Implementation of Dried Blood Spot-based COVID-19 Serological Assays for Epidemiologic Studies. *medRxiv* 2021; **Submitted November 24, 2021**: MS ID#: MEDRXIV/2021/266786.

9 Weinstein S, Obuchowski NA, Lieber ML. Clinical Evaluation of Diagnostic Tests. *American Journal of Roentgenology* 2005; **184**: 14–9.

10 de Valpine P, Turek D, Paciorek CJ, Anderson-Bergman C, Lang DT, Bodik R. Programming with models: writing statistical algorithms for general model structures with NIMBLE. *Journal of Computational and Graphical Statistics* 2017; **26**: 403–13.

11 NIMBLE: MCMC, Particle Filtering, and Programmable Hierarchical Modeling. DOI:doi: 10.5281/zenodo.1211190.
