# Supplementary material for: Health inequities in SARS-CoV-2 infection, seroprevalence, and COVID-19 vaccination: Results from the East Bay COVID-19 study
Source: PLOS Glob Public Health. 2022 Aug 15;2(8):e0000647. doi: 10.1371/journal.pgph.0000647 (PMC10022102; doi:10.1371/journal.pgph.0000647)
Supplement: S5 File — (PDF) [file pgph.0000647.s017.pdf]

S5 File. Behaviors related to virus containment and mitigation.

1 Behaviors related to virus containment and mitigation

At each study round, participants were asked about physical distancing practices, recent close contacts with others, mask wearing, and behaviors and activities that might affect risk of SARS-CoV-2 or related outcomes. (See below for questions). To obtain a single binary measure of "mitigation behavior", we performed a latent class analysis (LCA) to classify participants into "high-risk" and "low-risk" behaviors classes, using the *poLCA* R package.[1–3] Sixty questions related to COVID-risk behaviors were grouped into 6 categories: 1) mask wearing, 2) leaving your home, 3) mode of transportation, 4) travel, 5) attendance at gatherings, and 6) occupational-related exposures (Table S-8). To create interpretable LCA classes (i.e., "high-risk" vs. "low-risk"), we first identified a subset of questions with each category that were the most representative of participant responses using similarity scores. For example, any masking wearing any of the queried situations (while at work, while traveling, while shopping, or while doing leisure activities outside) was representative of participants' mask wearing habits overall. A total of 15 variables across the six categories were identified using similarity scores and used for LCA classification. For the leaving home category, the most informative variables were leaving your home at all; leaving for either shopping, leisure, or exercise; and leaving for work, relative care, medical care, or another reason. Whether a participant used public or personal transportation was the most important feature of the transportation variables. Whether a participant traveled to countries outside the United States, states outside California, or counties outside of primary residence either since December 2019 or in the two weeks prior to completing the questionnaire were used

from the travel questions. Whether a participant attended any gathering or attended a gathering with more than 10 people were both retained for the gathering group. Finally, whether a participant or a member of their household had potential contact with persons infected with COVID-19 at work informed work exposures.

References

1 McLachlan, Jeffrey J, Thriyambakam Krishnan. The EM Algorithm and Extensions. New York: John Wiley & Sons, 1997.

2 Hagenaars JA, McCutcheon AL, editors. Applied Latent Class Analysis. Cambridge: Cambridge University Press, 2002 DOI:10.1017/CBO9780511499531.

3 Bandeen-Roche K, Miglioretti DL, Zeger SL, Rathouz PJ. Latent Variable Regression for Multiple Discrete Outcomes. *Journal of the American Statistical Association* 1997; **92**: 1375–86.
